# Landscape of target:guide homology effects on Cas9-mediated cleavage

**DOI:** 10.1093/nar/gku1102

**Published:** 2014-11-15

**Authors:** Becky Xu Hua Fu, Loren L. Hansen, Karen L. Artiles, Michael L. Nonet, Andrew Z. Fire

**Affiliations:** 1 Department of Genetics, Stanford University, Stanford, CA 94305, USA; 2 Department of Pathology, Stanford University, Stanford, CA 94305, USA; 3 Department of Anatomy and Neurobiology, Washington University School of Medicine, St. Louis, MO 63110, USA

## Abstract

To study target sequence specificity, selectivity, and reaction kinetics of *Streptococcus pyogenes* Cas9 activity, we challenged libraries of random variant targets with purified Cas9::guide RNA complexes *in vitro*. Cleavage kinetics were nonlinear, with a burst of initial activity followed by slower sustained cleavage. Consistent with other recent analyses of Cas9 sequence specificity, we observe considerable (albeit incomplete) impairment of cleavage for targets mutated in the PAM sequence or in ‘seed’ sequences matching the proximal 8 bp of the guide. A second target region requiring close homology was located at the other end of the guide::target duplex (positions 13–18 relative to the PAM). Sequences flanking the guide+PAM region had measurable (albeit modest) effects on cleavage. In addition, the first-base Guanine constraint commonly imposed by gRNA expression systems has little effect on overall cleavage efficiency. Taken together, these studies provide an *in vitro* understanding of the complexities of Cas9–gRNA interaction and cleavage beyond the general paradigm of site determination based on the ‘seed’ sequence and PAM.

## INTRODUCTION

Bacteria and archaea have evolved an adaptive immune system relying on a dynamic genomic structure called CRISPR (**c**lustered **r**egularly **i**nterspaced **s**hort **p**alindromic **r**epeats), an alternating genomic array of palindromic repeats and unique guide sequences (spacers). A set of CRISPR-associated (‘Cas’) proteins mediate three steps in CRISPR immunity. The first step is adaptation via the host's incorporation of foreign genetic material into the CRISPR array to make a spacer. Second, transcripts of the spacer and flanking repeats are processed into CRISPR RNAs (a.k.a. guide RNAs/gRNAs). Finally, the gRNAs are incorporated into an endonuclease complex, which utilizes guide RNA homology to potential DNA target sequences for cleavage. The CRISPR-Cas immune system has been adopted as a versatile and efficient platform for manipulation of DNA *in vitro* and for genome editing in various organisms (1–3). The breadth of efforts to adapt and apply Cas9 in biochemical manipulation and genome editing technology lend a substantial importance to a detailed understanding of requirements for gRNA::target DNA homology.

While Cas9 offers remarkable specificity for RNA-guided cleavage, mis-targeted cleavage events can result from the flexible requirement for guide-to-target complementarity. Recent studies in human and bacterial cells have begun to explore the *in vivo* and *in vitro* relationships between guide RNA sequence and binding/cleavage specificity (4). Notably, certain mutations (particularly in the protospacer adjacent motif (PAM) region) have been shown to greatly compromise Cas9 cleavage, as do certain single mismatches in a ‘seed’ region comprising the 10–12 bp closest to the PAM [4]. Despite these requirements, Cas9 gRNA-mediated cleavage can tolerate various mismatches in the 20 bp target region and in certain circumstances can cleave without the PAM sequence or with as many as five mismatches in the guide region [6,7,5]. While these studies have advanced our understanding of Cas9-gRNA mediated DNA cleavage, additional information remains of substantial value. Further knowledge of Cas9::gRNA homology specificity and cleavage can facilitate utilization of CRISPR as a tool but also can shed light on the mechanism in which an enzyme interrogates biological molecules. Here, we offer a quantitative high throughput analysis of homology specificity and cleavage efficiency between select gRNAs and polymorphic populations of target sequences.

## MATERIALS AND METHODS

As an overview, the Cas9 *in vitro* assay workflow used for this work consisted of:

1. Generation of a specific gRNA.
2. Generation of a random variant library containing homologous sequences with varying degrees of mismatch to the gRNA sequence.
3. Incubation of library with gRNA-programmed Cas9.
4. Amplification, sequencing and data analysis.

Below is a stepwise description of the workflow. Three random variant libraries and three guide RNAs were utilized in the work, with libraries designated ‘RVL’ (unc-22A RVL-1, unc-22A RVL-2 and ps4 RVL-3) and gRNAs designated unc-22A, unc-22A C11G and ps4. In addition to this workflow, additional assays carried out without amplification or sequencing are also described.

### Plasmid and constructs

#### gRNA expression vectors

gRNAs were made using vector pDR274 (Addgene #42250), (with thanks to Hwang *et al.* (5)), which carries a T7 promoter positioned upstream of the BsaI cassette for directional gRNA sequence insertion (5). To construct these vectors, appropriate primers were mixed in equal portions, heated to 100°C for 10 min, cooled to room temperature and cloned into the BsaI-digested and purified pDR274 vector.

### Cas9 protein

A C-terminal Nuclear-Localization-Signal-tagged *Streptococcus pyogenes* Cas9 ORF was polymerase chain reaction (PCR) amplified from MLM3613 (5) using oligonucleotides TAAAGGTCTCCCATGGATAAGAAATACTCAATAGGCTTAG and ATTTGGTCTCCAATTTCCTGCAGCTCCACCGC, digested with BsaI and inserted into the his6 expression vector pHO4d (NcoI EcoRI) (6). The plasmid was introduced into *E. coli* BL21(lambda-DE3) and 250 ml cultures were grown at 22.5°C to OD600 = 0.6, induced with 0.4 mM IPTG for 16 h. Cells were resuspended in 20 ml of buffer A [500mM NaCl, 20 mM Tris pH 8.0, 1 mM TCEP (tris(2-carboxyethyl)phosphine)] and sonicated on wet ice with ten 10 s bursts spaced at 1 min intervals with a microtip sonicator (Branson 185) to lyse the cells. The lysate was cleared by a 15 min 15000 g spin, and each supernatant incubated for 1 h at 4C with 1 ml of Ni-NTA agarose (Qiagen), rinsed two times with Buffer A and transferred to a disposable column. The matrix was washed extensively with Buffer A, then eluted with three 1 ml aliquots of 250 mM NaCl, 0.5 M imidazole, 20 mM Tris pH 8.0, 10% glycerol. The first two fractions were mixed, dialized twice with 100 volumes of 20 mM HEPES pH 7.5, 150 mM KCl, 0.5 mM DTT, 10% glycerol and frozen at -80°C. Yield was approximately 8 mg of Cas9 protein per 250 ml culture.

### Generation of random variant target library

Each random variant target library was generated through insertion of a mixed population of oligonucleotides into a plasmid vector (pHRL-TK, Promega). The initially single stranded oligonucleotides for the target library were designed to be converted to double stranded DNA using extension of a constant primer (AF-KLA-87) and cloned into the NotI and Acc65I digested pHRL-TK vector. The variable portion of the sequence was designed to be 35 nt (6 nt flanking on each side of a 23 nt target sequence). The two 6 nt flanking sequences and the N in the PAM sequence of the target were synthesized with custom mixes that were made up of 25% adenine (A), 25% thymine (T), 25% cytosine (C) or 25% guanine (G). The 22 nt sequence of unc-22A and ps4 were synthesized with custom mixes designed to produce a 10% variant rate at each position (3.3% of each variant base), although, the ps4 library has a unexpectedly high variant rate at two positions.

The initial diverse oligonucleotide pools were converted to double stranded DNA using NEB ThermoPol Taq polymerase and primer AF-KLA-87. Reaction tubes with 100 pM of each oligonucleotide in 25 ul dH_2_O and 2.5 ul of 10X Taq ThermoPol buffer (20 mM Tris-HCl, 10 mM KCl, 2 mM MgSO_4_, 10mM (NH_4_)_2_SO_4_, 0.1% Triton® X-100, pH 8.8) were placed in a beaker of boiling water for 5 min and removed from heat and allowed to cool at 45°C. The primer mix was then diluted by adding 5ul of 25 mM dNTPs, 210 ul of 1X Taq buffer and 10 ul of NEB Taq polymerase. An extension reaction was carried out for 15 min at 72°C, and was then halted by addition of 180 ul of (1.9 NH_4_OAc, 19 mM EDTA, 0.38% SDS), and 1.5 ug of GlycoBlue. Following extractions with phenol/chloroform (1:1) and chloroform, and ethanol precipitation, the resulting material was resuspsended in 40 ul of TE, digested with NEB NotI+Acc65I, purified following electrophoresis on a 1.5% agarose gel and ligated into the NotI and AccI65 digested recipient vector using NEB Quick ligation. Ligation mixture was transformed into library efficiency DH5α cells (Invitrogen) and plated on pre-warmed 15 cm plates. Colonies were grown overnight at 37°C then moved and incubated at 30°C for 48 h. Approximately 5000 colonies were scraped off of agar plates into 50 ml of 2XTY for each library. The 50 ml of cells were pelleted and prepped using Qiagen Midi plasmid prep kit. Each 50 ml of prepped plasmid DNA established one unique random variant library. In this study, multiple variant libraries were created for both the unc-22A and ps4 targets.

### Generation of guide RNA

Each gRNA plasmid was cleaved with restriction enzyme DraI, followed by gel purification of the resulting T7::gRNA fragment. RNA was synthesized using the Ambion Megascript system (10ul reaction; 3–6 h at 37°C) followed by treatment with Megascript DNase (15 min at 37°C). gRNAs from the Megascript protocol were resuspended in 15–20 ul TE. Each batch of gRNA was titrated for optimal cleavage before use in the Cas9 *in vitro* assay. Optimal cleavage was determined by titrating gRNA with the approximately 80 pmoles of perfect target sequence (20–25 ng of total DNA). The dilution with the maximum cleavage for each batch was used in the Cas9 *in vitro* assay. Optimal cleavage levels corresponded to 15–35 ng (0.33–0.76 pmoles) RNA per reaction.

### Cas9 *in vitro* assays and sequencing

Cas9 *in vitro* assays were performed in 10 ul reactions, unless specified otherwise, with Cas9 protein pre-incubated with gRNA, buffer (20 mM Tris-HCl, 10 mM (NH_4_)_2_SO_4_, 10 mM KCl, 2 mM MgSO_4_, 0.1% Triton® X-100, pH 8.8), dH_2_O, and random variant target library. Following incubation at 37°C, reactions were stopped by flash freezing in dry ice. Subsequent protein removal was carried out immediately after thawing: 200 ul of proteinase K buffer (0.1M NaCl, 0.1M TRIS pH 8.4, 0.1M EDTA, 1% SDS) and 20 uls of proteinase K (20 mg/ml) were added and the reaction allowed to incubate for 3 h at 60°C. After adding 190 ul TE, 1 ul of 1.5 ug of GlycoBlue, 2.5 ug of yeast tRNA and 40 ul of saturated ammonium acetate, samples were extracted with phenol/ chloroform (1:1) and chloroform, ethanol precipitated, and resuspended in 10 ul of TE.

PCR was used to amplify the retained sequences and add Illumina-compatible adaptors for sequencing. This included a first round (15 cycles) with short primers AF-KLA-136 and AF-KLA-140, and a second round (6 cycles) with longer primers bearing index sequences and flow cell anchors for sequencing and subsequent analysis (Supplementary Tables S1 and S2). To investigate possible PCR bias in the Cas9 *in vitro* cleavage assay, various experiments using the same approach with varying number of cycles were performed, in addition to a PCR-free Cas9 *in vitro* cleavage assay (Supplementary Figures S8 and S9). Beyond a slight difference in overall cleavage estimates for large and small numbers of PCR cycles, the general trends and patterns were consistent between various assay protocols and within various experiments. All libraries were sequenced using the 50 cycle Illumina Miseq (100 cycle single-read) platforms. A list of experiments, experimental conditions, and sequencing run IDs is reported in the supplemental material (Supplementary Table S2).

### Cas9 in vitro non-PCR assay

Cas9 *in vitro* experiments with non-PCR-based measurement of cleavage were performed for validation (Supplementary Figures S8 and S10). To obtain a rough assessment testing the validity of PCR-based measurement for cleavage of variants, we carried out a number of assays in which cleavage of plasmids with specific variants was analyzed directly with gel electrophoresis. The tested plasmids carry specific variants and have been engineered to generate separable restriction fragments cut with NEB PvuI-HF. The fragments, if cleaved, can be visualized by band migration changes. See Supplementary Figures S8 and S10 for sizes and examples of the Cas9 *in vitro* non-PCR assay.

### Computational methods

#### Calculation of retention and normalization

We use the log2 of retention in the PCR pool as a measure of target cleavage by Cas9, with a retention score calculated for each sequence in each experiment as described below. The retention scores provide a framework to compare cleavage efficiencies for each of the sequences present in the respective libraries.

For each sequence ‘X’:

\begin{eqnarray*} && {\rm Retention}[{\rm X}]\nonumber\\ && = {\rm log}2({\mathop{\rm Re}\nolimits} {\rm presentation}_{\rm X} [{\rm Cleaved}\;{\rm Library}]\nonumber\\ && /{\mathop{\rm Re}\nolimits} {\rm presentation}_{\rm X} [{\rm Uncleaved}\;{\rm Library}]) \end{eqnarray*}

Representation_x_ in a library gives the ratio between:

\begin{eqnarray*} && [{\rm Instances}\;{\rm of}\;{\rm sequence}\;{\rm X}\;{\rm in}\;{\rm the}\;{\rm library}]\nonumber\\ && /[{\rm Instances}\;{\rm of}\;{\rm a}\;{\rm reference}\;{\rm population}\;{\rm in}\;{\rm the}\;{\rm library}] \end{eqnarray*}

As reference populations we used either an aggregate of all sequences with 4–7 mismatches to the original trigger, or (for the unc-22A library) an ‘internal control’ population comprising a subset of plasmids from the protospacer 4 library. Comparable results were obtained with these two references for normalization (for figures in this manuscript, we have used the 4–7 mismatched sequences as a reference). Only sequences that had at least 50 read counts in the reference library population were analyzed for cleavage.

## RESULTS AND DISCUSSION

### Cas9 in vitro homology specificity assay

**Figure 1.**
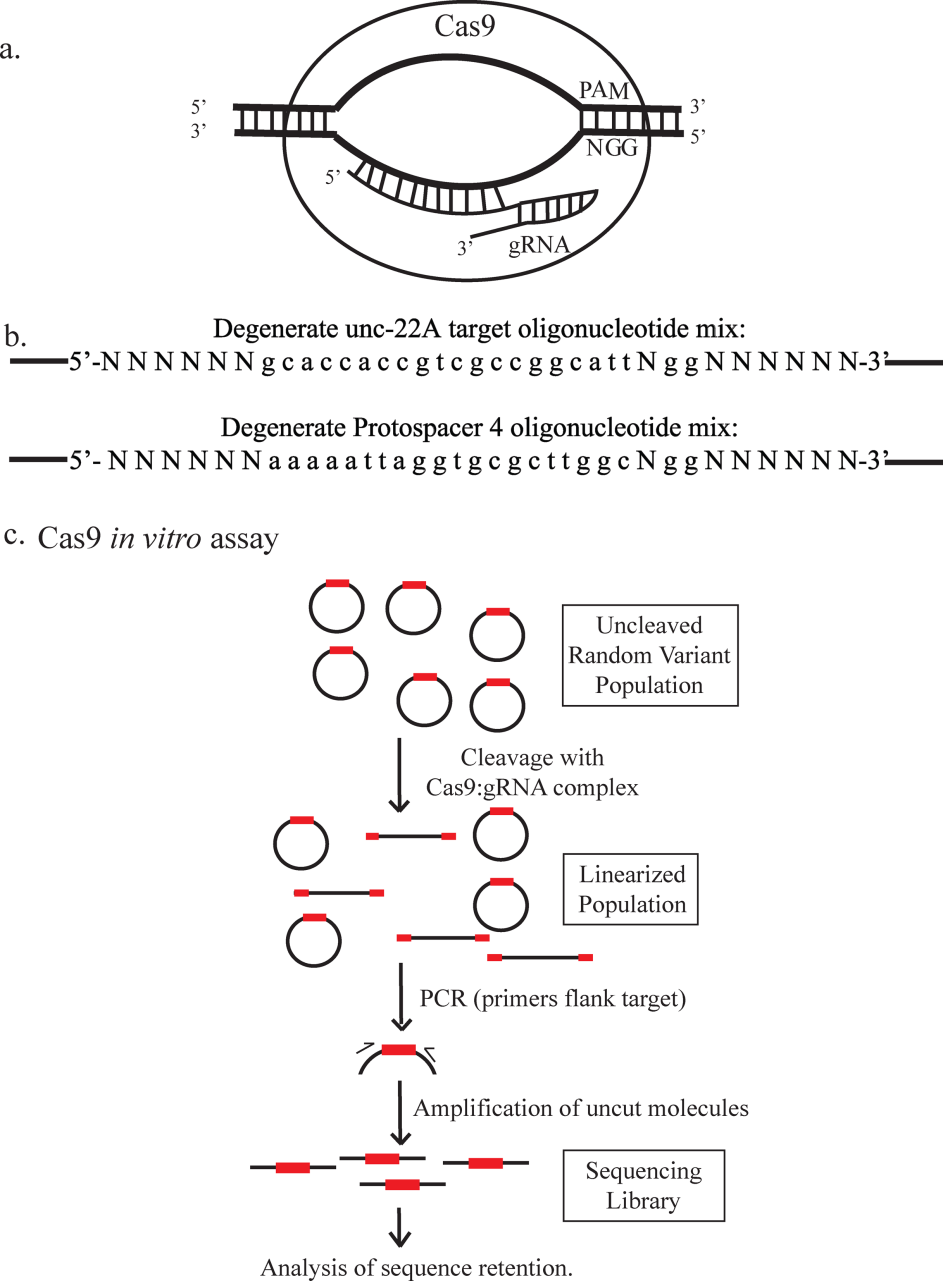
Assaying a diverse population of potential targets for Cas9 cleavage. (**a**) Schematic of target recognition by Cas9 (redrawn from Pattanayak *et al.* (8)). Cas9 uses 20 bp of gRNA homology and PAM to recognize and target DNA and cleave both strands of substrate. (**b**) Target sequences assayed using random variant libraries. From the 5′ end the bases are indexed starting at −5 and ending with 29 (left to right). Positions 21–23 correspond to the PAM (NGG). Positions 11–20 correspond to the seed region. For both guides, the 0th position contained a guanine added on by the gRNA expression vector. c. Cas9 *in vitro* assay. Each random variant library was incubated with Cas9:gRNA complexes. PCR was used to amplify uncut Cas9 target regions. The resulting amplicons were subjected to high-throughput DNA sequencing and computational analysis described in Materials and Methods.

To quantitatively measure cleavage efficiency of a single gRNA, we created a population of random variant target sequences for two gRNA targets. The targets used were ‘unc-22A’, [a sequence from the well-characterized *unc-22* gene of *Caenorhabditis elegans*], and ‘protospacer 4′ (ps4), a previously characterized sequence from a natural spacer from *S. pyogenes* MGAS10750 (7,9). Using custom mixtures of oligonucleotide precursors for each base during chemical synthesis, a set of polymorphic target libraries (‘Random Variant Libraries’ a.k.a. RVL) were designed to have a baseline variation rate at each position. On each side of the gRNA homology and PAM regions, 6 bp of random sequence were added. The first base of intended gRNA homology is designated base 1 (Figure 1b). The entire 35 bp random variant library mixture was cloned into a standard plasmid vector (pHRL-TK). Several thousand colonies from plates were washed in pools and prepared by standard plasmid preparation methods. The complexity of the libraries were estimated based on Illumina sequencing of the uncut libraries and filtering for minimum representation (n> = 50 counts). Approximately 1500–3000 unique species were obtained in the unc-22A libraries and 5000 unique sequences in the ps4 library (see Materials and Methods).

To assay cleavage, purified Cas9 was first incubated with gRNA (7), followed by incubation with the variant library for various time points and under various conditions (Figure 1c). DNA template is among the conditions varied in the experiments (see Supplementary Table S2 for a list of experiments). After protein removal (see Materials and Methods), flanking sequences outside of the target region are used for PCR amplification and plasmid cleavage was measured through loss of PCR products that span the region of interest. A set of perfectly matched targets and highly mutated versions present in the random variant library served as internal positive and negative controls, respectively.

A log retention score for each sequence in each experiment was calculated by quantifying the representation of each sequence before and after addition of the Cas9 protein. Two approaches were used for normalization: first, we used a population of ps4 targets ‘spiked’ into the library as an uncleaved control; second, we used a population of unc-22A targets with large numbers of variations from the perfect target (between 4 and 7), and hence likely limited cleavage, if any. Equivalent results were obtained with these two normalization approaches (see Computational Methods for details). Retention scores are expressed as the log2 of the normalized ratio, so that a more negative retention score indicates efficient cleavage of substrate while a less negative score indicates less cleavage. Templates which are uncleaved will yield a retention score at or near zero. Comparisons between multiple experiments indicate strong correlation between independent retention measurements (Supplementary Figures S6 and S7).

### Effects of single base variants on cleavage efficiency

**Figure 2.**
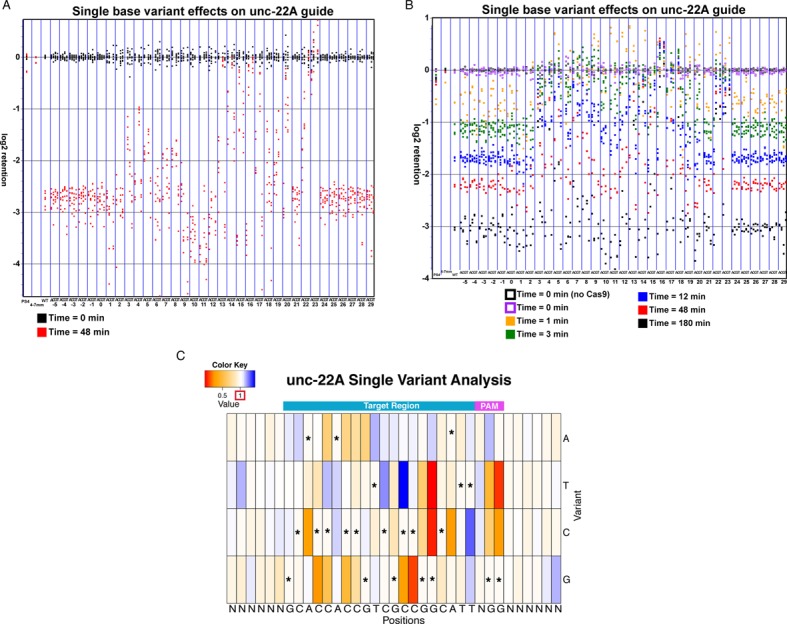
Cleavage results for two independent unc-22A random variant libraries. (**A**) Each variant is indicated by a dot noting differences from the canonical sequence. Retention at various time points following Cas9 addition is depicted as a dot colored according to time point. For the target region and constrained PAM nucleotides (positions 1–20 and 22–23), each dot represents median retention amongst matching library sequences carrying the variant of interest (either A,C,G or T) and distinct flanking sequences. For the flanking sequence and the N in the PAM, each dot represents median retention for library species with fully matched target/PAM regions and with the indicated flanking position constrained to the indicated base. Only species with > = 50 reads in the control (uncut) library were considered in calculating this median. Two negative (labeled PS4) and one positive (labeled WT) controls are shown at the left. Sequences with 4–7 mismatches in the target region, and sequences from a ps4 ‘spike in’ behave in aggregate as similar ‘uncut’ pools, while a median from all sequences that match the guide and PAM sequences perfectly provides a positive control. The retention scores shown are from experiments done on unc-22A random variant library-2 (AF_SOL_516, using 0.2 ug of enzyme per reaction). Negative retention scores indicate increasing cleavage, while a retention score of zero corresponds to samples whose cleavage is comparable to the pool of highly mutated sites (with 4–7 mutations) and to trace amounts of unrelated (protospacer 4) DNA. Some samples showed a slightly positive retention score, indicating a lack of cleavage with either noise or consistent PCR effects leading to a slight over-representation in the post-cleavage sample. (**B**) Detailed time course experiments for unc-22A random variant library-1. Methods of analysis were as in Figure 2A. The retention scores shown are from experiment AF_SOL_515, using 0.2 ug of enzyme per reaction. (**C**) Heat map summarizing cleavage for single variants for 180 min time point experiments shown in part b. The heat map shows (median variant retention)/(median WT retention). Boxes with asterisks indicate the WT base. Values with the score of ∼1 (white) are variants with retentions that do not greatly deviate from the median for all perfectly matched (guide+PAM) targets. Variants with values that are above 1 (blue) correspond to variants where read counts indicated a more efficient Cas9-based depletion than WT. Variants with values around ∼0 (red) correspond to variants that either do not cleave or cleave very inefficiently. Retention scores shown are from experiment AF_SOL_515, using 0.2 ug of enzyme per reaction.

**Figure 3.**
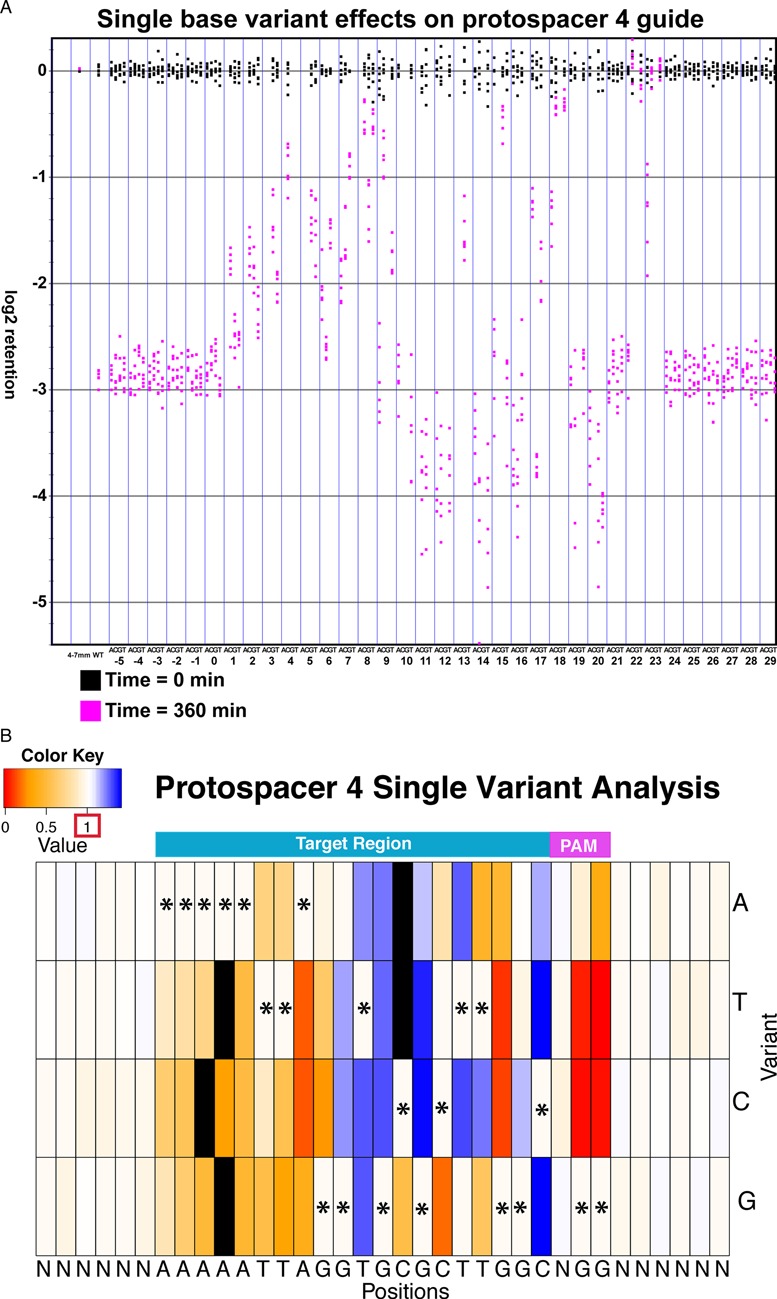
Cleavage results for the protospacer 4 (ps4 (7)) library analysis. Methods of analysis from Figure 2 were used to analyze the ps4 variant library. A reference population used for normalization with ps4 libraries consists of sequences with 4–7 mismatches. The log retention scores shown are from experiment AF_SOL_513, using 0.2 ug of enzyme per reaction. (**A**) Cleavage results for ps4. (**B**) Heat map *in vitro* results for ps4. Black boxes represent variants that were not assayed due to insufficient representation in the library.

Here, we present the effects of single variants for the unc-22A and the ps4 guides. We note that, similar to past studies, we find different gRNAs produced unique off target profiles (8). We highlight the similarities between the unc-22A and ps4 guides and acknowledge that there is variation between the cleavage profiles that are specific to each target. Single base variants affected Cas9 cleavage to varying degrees, depending on both the position and the nature of mutation (Figures 2 and 3, Supplementary Figures S1 and S2). As in previous studies (9–12), we found a strong influence of the PAM sequence on Cas9 cleavage. Single-base transversions (G to T or C) at either position of the PAM greatly decrease cleavage, while single transitions (G to A) can be moderately tolerated (at the first position in unc-22A and at both in ps4), with a decrease in cleavage activity (Figures 2 and 3, Supplementary Figures S1 and S2). In addition, as in previous studies, we found that not all base changes in the seed region have the same effect on cleavage activity (12).

In the seed region, certain variants at positions 13, 14, 15, 16, 17 and 18 substantially decreased cleavage efficiency, while only minimal effects were observed at bases 19 and 20 (Figures 2 and 3, Supplementary Figures S1 and S2). As might be expected from effects on duplex structure, transversion variants had much more severe effects than transitions. Not all positions within the seed region were equal in their effects. Variants at the end of the guide homology closest to the PAM region could decrease efficiency but matches at these positions were not critical for cleavage. Additional non-PCR cleavage assays were carried out, with verification that the unc-22A PAM proximal variant, T20A, was cleaved while a variant 5 bp away from the PAM, G16T, failed to cleave (Supplementary Figure S10).

We observed a second critical region of gRNA homology close to the 5′ end of the target sequence for both libraries. Variants at positions 3, 4 and 5 were particularly notable in their substantial loss of cleavage activity for both targets (Figures 2 and 3, Supplementary Figures S1 and S2). Although it has been reported that the regions beyond the seed region are less important for cleavage, a contribution of such sequences toward effective cleavage is evident for both the unc-22A and ps4 guides (13).

We note that some variants at positions 9,10 and 11 led to reproducible higher cutting efficiency for both the unc-22A and ps4 target sequences with extended reaction time (Figures 2 and 3, Supplementary Figures S1 and S2). While mechanistic information regarding the improved cleavage of these targets remains to be obtained, their improved cleavage supports a model in which overly tight pairing of guide and target strands may decrease target cleavage activity. Although we have not tested the efficacy of the centrally mutated guide RNAs *in vivo*, it has been shown that bulges between the gRNA and the DNA target caused by insertions and deletions (yielding a structure potentially similar to the mismatches described here) can lead to higher genome editing efficiency *in vivo* (14).

**Figure 4.**
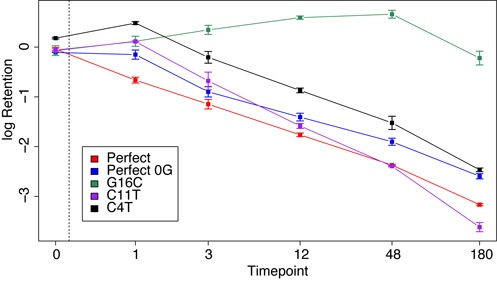
Kinetics of Cas9 cleavage for exemplary variant categories. Graph depicts the median retention for each exemplary variant as a function of time for the unc-22A random variant library (AF_SOL_515). The error bars represent the standard deviation of the median retentions for each exemplary variant at the specific time point.

Our library design offered an additional ability to characterize flanking sequence effects on target cleavage. One flanking position with a modest effect on cleavage was at base position 0, just beyond the homology between gRNA and target DNAs. Target DNAs with a cytosine (C) in the 0th position produced a modest decrease in cleavage efficiency (Figures 2–4, Supplementary Figures S1 and S2). We note that the synthetic gRNAs produced with T7 polymerase for these assays have an additional guanine (G) on the 5′ end, with the interaction of the 5′ G with the 0th position of the target DNA potentially stabilizing (and thus perturbing) specific intermediate structure(s) in Cas9 cleavage. The 5′ G had reproducible modest effects on cleavage efficiency for the unc-22A libraries, with templates carrying a matching base at this position having slightly reduced cleavage. Operationally, the number of gRNAs available for any given site of interest increases once we eliminate the requirement of the G at the first gRNA base.

Similar to past studies, we observed target specific cleavage profiles and some guide-specific features of off-target profiles in the unc-22A and ps4 random variant library analysis. Thus, for example, sequences toward the 5′ end had more profound effects on the ps4 guide than on the unc-22A guide (Figures 2 and 3, Supplementary Figures S1 and S2). Although universal rules for Cas9 cleavage is of interest for many scientists, it is evident that from this study that Cas9 cleavage specificity is extremely target specific. Further analysis of additional targets would be needed to determine the basis for such diversity.

### Cas9 cleavage kinetics and efficiency

The kinetics of Cas9 cleavage suggest a complex reaction in which the enzyme may ‘hold onto’ the DNA even in the absence of cleavage (13). We observed rapid cleavage of a small fraction of DNA targets, followed by slower cleavage of the remainder. We observed different cleavage kinetics for distinct targets, with diversity both among perfect match sequences with diverse flanking regions, and for variants within the target homology.

Figure 4 shows median retention versus time for several exemplary variants. Some targets, exemplified by variant (C4T), lag behind the median for perfect match controls at early time points, catching up later (additional plots can be found in supplemental: Figure S3). Sequences with enhanced cleavage relative to perfect match controls tend to exhibit their apparent advantage at later time points, exemplified by variant at position 11 C to T (C11T). A subset of targets showed impaired cleavage in all time points, exemplified by variant at position 16 G to C (G16C). These observations highlight the time-dependent nature of sequence effects on Cas9 cleavage efficiency.

To further explore the unusually weak requirement for sequence homology at position 11, we carried out a parallel analysis of cleavage with a mutated version of the unc-22A guide (unc-22A mutated to C11G). This guide RNA produced similar results with the assay (Supplementary Figures S8 and S9), including a similar horseshoe-shaped pattern in mutational effects with the library.

### Effects of double variants on cleavage efficiency

**Figure 5.**
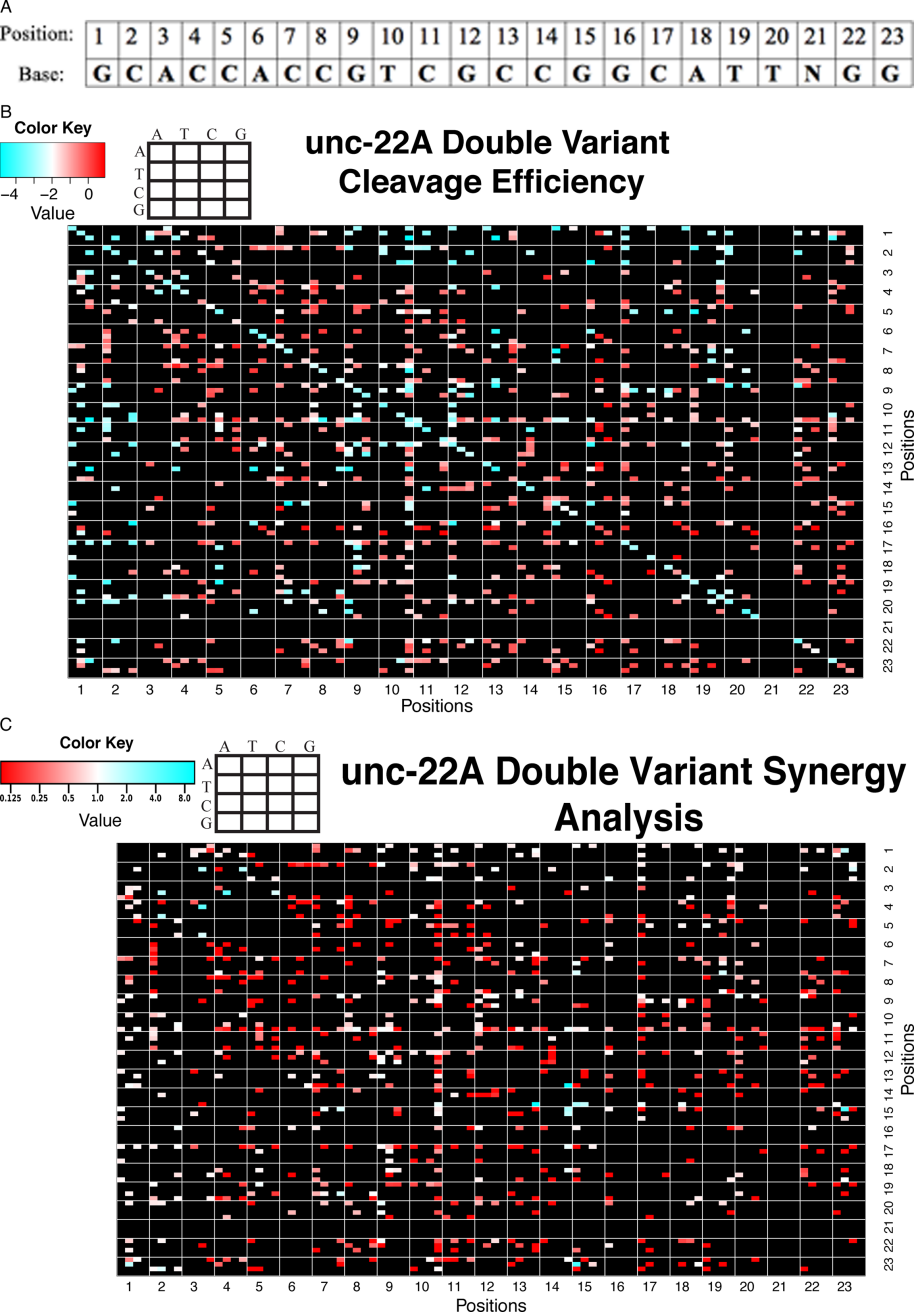
Double variant analysis for unc-22A random variant library (AF_SOL_515). Each position of the target sequence is a box with a 4×4 grid labeled ‘ATCG’. The 4×4 grid denotes the variant and the positions of the double variants (shown in the figure legend). Because transitions and transversions have diverse effects on cleavage efficiency, the data are represented in a detailed heat map (92×92). Only the 23 bp target sequence of the gRNA is shown in this heat map (positions 1–23). Only species with > = 50 reads in the control (uncut) library were considered. **(A**) Table showing bases of gRNA and corresponding position (for reference to heat map positions). (**B**) Detailed double variant heat map of log retentions. Each box represents the median log retention of the double variant of interest. For reference, the diagonal provides median log retention of the single variants. Red colors correspond to variants that do not cleave efficiently. Blue colors correspond to variants with highly efficient cleavage. (**C**) Detailed double variant synergy heat map. Each box represents the value of the double variant divided by the corresponding the multiplication of the single variants: (LR_M12_/LR_WT_) / [(LR_M1_/LR_WT_) * (LR_M2_/LR_WT_)]. Single mutants with no detectable cleavage (no significant difference in representation between cleaved and uncleaved) were not considered in this analysis, as a synergy value cannot generally be calculated. A synergy value of 1 corresponds to double variants whose kinetic influence on the reaction rate is the product of the influences of the two single variants. Synergy values below 1 indicate negative synergistic effects of variants, while values above 1 indicate a higher residual cleavage activity on the double variant that would be expected from the individual effects of the single variants.

The depth of sequencing data obtained from the unc-22A random variant library provided data on a subset of the possible double variants. Many double variant combinations had severe effects on cleavage, while a few remained sensitive to cleavage (Figure 5, Supplementary Figures S4 and S5). A few specific features of the double variant analysis are worthy of note.

- Variants that lie in some non-critical regions (such as the ultimate PAM-distal base of the 20 bp target) (position 1) had little if any contribution in double variant combinations.
- G-to-A variants in the PAM region and in specific seed regions, although tolerated as single variants, result in unexpectedly severe effects on cleavage in double variant combinations. The AG PAM with a perfect target sequence is cleaved but any double variant combination ablated cleavage.

## CONCLUSION

In this study, we analyzed the effect of variants on the target sequence on cleavage efficiency of a popular RNA-guided endonuclease (1,2). Various studies have begun to explore the gRNA–DNA interaction and its effects on Cas9 specificity (8,10,11). Here, we analyze Cas9 homology specificity by investigating cleavage by three Cas9::gRNA complexes (unc-22A, unc22-A C11G and ps4) in the context of diverse populations of targets. Our findings corroborate findings from previous studies. Based on the targets and gRNAs in our study, we find that Cas9 specificity and cleavage is complex and can have diverse cleavage specificity depending on the target. Although this study is able to give insight on Cas9 cleavage behavior *in vitro*, we note that it is in the context of challenging Cas9 with thousands of targets that are similar to the target to which the gRNA is programmed. In an *in vivo* situation, Cas9 is challenged with an entire genome including hindrances such as nucleosomes and chromatin, with potential gRNA targets being much rarer. Our analysis of select gRNAs provides both an overall map of sequence requirements for cleavage and detailed data on thousands of target variants that we hope will be of value for both *in vitro* applications of the enzyme and further modeling and analysis to extend the body of knowledge on the CRISPR/Cas system.

## SRA ACCESSION NUMBER

GSE58426.

## SUPPLEMENTARY DATA

Supplementary Data are available at NAR Online.

SUPPLEMENTARY DATA
