# Supplementary material for: Landscape of target:guide homology effects on Cas9-mediated cleavage
Source: Nucleic Acids Res. 2014 Nov 15;42(22):13778–87. doi: 10.1093/nar/gku1102 (PMC4267615; doi:10.1093/nar/gku1102)
Supplement: SUPPLEMENTARY DATA [file supp_42_22_13778__index.html]

Landscape of target: guide homology effects on Cas9-mediated cleavage — Landscape of target:guide homology effects on Cas9-mediated cleavage — SUPPLEMENTARY DATA 

# Landscape of target:guide homology effects on Cas9-mediated cleavage

## SUPPLEMENTARY DATA

**Files in this Data Supplement:**

- SUPPLEMENTARY DATA
