# Supplementary material for: Landscape of target:guide homology effects on Cas9-mediated cleavage
Source: Nucleic Acids Res. 2014 Nov 15;42(22):13778–87. doi: 10.1093/nar/gku1102 (PMC4267615; doi:10.1093/nar/gku1102)
Supplement: SUPPLEMENTARY DATA [file supp_gku1102_nar-01366-h-2014-File003.docx]

**Supplementary Material:**

**Table S1**: Oligonucleotide sequences.

| **Oligo name:** | **Oligo sequence (5’ to 3’):** | **Extra information:** |
| --- | --- | --- |
| AF-KLA-67 | AATGATACGGCGACCACCGAGATCTACACTATAGCCTACACTCTTTCCCTACACGACGCTCTTCCGATCT | \|D501 Truseq HT for Luciferase DNA libraries forward \| |
| AF-KLA-68 | AATGATACGGCGACCACCGAGATCTACACATAGAGGCACACTCTTTCCCTACACGACGCTCTTCCGATCT | \|D502 Truseq HT for Luciferase DNA libraries forward \| |
| AF-KLA-69 | AATGATACGGCGACCACCGAGATCTACACCCTATCCTACACTCTTTCCCTACACGACGCTCTTCCGATCT | \|D503 Truseq HT for Luciferase DNA libraries forward \| |
| AF-KLA-70 | AATGATACGGCGACCACCGAGATCTACACGGCTCTGAACACTCTTTCCCTACACGACGCTCTTCCGATCT | \|D504 Truseq HT for Luciferase DNA libraries forward \| |
| AF-KLA-71 | AATGATACGGCGACCACCGAGATCTACACAGGCGAAGACACTCTTTCCCTACACGACGCTCTTCCGATCT | \|D505 Truseq HT for Luciferase DNA libraries forward \| |
| AF-KLA-72 | AATGATACGGCGACCACCGAGATCTACACTAATCTTAACACTCTTTCCCTACACGACGCTCTTCCGATCT | \|D506 Truseq HT for Luciferase DNA libraries forward \| |
| AF-KLA-73 | AATGATACGGCGACCACCGAGATCTACACCAGGACGTACACTCTTTCCCTACACGACGCTCTTCCGATCT | \|D507 Truseq HT for Luciferase DNA libraries forward \| |
| AF-KLA-74 | AATGATACGGCGACCACCGAGATCTACACGTACTGACACACTCTTTCCCTACACGACGCTCTTCCGATCT | \|D508 Truseq HT for Luciferase DNA libraries forward \| |
| AF-KLA-124 | CAAGCAGAAGACGGCATACGAGATCGAGTAATGTGACTGGAGTTCAGACGTGTGCTCTTCCGATCGCAGTAATTCTAGAGGTACC | [D701] REV primer for DNA libraries |
| AF-KLA-125 | CAAGCAGAAGACGGCATACGAGATTCTCCGGAGTGACTGGAGTTCAGACGTGTGCTCTTCCGATCGCAGTAATTCTAGAGGTACC | [D702] REV primer for DNA libraries |
| AF-KLA-126 | CAAGCAGAAGACGGCATACGAGATAATGAGCGGTGACTGGAGTTCAGACGTGTGCTCTTCCGATCGCAGTAATTCTAGAGGTACC | [D703] REV primer for DNA libraries |
| AF-KLA-127 | CAAGCAGAAGACGGCATACGAGATGGAATCTCGTGACTGGAGTTCAGACGTGTGCTCTTCCGATCGCAGTAATTCTAGAGGTACC | [D704] REV primer for DNA libraries |
| AF-KLA-128 | CAAGCAGAAGACGGCATACGAGATTTCTGAATGTGACTGGAGTTCAGACGTGTGCTCTTCCGATCGCAGTAATTCTAGAGGTACC | [D705] REV primer for DNA libraries |
| AF-KLA-129 | CAAGCAGAAGACGGCATACGAGATACGAATTCGTGACTGGAGTTCAGACGTGTGCTCTTCCGATCGCAGTAATTCTAGAGGTACC | [D706] REV primer for DNA libraries |
| AF-KLA-130 | CAAGCAGAAGACGGCATACGAGATAGCTTCAGGTGACTGGAGTTCAGACGTGTGCTCTTCCGATCGCAGTAATTCTAGAGGTACC | [D707] REV primer for DNA libraries |
| AF-KLA-131 | CAAGCAGAAGACGGCATACGAGATGCGCATTAGTGACTGGAGTTCAGACGTGTGCTCTTCCGATCGCAGTAATTCTAGAGGTACC | [D708] REV primer for DNA libraries |
| AF-KLA-132 | CAAGCAGAAGACGGCATACGAGATCATAGCCGGTGACTGGAGTTCAGACGTGTGCTCTTCCGATCGCAGTAATTCTAGAGGTACC | [D709] REV primer for DNA libraries |
| AF-KLA-133 | CAAGCAGAAGACGGCATACGAGATTTCGCGGAGTGACTGGAGTTCAGACGTGTGCTCTTCCGATCGCAGTAATTCTAGAGGTACC | [D710] REV primer for DNA libraries |
| AF-KLA-134 | CAAGCAGAAGACGGCATACGAGATGCGCGAGAGTGACTGGAGTTCAGACGTGTGCTCTTCCGATCGCAGTAATTCTAGAGGTACC | [D711] REV primer for DNA libraries |
| AF-KLA-135 | CAAGCAGAAGACGGCATACGAGATCTATCGCTGTGACTGGAGTTCAGACGTGTGCTCTTCCGATCGCAGTAATTCTAGAGGTACC | [D712] REV primer for DNA libraries |
| AF-BXHF-5 | CATGGTACCNNNNNN ccNaatgccggcgacggtggtgcNNNNNNAGATCGGAAGAGCGTGCGGCCGCAAC | Unc-22A target sequence randomized library |
| AF-BXHF-6 | CATGGTACCNNNNNN ccNgccaagcgcacctaattttt NNNNNNAGATCGGAAGAGCGTGCGGCCGCAAC | Protospacer 4 target seq randomized library |
| AF-KLA-87 | AGTTGCGGCCGCACGC | Short primer to make AF-BXHF-5 and AF-BXHF-6 double stranded |
| AF-KLA-136 | CCTACACGACGCTCTTCCGATCT | Short forward primer for PCR1 |
| AF-KLA-140 | ATCGCAGTAATTCTAGAGGTACC | Short reverse primer for PCR 2 |

**Table S2**: List of bulk Cas9 cleavage experiments and corresponding sequence datasets. Experiments can be identified by on SRA by sample ID and Illumina Run ID. For example, 2a-lo_S9_L001_R1_001_AF_SOL_513.fastq corresponds to the first entry of Table S2.

SRA accession number: GSE58426

| **Target Library:** | **Incubation time:** | **Sequencing platform:** | **Target and gRNA:** | **Amount**  **of Cas9 (ug):** | **Total DNA:** | **Sequencing Primers (sample ID):** | **Illumina Run ID:** |
| --- | --- | --- | --- | --- | --- | --- | --- |
| Proto4 RVL-2 | 180 min | MiSeq | Proto4 | 0.2 | ~10ng | AF-KLA-126, 67  (2a) | AF_SOL_513 |
| Proto4 RVL-2 | 180 min | MiSeq | Proto4 | 0.2 | ~10ng | AF-KLA-126, 68  (2b) | AF_SOL_513 |
| Proto4 RVL-2 | 180 min | MiSeq | Proto4 | 0.2 | ~10ng | AF-KLA-126, 69  (2c) | AF_SOL_513 |
| Proto4 RVL-2 | 180 min | MiSeq | Proto4 | 0.2 | ~10ng | AF-KLA-126, 70  (2d) | AF_SOL_513 |
| Proto4 RVL-2 | 180 min | MiSeq | Proto4 | 0.2 | ~10ng | AF-KLA-126, 72  (2f) | AF_SOL_513 |
| Proto4 RVL-2 | 180 min | MiSeq | Proto4 | 0.2 | ~10ng | AF-KLA-126, 73  (2g) | AF_SOL_513 |
| Proto4 RVL-2 | 180 min | MiSeq | Proto4 | 0.2 | ~10ng | AF-KLA-126, 74  (2h) | AF_SOL_513 |
| Proto4 RVL-2 | 180 min | MiSeq | Proto4 | 0.2 | ~10ng | AF-KLA-127, 67  (3a) | AF_SOL_513 |
| Proto4 RVL-2 | 180 min | MiSeq | Proto4 | 0.2 | ~10ng | AF-KLA-127, 68  (3b) | AF_SOL_513 |
| Proto4 RVL-2 | N/A | MiSeq | Proto4 | 0 | ~10ng | AF-KLA-127, 69  (3c) | AF_SOL_513 |
| Proto4 RVL-2 | N/A | MiSeq | Proto4 | 0 | ~10ng | AF-KLA-127, 70  (3d) | AF_SOL_513 |
| Proto4 RVL-2 | N/A | MiSeq | Proto4 | 0 | ~10ng | AF-KLA-127, 71  (3e) | AF_SOL_513 |
| Proto4 RVL-2 | N/A | MiSeq | Proto4 | 0 | ~10ng | AF-KLA-127, 72  (3f) | AF_SOL_513 |
| Proto4 RVL-2 | N/A | MiSeq | Proto4 | 0 | ~10ng | AF-KLA-127, 73  (3g) | AF_SOL_513 |
| Proto4 RVL-2 | N/A | MiSeq | Proto4 | 0 | ~10ng | AF-KLA-127, 74  (3h) | AF_SOL_513 |
| unc-22A RVL-2 | N/A | MiSeq | unc-22A | 0 | ~10ng | KLA-127, 69  (3c) | AF_SOL-514 |
| unc-22A RVL-2 | N/A | MiSeq | unc-22A | 0 | ~10ng | AF-KLA-127, 70  (3d) | AF_SOL-514 |
| unc-22A RVL-2 | N/A | MiSeq | unc-22A | 0 | ~10ng | AF-KLA-127, 71  (3e) | AF_SOL-514 |
| unc-22A RVL-2 | N/A | MiSeq | unc-22A | 0 | ~10ng | AF-KLA-127, 72  (3f) | AF_SOL-514 |
| unc-22A RVL-2 | N/A | MiSeq | unc-22A | 0 | ~10ng | AF-KLA-127, 73  (3g) | AF_SOL-514 |
| unc-22A RVL-2 | N/A | MiSeq | unc-22A | 0 | ~10ng | AF-KLA-127, 74  (3h) | AF_SOL-514 |
| unc-22A RVL-2 | N/A | MiSeq | unc-22A | 0 | ~10ng | AF-KLA- 129, 67  (4a) | AF_SOL-514 |
| unc-22A RVL-2 | N/A | MiSeq | unc-22A | 0 | ~10ng | AF-KLA- 129, 68  (4b) | AF_SOL-514 |
| unc-22A RVL-2 | 3 min | MiSeq | unc-22A | 0.2 | ~10ng | AF-KLA- 129, 69  (4c) | AF_SOL-514 |
| unc-22A RVL-2 | 3 min | MiSeq | unc-22A | 0.2 | ~10ng | AF-KLA- 129, 70  (4d) | AF_SOL-514 |
| unc-22A RVL-2 | 3 min | MiSeq | unc-22A | 0.2 | ~10ng | AF-KLA- 129, 71  (4e) | AF_SOL-514 |
| unc-22A RVL-2 | 3 min | MiSeq | unc-22A | 0.2 | ~10ng | AF-KLA- 129, 72  (4f) | AF_SOL-514 |
| unc-22A RVL-2 | 3 min | MiSeq | unc-22A | 0.2 | ~10ng | AF-KLA- 129, 73  (4g) | AF_SOL-514 |
| unc-22A RVL-2 | 3 min | MiSeq | unc-22A | 0.2 | ~10ng | AF-KLA- 129, 74  (4h) | AF_SOL-514 |
| unc-22A RVL-2 | 12 min | MiSeq | unc-22A | 0.2 | ~10ng | AF-KLA-130, 67  (5a) | AF_SOL-514 |
| unc-22A RVL-2 | 12 min | MiSeq | unc-22A | 0.2 | ~10ng | AF-KLA-130, 68  (5b) | AF_SOL-514 |
| unc-22A RVL-2 | 12 min | MiSeq | unc-22A | 0.2 | ~10ng | AF-KLA-130, 69  (5c) | AF_SOL-514 |
| unc-22A RVL-2 | 12 min | MiSeq | unc-22A | 0.2 | ~10ng | AF-KLA-130, 70  (5d) | AF_SOL-514 |
| unc-22A RVL-2 | 48 min | MiSeq | unc-22A | 0.2 | ~10ng | AF-KLA-130, 71  (5e) | AF_SOL-514 |
| unc-22A RVL-2 | 48 min | MiSeq | unc-22A | 0.2 | ~10ng | AF-KLA-130, 72  (5f) | AF_SOL-514 |
| unc-22A RVL-2 | 48 min | MiSeq | unc-22A | 0.2 | ~10ng | AF-KLA-130, 73  (5g) | AF_SOL-514 |
| unc-22A RVL-2 | 48 min | MiSeq | unc-22A | 0.2 | ~10ng | AF-KLA-130, 74  (5h) | AF_SOL-514 |
| unc-22A RVL-2 | 48 min | MiSeq | unc-22A | 0.2 | ~10ng | AF-KLA-131, 67  (6a) | AF_SOL-514 |
| unc-22A RVL-2 | 48 min | MiSeq | unc-22A | 0.2 | ~10ng | AF-KLA-131, 68  (6b) | AF_SOL-514 |
| unc-22A RVL-2 | 12 min | MiSeq | unc-22A | 0.2 | ~10ng | AF-KLA-131, 69  (6c) | AF_SOL-514 |
| unc-22A RVL-2 | 12 min | MiSeq | unc-22A | 0.2 | ~10ng | AF-KLA-131, 70  (6d) | AF_SOL-514 |
| unc-22A RVL- 1 | N/A | MiSeq | unc-22A | 0 | ~10ng | AF-KLA-128, 67 (5a) | AF_SOL_515 |
| unc-22A RVL- 1 | N/A | MiSeq | unc-22A | 0 | ~10ng | AF-KLA-128, 68 (5b) | AF_SOL_515 |
| unc-22A RVL-2 | N/A | MiSeq | unc-22A | 0 | ~10ng | AF-KLA-128, 69 (5c) | AF_SOL_515 |
| unc-22A RVL- 1 | N/A | MiSeq | unc-22A | 0 | ~10ng | AF-KLA-128, 70 (5d) | AF_SOL_515 |
| unc-22A RVL- 1 | 0 | MiSeq | unc-22A | 0.2 | ~10ng | AF-KLA-128, 73 (5g) | AF_SOL_515 |
| unc-22A RVL- 1 | 0 | MiSeq | unc-22A | 0.2 | ~10ng | AF-KLA-128, 74 (5h) | AF_SOL_515 |
| unc-22A RVL- 1 | 1 min | MiSeq | unc-22A | 0.2 | ~10ng | AF-KLA-129, 69  (6c) | AF_SOL_515 |
| unc-22A RVL- 1 | 1 min | MiSeq | unc-22A | 0.2 | ~10ng | AF-KLA-129, 70  (6d) | AF_SOL_515 |
| unc-22A RVL- 1 | 3 min | MiSeq | unc-22A | 0.2 | ~10ng | AF-KLA-130, 71  (7e) | AF_SOL_515 |
| unc-22A RVL- 1 | 3 min | MiSeq | unc-22A | 0.2 | ~10ng | AF-KLA-130, 72  (7f) | AF_SOL_515 |
| unc-22A RVL- 1 | 3 min | MiSeq | unc-22A | 0.2 | ~10ng | AF-KLA-130, 73  (7g) | AF_SOL_515 |
| unc-22A RVL- 1 | 3 min | MiSeq | unc-22A | 0.2 | ~10ng | AF-KLA-130, 74  (7h) | AF_SOL_515 |
| unc-22A RVL- 1 | 12 min | MiSeq | unc-22A | 0.2 | ~10ng | AF-KLA-131, 67  (8a) | AF_SOL_515 |
| unc-22A RVL- 1 | 12 min | MiSeq | unc-22A | 0.2 | ~10ng | AF-KLA-131, 68  (8b) | AF_SOL_515 |
| unc-22A RVL- 1 | 12 min | MiSeq | unc-22A | 0.2 | ~10ng | AF-KLA-131, 69  (8c) | AF_SOL_515 |
| unc-22A RVL- 1 | 12 min | MiSeq | unc-22A | 0.2 | ~10ng | AF-KLA-131, 70  (8d) | AF_SOL_515 |
| unc-22A RVL- 1 | 48 min | MiSeq | unc-22A | 0.2 | ~10ng | AF-KLA-131, 71  (8e) | AF_SOL_515 |
| unc-22A RVL- 1 | 48 min | MiSeq | unc-22A | 0.2 | ~10ng | AF-KLA-131, 72  (8f) | AF_SOL_515 |
| unc-22A RVL- 1 | 48 min | MiSeq | unc-22A | 0.2 | ~10ng | AF-KLA-132, 67  (9a) | AF_SOL_515 |
| unc-22A RVL- 1 | 48 min | MiSeq | unc-22A | 0.2 | ~10ng | AF-KLA-132, 68  (9b) | AF_SOL_515 |
| unc-22A RVL- 1 | 48 min | MiSeq | unc-22A | 0.2 | ~10ng | AF-KLA-132, 71  (9e) | AF_SOL_515 |
| unc-22A RVL- 1 | 48 min | MiSeq | unc-22A | 0.2 | ~10ng | AF-KLA-132, 72  (9f) | AF_SOL_515 |
| unc-22A RVL- 1 | 48 min | MiSeq | unc-22A | 0.2 | ~10ng | AF-KLA-132, 73  (9g) | AF_SOL_515 |
| unc-22A RVL- 1 | 48 min | MiSeq | unc-22A | 0.2 | ~10ng | AF-KLA-132, 74  (9h) | AF_SOL_515 |
| unc-22A RVL- 1 | 3 min | MiSeq | unc-22A | 0.2 | ~10 ng | AF-KLA-124, 67  (1a) | AF_SOL_516 |
| unc-22A RVL- 1 | 3 min | MiSeq | unc-22A | 0.2 | ~10 ng | AF-KLA-124, 68  (1b) | AF_SOL_516 |
| unc-22A RVL- 1 | 3 min | MiSeq | unc-22A | 0.2 | ~10 ng | AF-KLA-124, 69  (1c) | AF_SOL_516 |
| unc-22A RVL- 1 | 3 min | MiSeq | unc-22A | 0.2 | ~10 ng | AF-KLA-124, 70  (1d) | AF_SOL_516 |
| unc-22A RVL- 1 | 3 min | MiSeq | unc-22A | 0.2 | ~10 ng | AF-KLA-124, 71  (1e) | AF_SOL_516 |
| unc-22A RVL- 1 | 3 min | MiSeq | unc-22A | 0.2 | ~10 ng | AF-KLA-124, 72  (1f) | AF_SOL_516 |
| unc-22A RVL- 1 | 3 min | MiSeq | unc-22A | 0.2 | ~40 ng | AF-KLA-124, 73  (1g) | AF_SOL_516 |
| unc-22A RVL- 1 | 3 min | MiSeq | unc-22A | 0.2 | ~40 ng | AF-KLA-124, 74  (1h) | AF_SOL_516 |
| unc-22A RVL- 1 | 3 min | MiSeq | unc-22A | 0.2 | ~40 ng | AF-KLA-126, 67  (2a) | AF_SOL_516 |
| unc-22A RVL- 1 | 3 min | MiSeq | unc-22A | 0.2 | ~40 ng | AF-KLA-126, 68  (2b) | AF_SOL_516 |
| unc-22A RVL- 1 | 3 min | MiSeq | unc-22A | 0.2 | ~40 ng | AF-KLA-126, 69  (2c) | AF_SOL_516 |
| unc-22A RVL- 1 | 3 min | MiSeq | unc-22A | 0.2 | ~40 ng | AF-KLA-126, 70  (2d) | AF_SOL_516 |
| unc-22A RVL- 1 | 180 min | MiSeq | unc-22A | 0.2 | ~10 ng | AF-KLA-126, 71  (2e) | AF_SOL_516 |
| unc-22A RVL- 1 | 180 min | MiSeq | unc-22A | 0.2 | ~10 ng | AF-KLA-126, 72  (2f) | AF_SOL_516 |
| unc-22A RVL- 1 | 180 min | MiSeq | unc-22A | 0.2 | ~10 ng | AF-KLA-126, 73  (2g) | AF_SOL_516 |
| unc-22A RVL- 1 | 180 min | MiSeq | unc-22A | 0.2 | ~10 ng | A181F-KLA-126, 74  (2h) | AF_SOL_516 |
| unc-22A RVL- 1 | 180 min | MiSeq | unc-22A | 0.2 | ~10 ng | AF-KLA-127, 67  (3a) | AF_SOL_516 |
| unc-22A RVL- 1 | 180 min | MiSeq | unc-22A | 0.2 | ~10 ng | AF-KLA-127, 68  (3b) | AF_SOL_516 |
| unc-22A RVL- 1 | 360 min | MiSeq | unc-22A | 0.2 | ~10 ng | AF-KLA-129, 67  (4a) | AF_SOL_516 |
| unc-22A RVL- 1 | 360 min | MiSeq | unc-22A | 0.2 | ~10 ng | AF-KLA-129, 68  (4b) | AF_SOL_516 |
| unc-22A RVL- 1 | 360 min | MiSeq | unc-22A | 0.2 | ~10 ng | AF-KLA-129, 69  (4c) | AF_SOL_516 |
| unc-22A RVL- 1 | 360 min | MiSeq | unc-22A | 0.2 | ~10 ng | AF-KLA-129, 70  (4d) | AF_SOL_516 |
| unc-22A RVL- 1 | 360 min | MiSeq | unc-22A | 0.2 | ~10 ng | AF-KLA-129, 71  (4e) | AF_SOL_516 |
| unc-22A RVL- 1 | 360 min | MiSeq | unc-22A | 0.2 | ~10 ng | AF-KLA-129, 72  (4f) | AF_SOL_516 |
| unc-22A RVL- 1 | 180 min | MiSeq | unc-22A | 0.2 | ~40 ng | AF-KLA-129, 73  (4g) | AF_SOL_516 |
| unc-22A RVL- 1 | 180 min | MiSeq | unc-22A | 0.2 | ~40 ng | AF-KLA-129, 74  (4h) | AF_SOL_516 |
| unc-22A RVL- 1 | 180 min | MiSeq | unc-22A | 0.2 | ~40 ng | AF-KLA-130, 67  (5a) | AF_SOL_516 |
| unc-22A RVL- 1 | 180 min | MiSeq | unc-22A | 0.2 | ~40 ng | AF-KLA-130, 68  (5b) | AF_SOL_516 |
| unc-22A RVL- 1 | 180 min | MiSeq | unc-22A | 0.2 | ~40 ng | AF-KLA-130, 69  (5c) | AF_SOL_516 |
| unc-22A RVL- 1 | 180 min | MiSeq | unc-22A | 0.2 | ~40 ng | AF-KLA-130, 70  (5d) | AF_SOL_516 |
| unc-22A RVL- 1 | N/A | MiSeq | unc-22A | 0 | ~40 ng | AF-KLA-132, 71  (7e) | AF_SOL_516 |
| unc-22A RVL- 1 | N/A | MiSeq | unc-22A | 0 | ~40 ng | AF-KLA-132, 72  (7f) | AF_SOL_516 |
| unc-22A RVL- 1 | N/A | MiSeq | unc-22A | 0 | ~40 ng | AF-KLA-132, 73  (7g) | AF_SOL_516 |
| unc-22A RVL- 1 | N/A | MiSeq | unc-22A | 0 | ~40 ng | AF-KLA-132, 74  (7h) | AF_SOL_516 |
| unc-22A RVL- 1 | N/A | MiSeq | unc-22A | 0 | ~10 ng | AF-KLA-125, 67  (9a) | AF_SOL_516 |
| unc-22A RVL- 1 | N/A | MiSeq | unc-22A | 0 | ~10 ng | AF-KLA-125, 68  (9b) | AF_SOL_516 |
| unc-22A RVL- 1 | N/A | MiSeq | unc-22A | 0 | ~10 ng | AF-KLA-125, 69  (9c) | AF_SOL_516 |
| unc-22A RVL- 1 | N/A | MiSeq | unc-22A | 0 | ~10 ng | AF-KLA-125, 70  (9d) | AF_SOL_516 |

Each line in the table refers to a single cleavage reaction that was amplified and subject to Illumina sequencing. Input libraries were from the unc-22A region or ps4 as noted. Two separately prepared libraries for unc-22A were used and are designated: "RVL-2" and "RVL 1". Libraries were amplified through two consecutive rounds of PCR. Amplification PCR conditions were as follows: PCR 1 mix consisted of 2ul of extracted DNA, 0.35 ul of AF-KLA-136 (at 100 pmoles/ul), 0.35 ul of AF-KLA-140 (at 100 pmoles/ul), 17.5 ul 2X Phusion Master Mix buffer, 1 ul of DMSO, and 13.8 ul of dH20. Input library material was amplified with the following PCR conditions: 98 ° C for 1 min, (98° C for 10 secs, 52° C for 20 secs, 72° C for 30 secs) x 2 cycles , (98° C for 10 secs, 57° C for 20 secs, 72° C for 30 secs) x 18 cycles , 72° C for 10 mins, and 10° C infinity.

Following the first amplification, a second round of amplification was performed to add sequencing primers. PCR 2 mix consisted of: 4 ul of PCR 1, 0.35 ul forward long primer (at 100 pmoles/ul), 0.35 ul of reverse long primer (at 100 pmoles/ul), 17.5 ul 2X Master Mix Phusion buffer, 1 ul of DMSO, and 11.8 ul dH20. The second amplification was done with the following PCR conditions: 98 ° C for 1 min, (98° C for 10 secs, 54° C for 20 secs, 72° C for 30 secs) x 2 cycles, (98° C for 10 secs, 70° C for 20 secs, 72° C for 30 secs) x 4 cycles, 72° C for 10 mins, and 10° C infinity. All 35 ul of PCR 2 is run on a 3% NuSieve agarose gel. The bands are cut out and purified using the Qiagen Gel Extraction kit. **Figure S1:** Additional Cas9 *in vitro* experiments on unc-22A.

a) A replicate of the experiment in Figure 2 with an alternative unc-22A library RVL-2 (10ng) (AF_SOL_514)


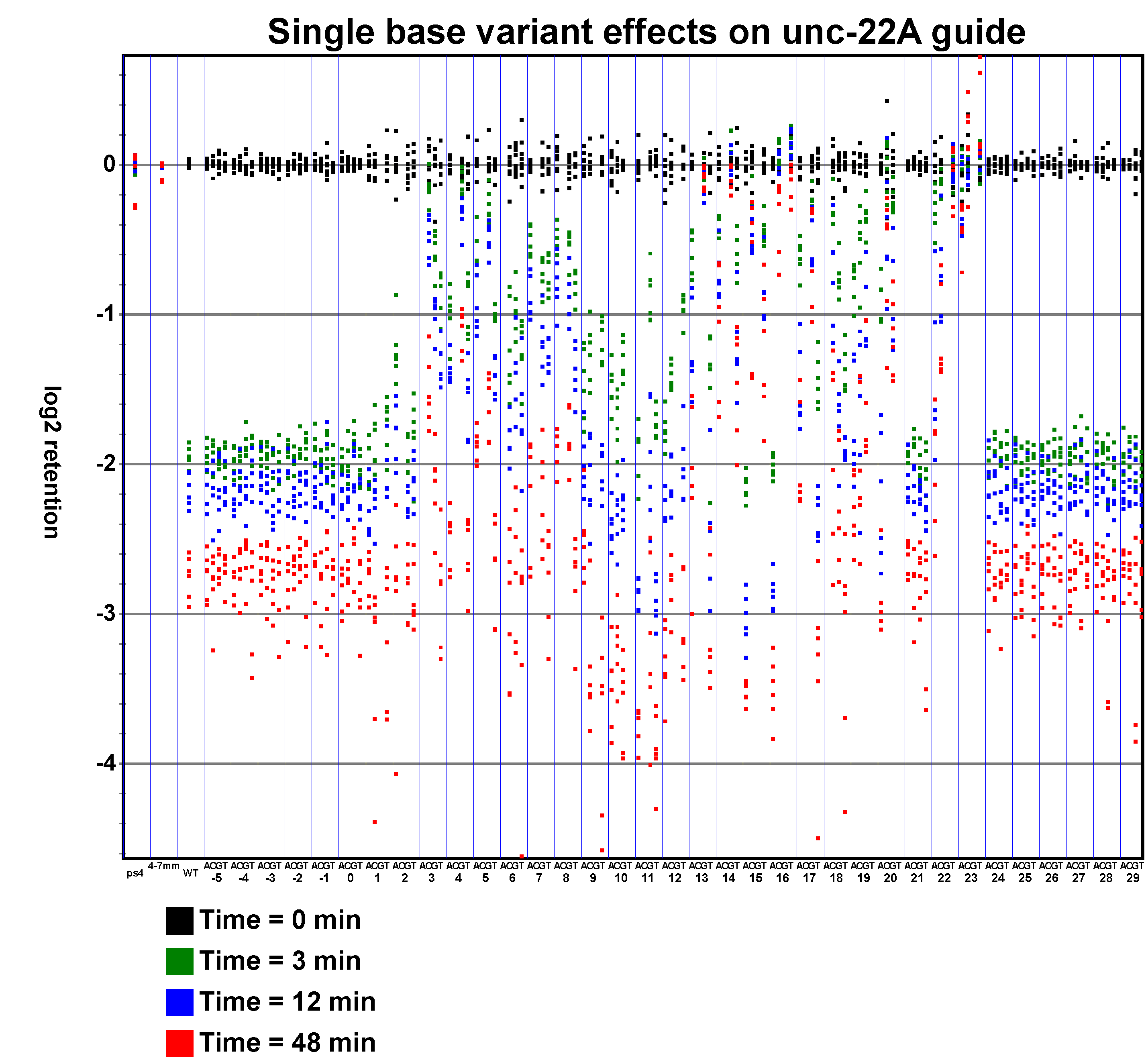


b) An experiment equivalent to Figure 2 with 40ng of library unc-22A RVL-1. (AF_SOL_516)


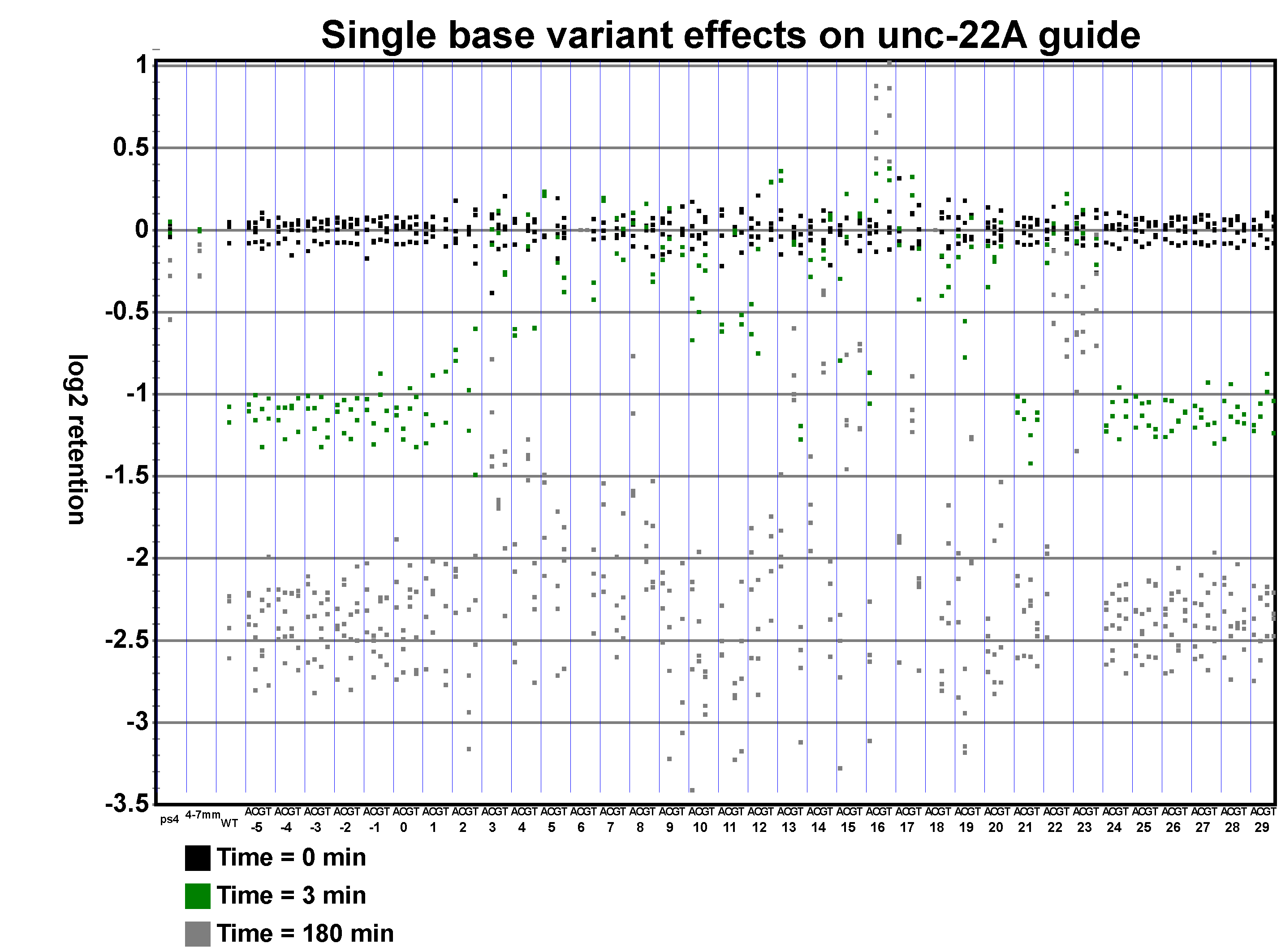


c) An experiment equivalent to Figure 2 using 10ng of unc-22A library RVL-1. (AF_SOL_516)


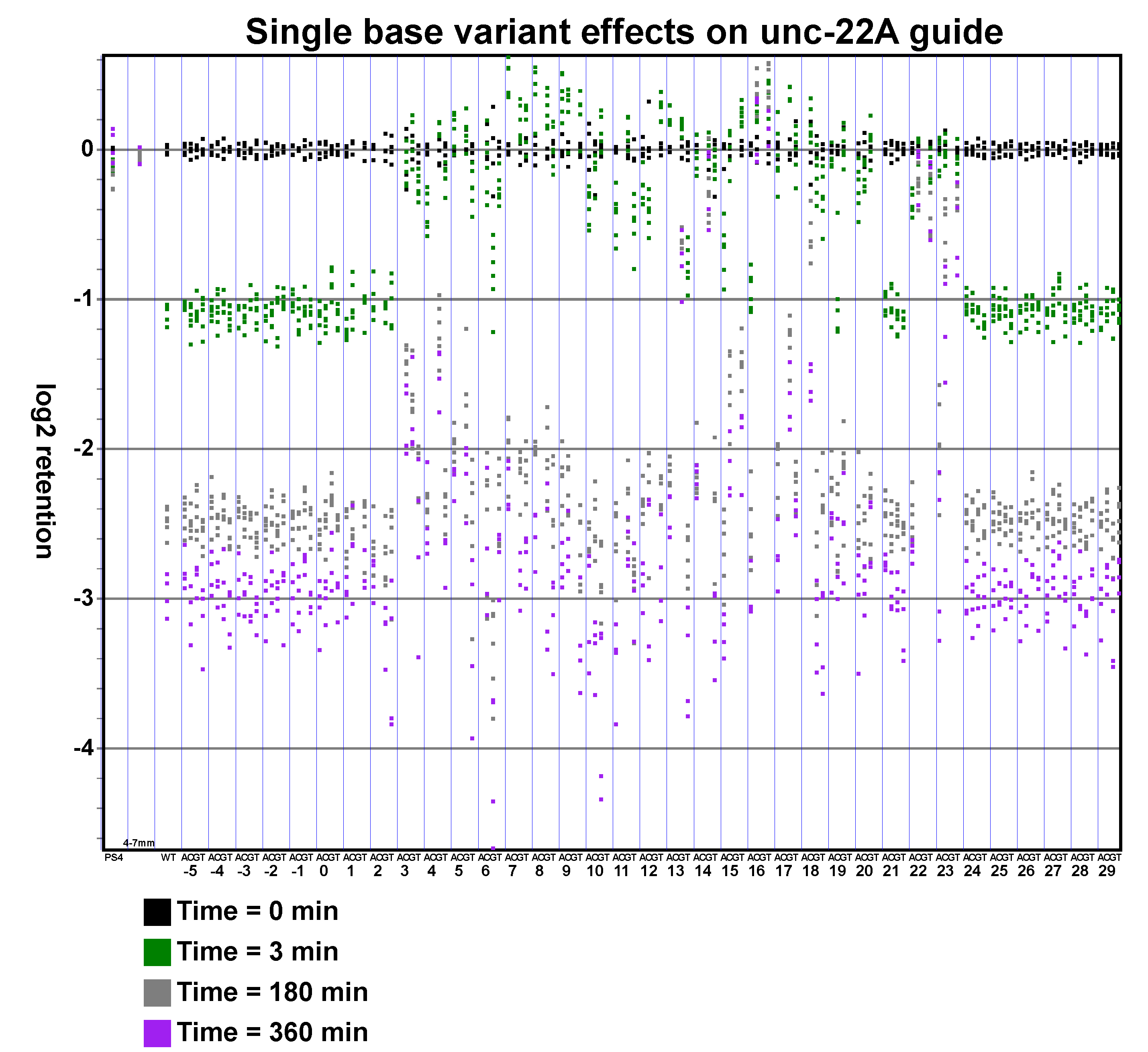


**Figure S2:** Single variant heat maps for additional assays with unc-22A target. The heat map shows a color for each mutation that reflects the ratio (median variant retention)/(median WT retention) as shown in the Color Key. Boxes with asterisks indicate the WT base. Values with the score of ~1 (white) are variants with retentions that do not greatly deviate from the median for all perfectly matched (guide+PAM) targets. Variants with values that are above 1 (blue) correspond to variants that cut more efficiently than WT. Variants with values around ~0 (red) correspond to variants that either do not cleave or cleave very inefficiently.

a) A replicate of the experiment in Figure 3 with an alternative unc-22A library, RVL-2 derived from the same original oligonucleotide pool. (AF_SOL_514)


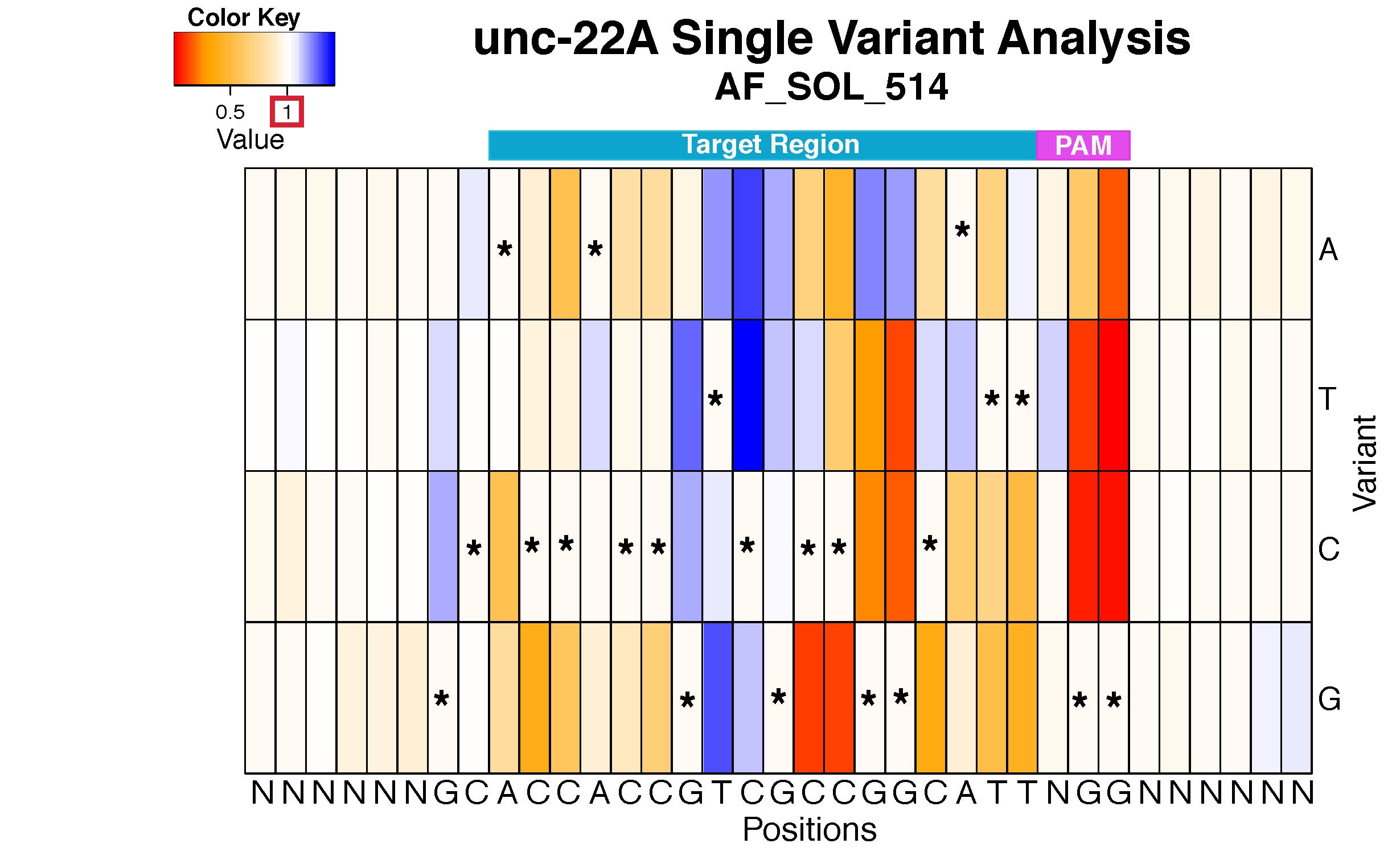


b) An experiment equivalent to Figure 3 with 10ng of library unc-22A RVL-1. (AF_SOL_516)
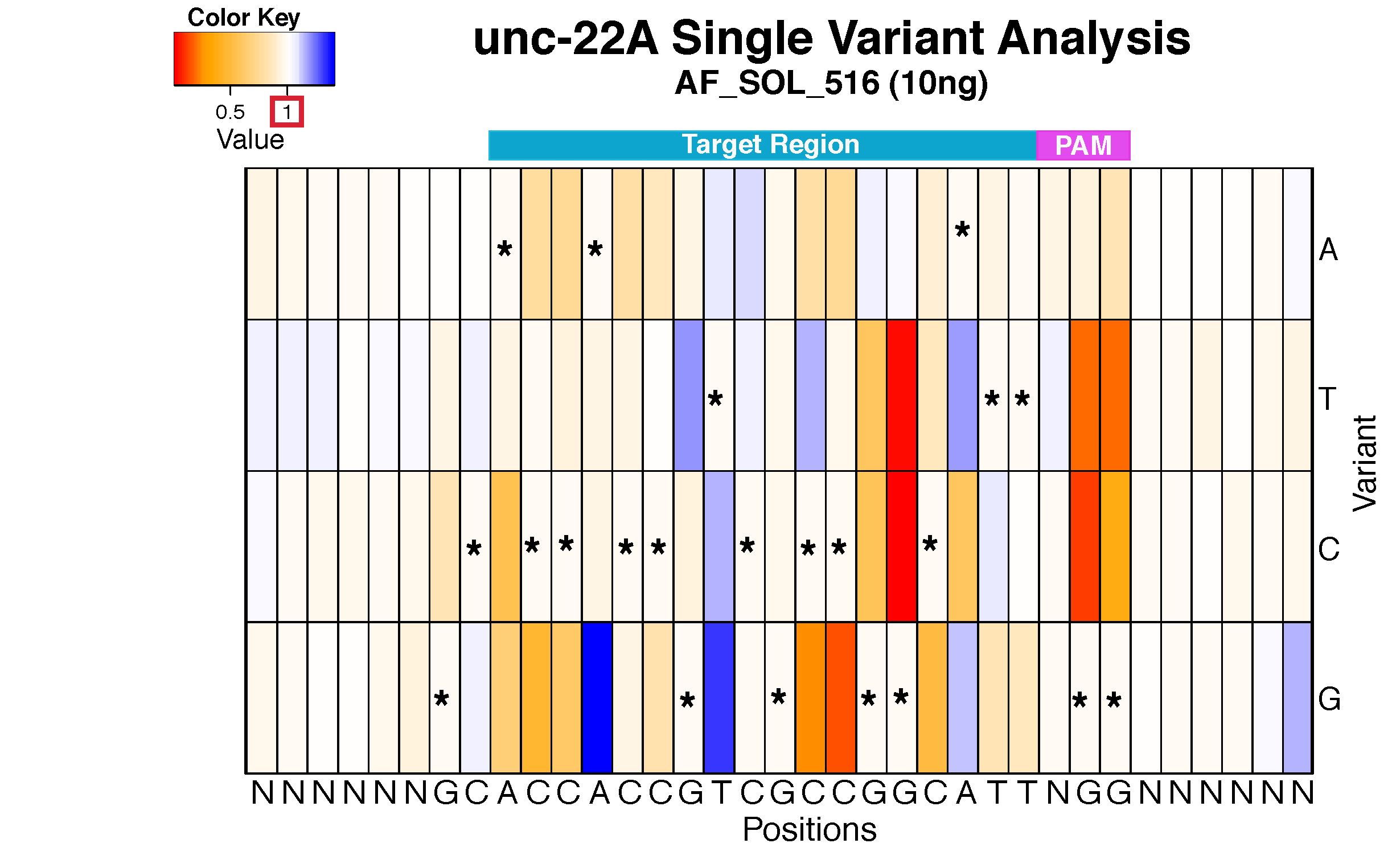


c) An experiment equivalent to Figure 3 with 40ng of library unc-22A RVL-1. (AF_SOL_516)


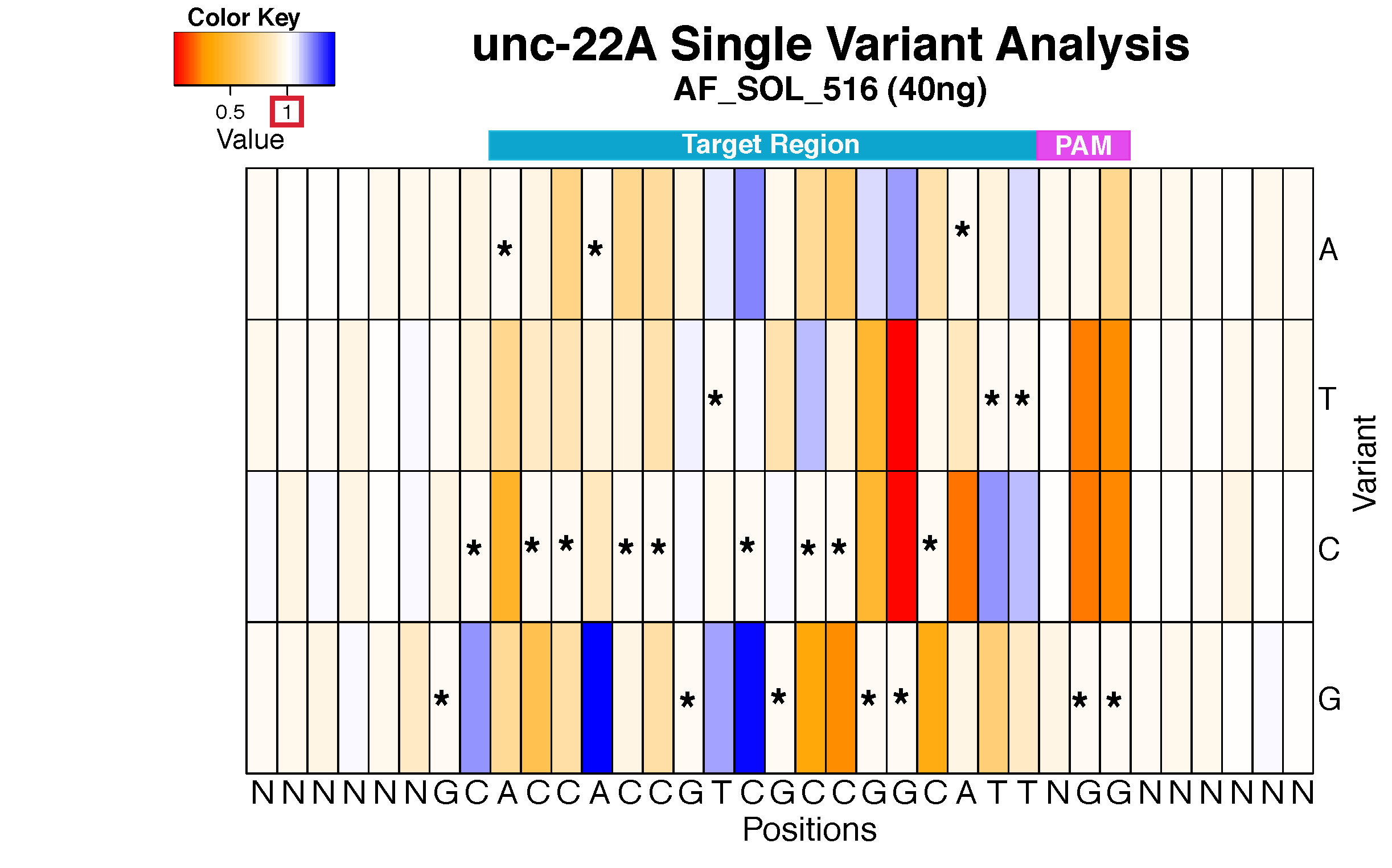


**Figure S3**: Graphs of median retention vs. time for additional experiments.

a) A time-versus-retention plot equivalent to Figure 4 but with an alternative unc-22A library (RVL-2) derived from the same original oligonucleotide pool. (AF_SOL_514)


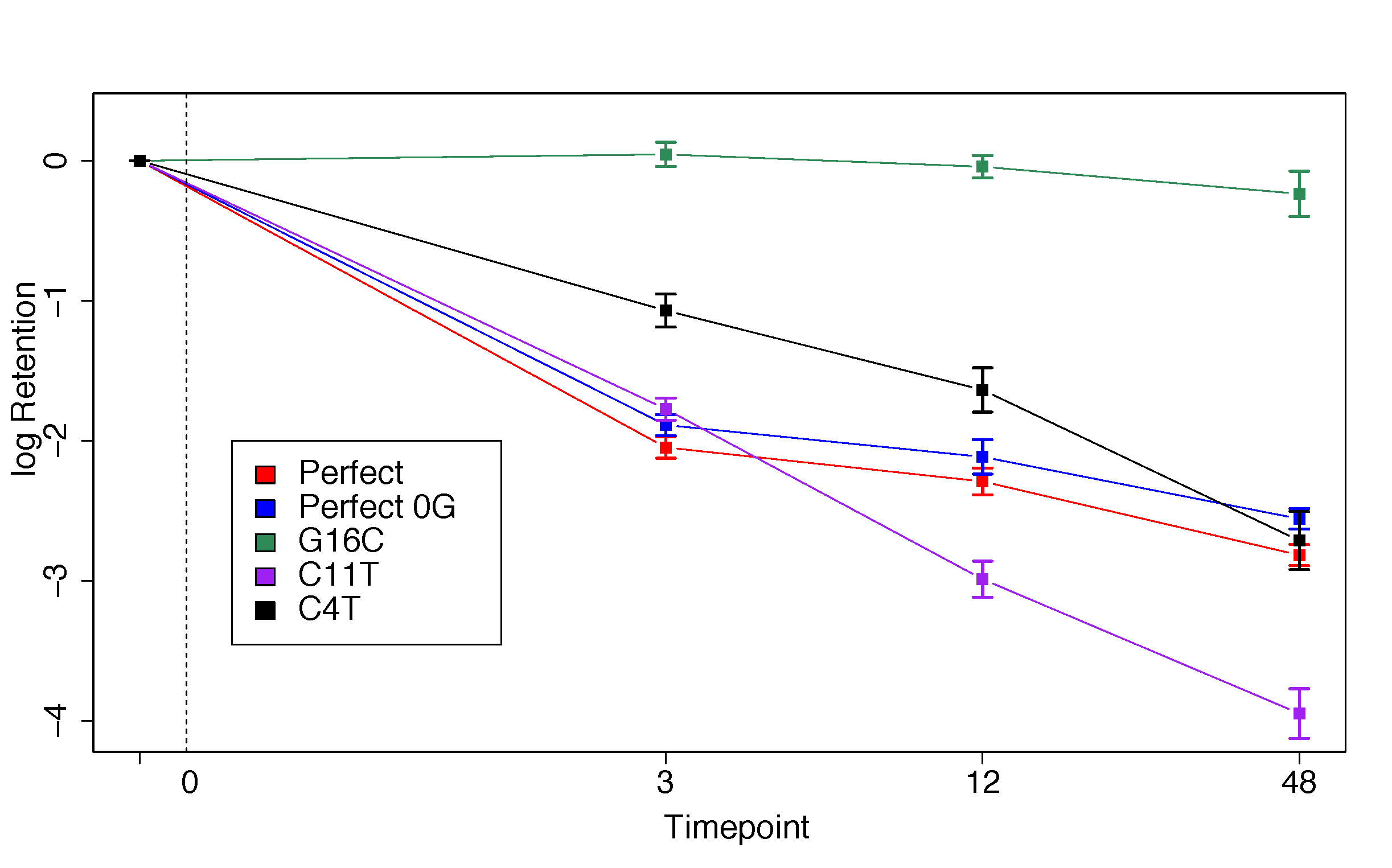


b) An experiment equivalent to Figure 4 with 10ng of library unc-22A RVL-1. (AF_SOL_516)


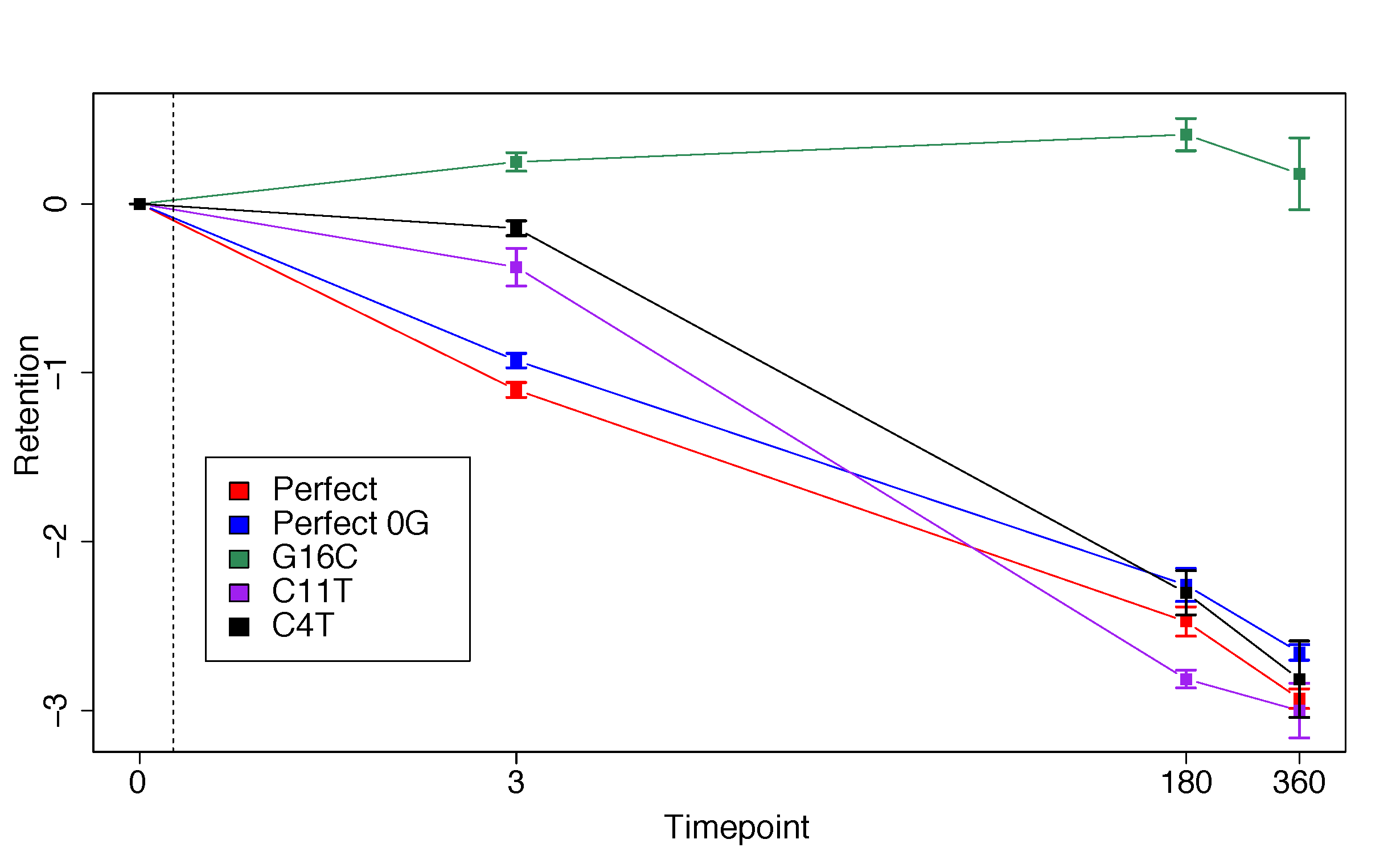


c) An experiment equivalent to Figure 4 with 40ng of library unc-22A RVL-1. (AF_SOL_516)


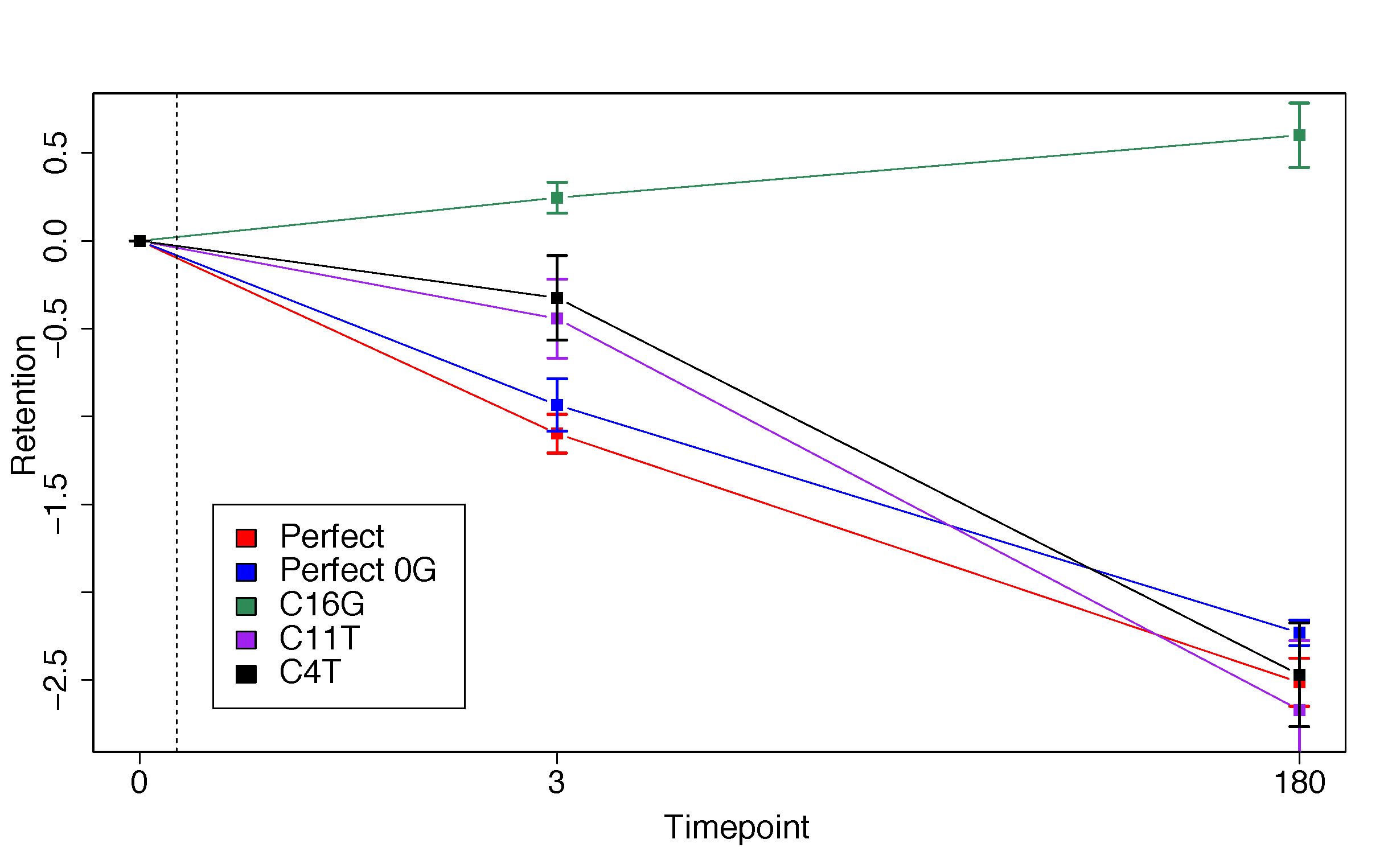


**Figure S4**: Double variant cleavage efficiency visualization from additional experiments. Each box represents the median log retention of the double variant of interest. For reference, the diagonal provides median log retention of the single variants. Red colors correspond to variants that do not cleave efficiently. Blue colors correspond to variants with highly efficient cleavage.

a) A replicate of the experiment in Figure 5a with an alternative unc-22A library, RVL-2 derived from the same original oligonucleotide pool. (AF_SOL_514)


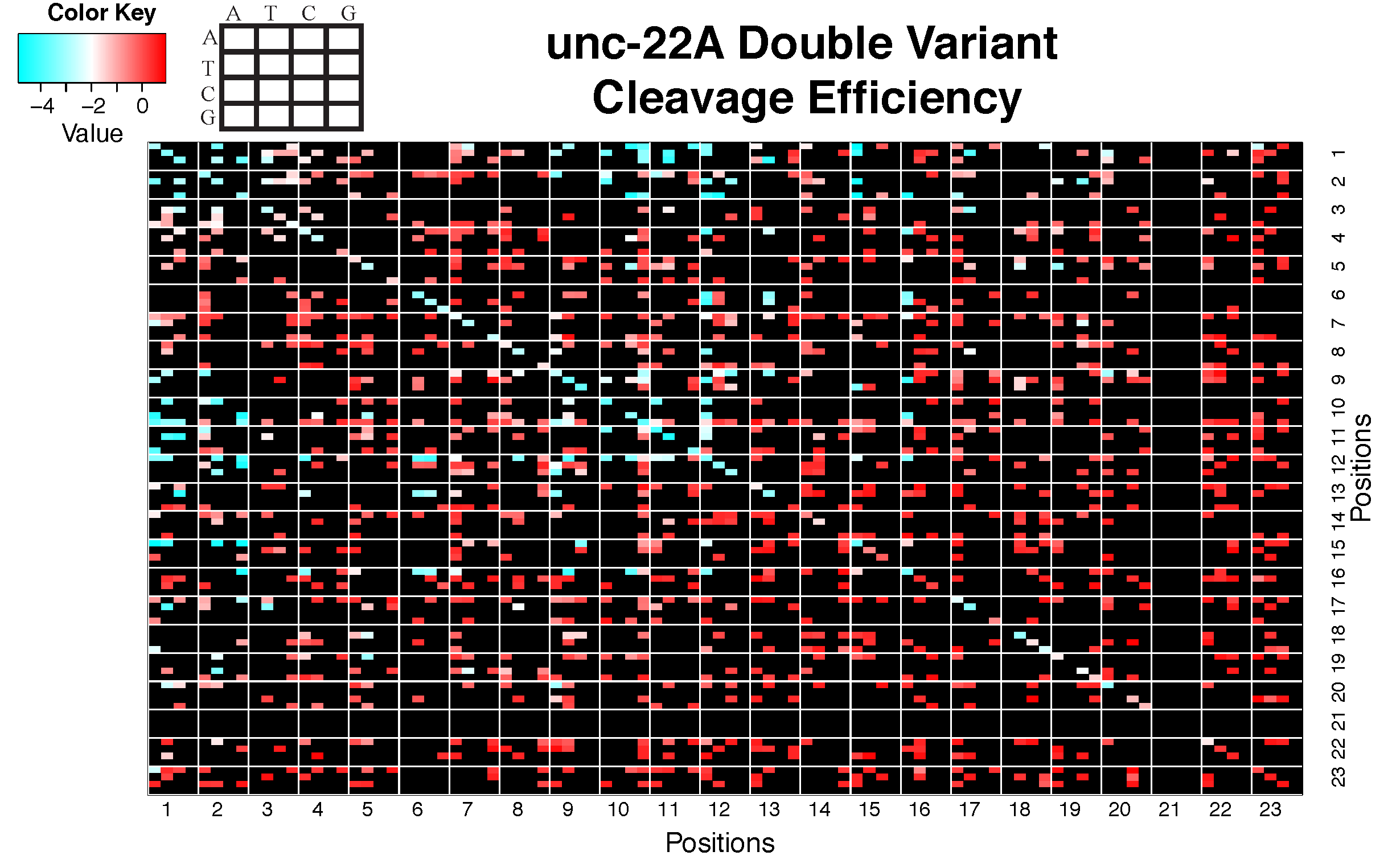


1. An experiment equivalent to Figure 5a with 10ng of library unc-22A RVL-1. (AF_SOL_516)
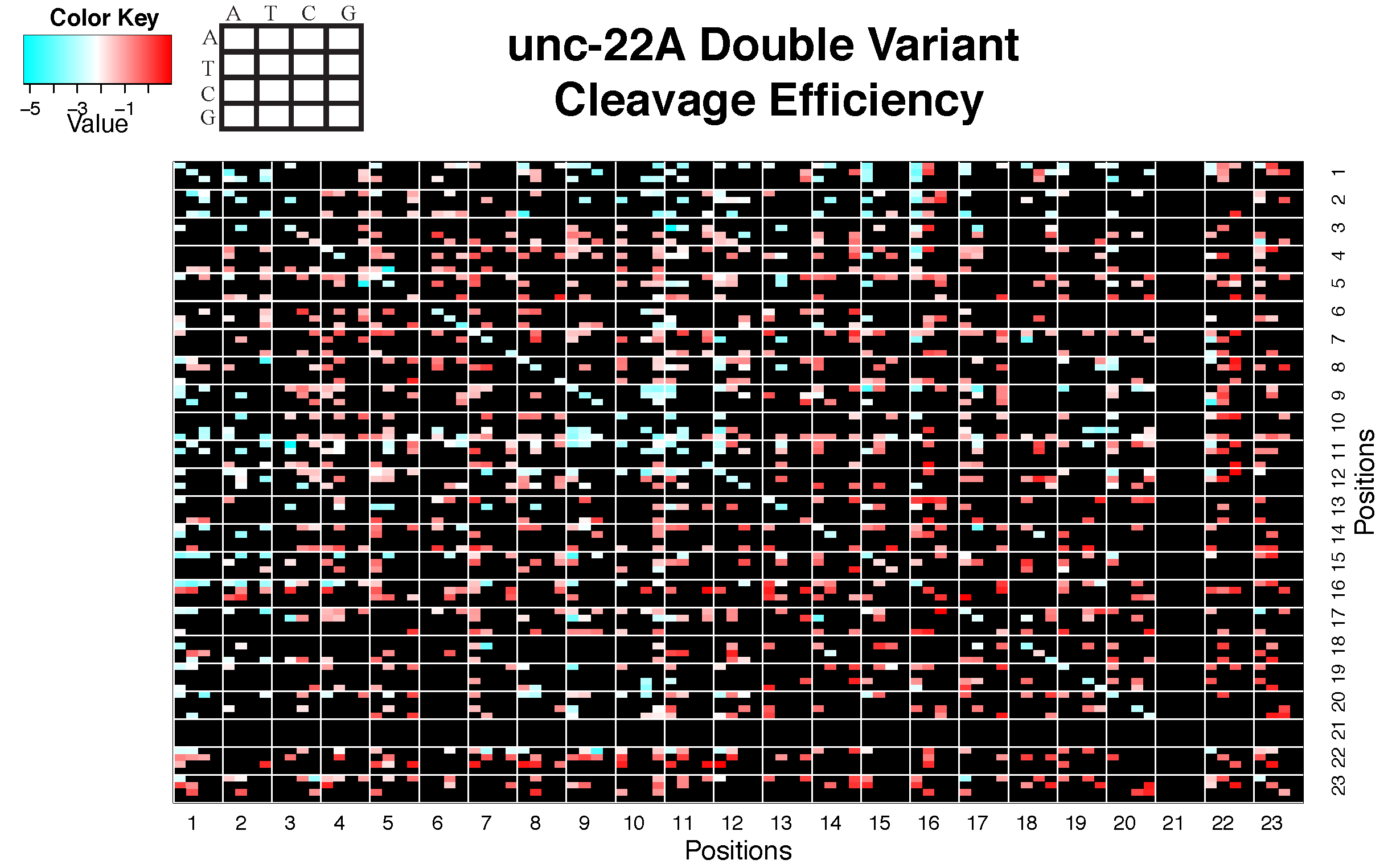


c) An experiment equivalent to Figure 5a with 40ng of library unc-22A RVL-1. (AF_SOL_516)
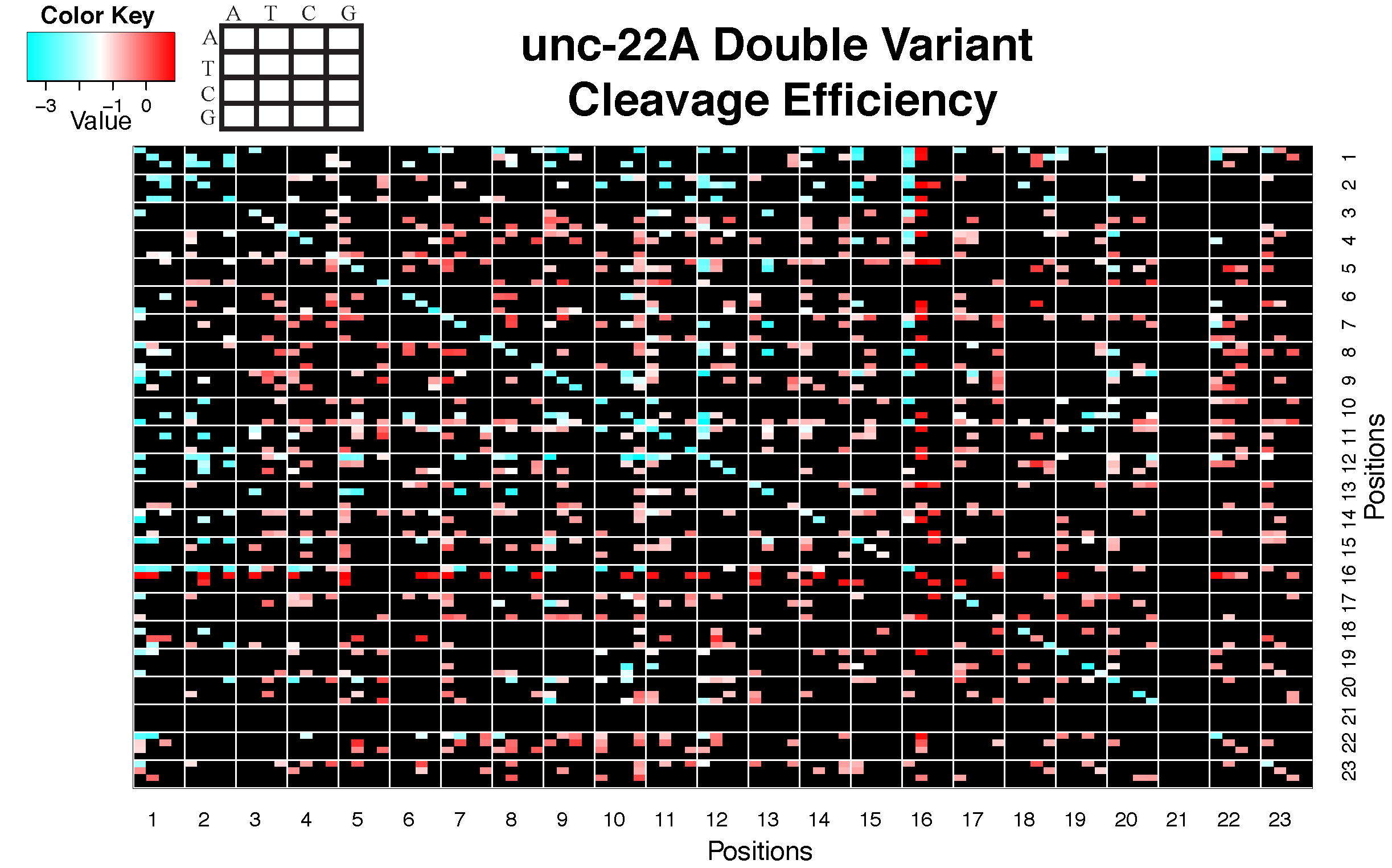


**Figure S5:** Double variant cleavage synergy visualization from additional experiments

a) A replicate of the experiment in Figure 5b with the different unc-22Alibrary (10ng RVL-2 library). (AF_SOL_514)
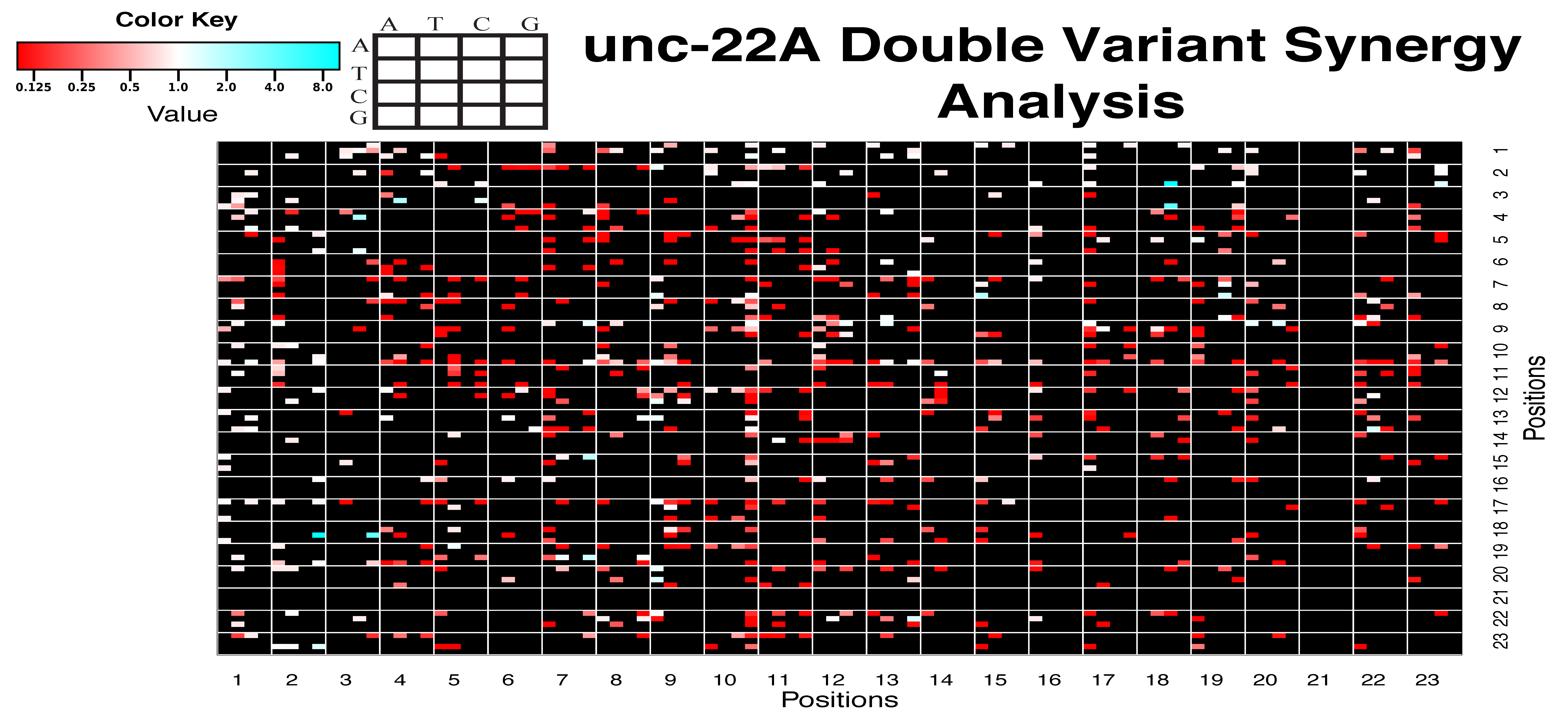


b) An experiment equivalent to Figure 5b, using 40 ng of library RVL-1. (AF_SOL_516)
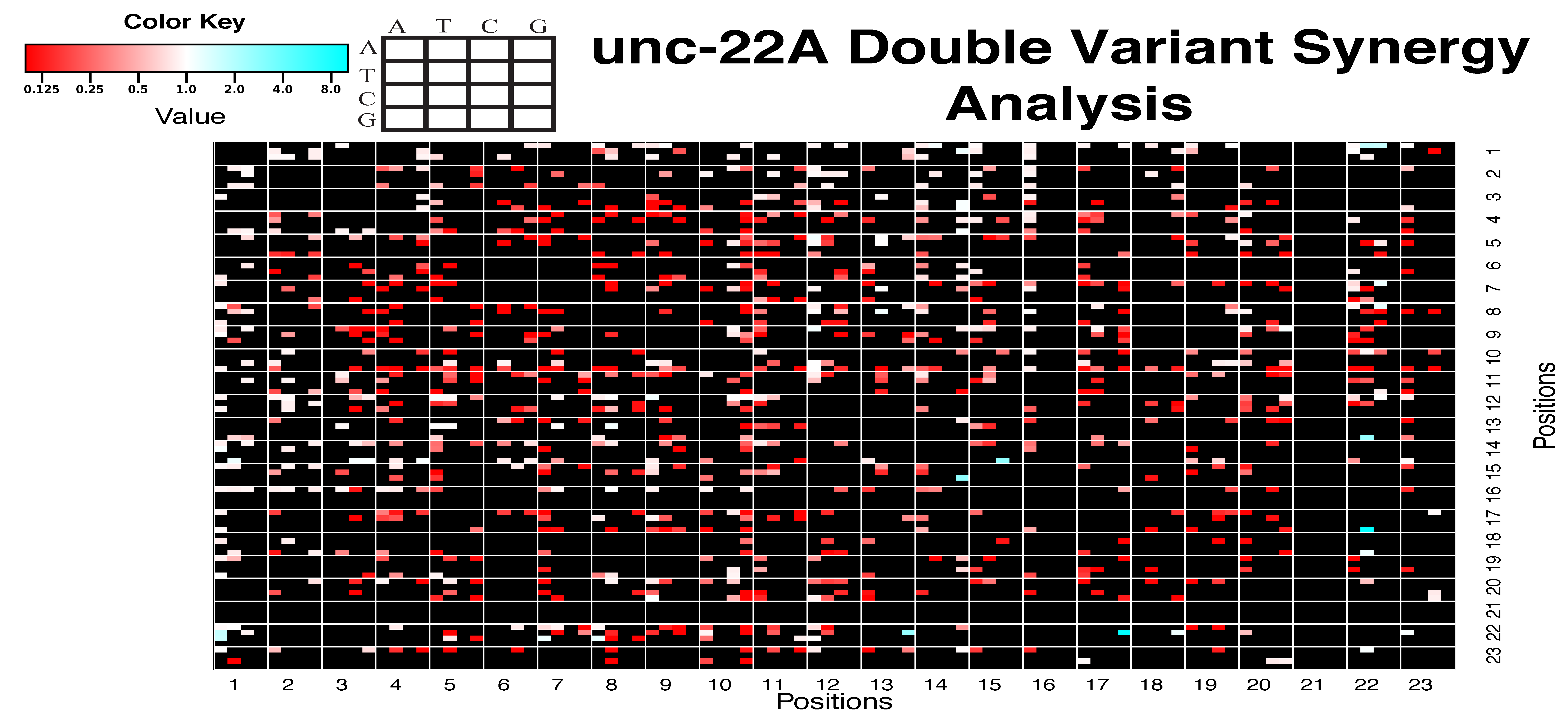


c) An experiment equivalent to Figure 5b, using 10 ng of library RVL-1. (AF_SOL_516)

**
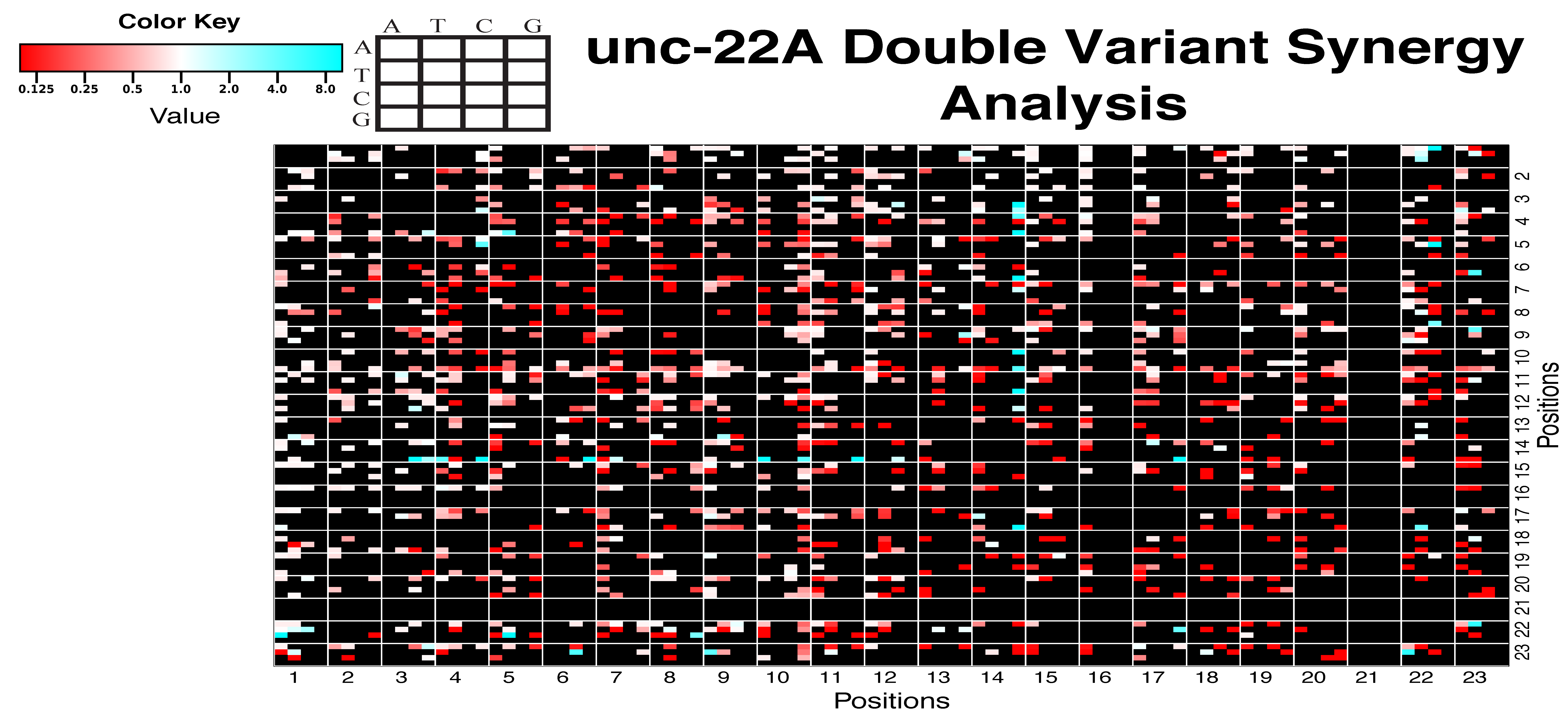
Figure S6**: Consistency of data between individual experimental replicates in a single run. Each experiment had PCR replicates and experimental replicates. These plots show the close correspondence between experimental and technical replicates.

a) Correlation plots for AF_SOL_515 between PCR replicates (unc-22A variant library).


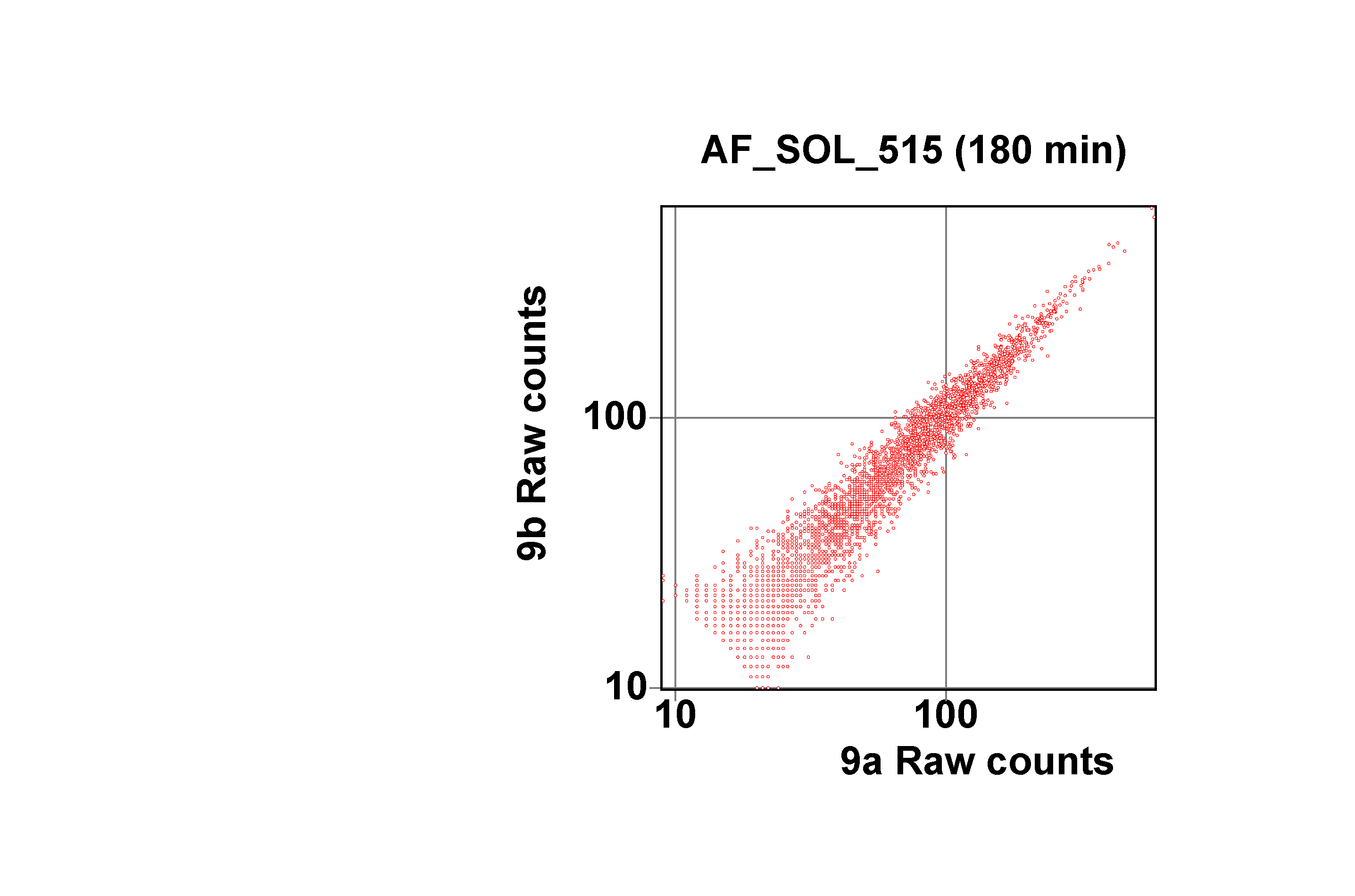

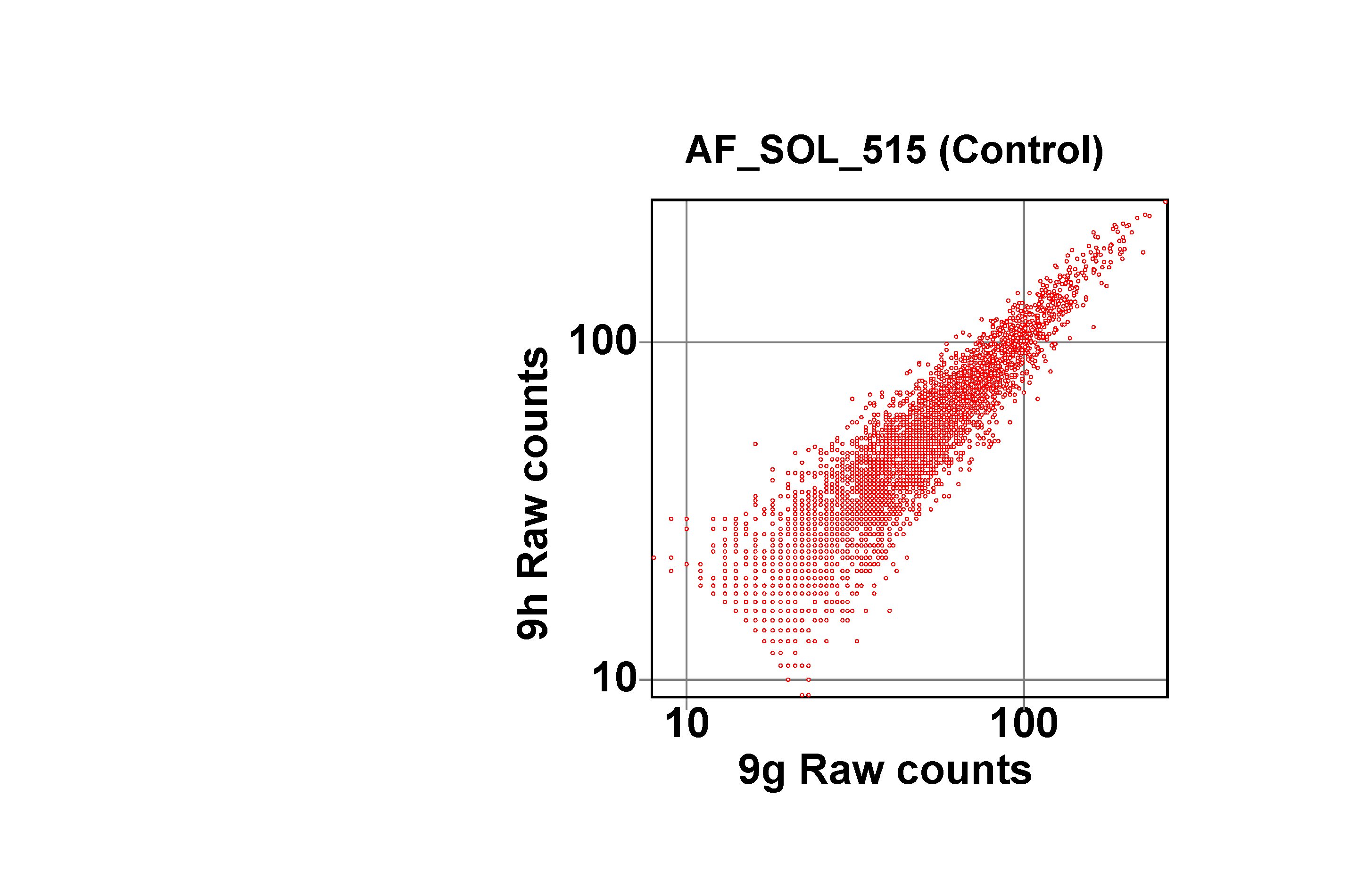

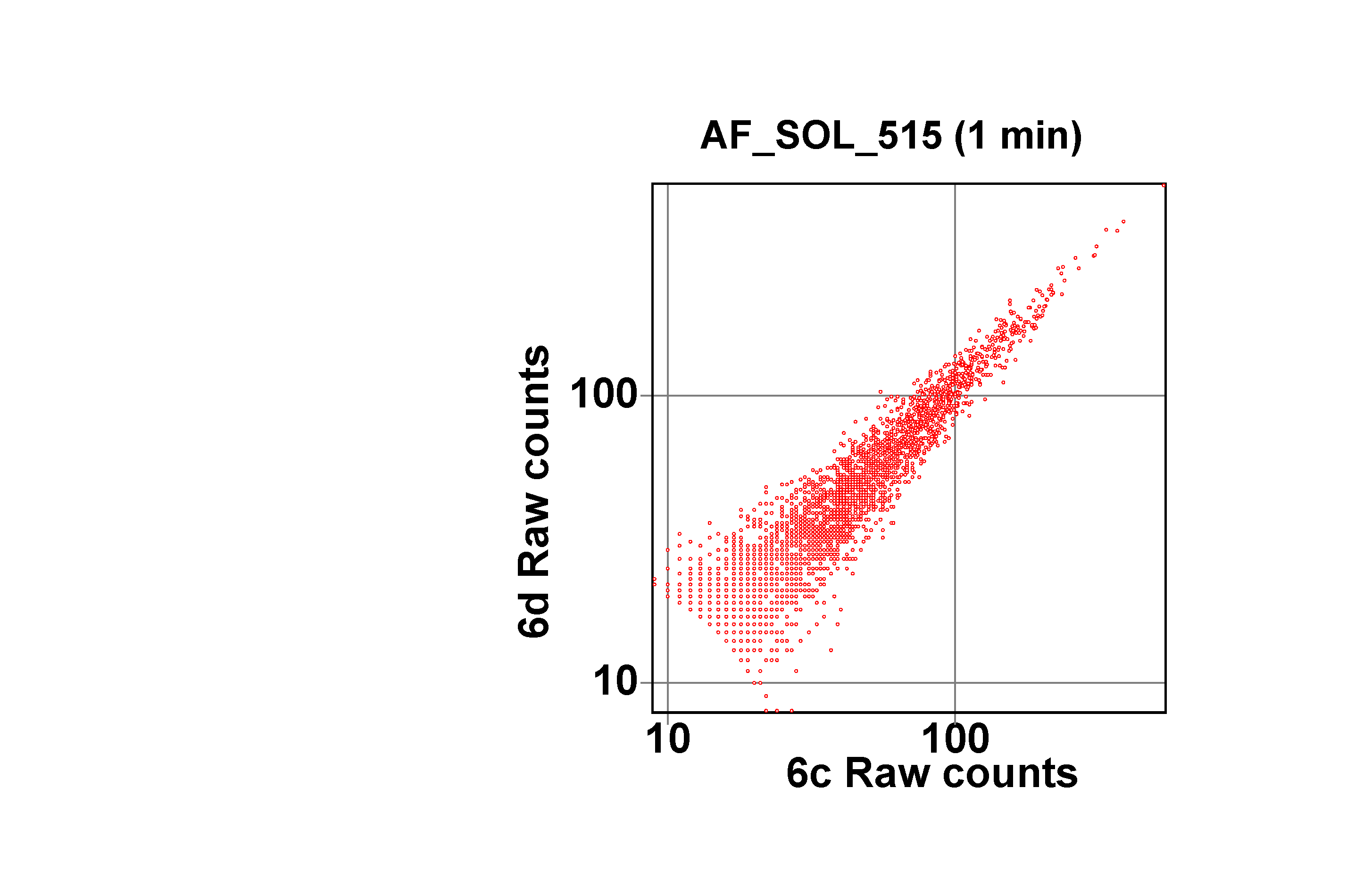

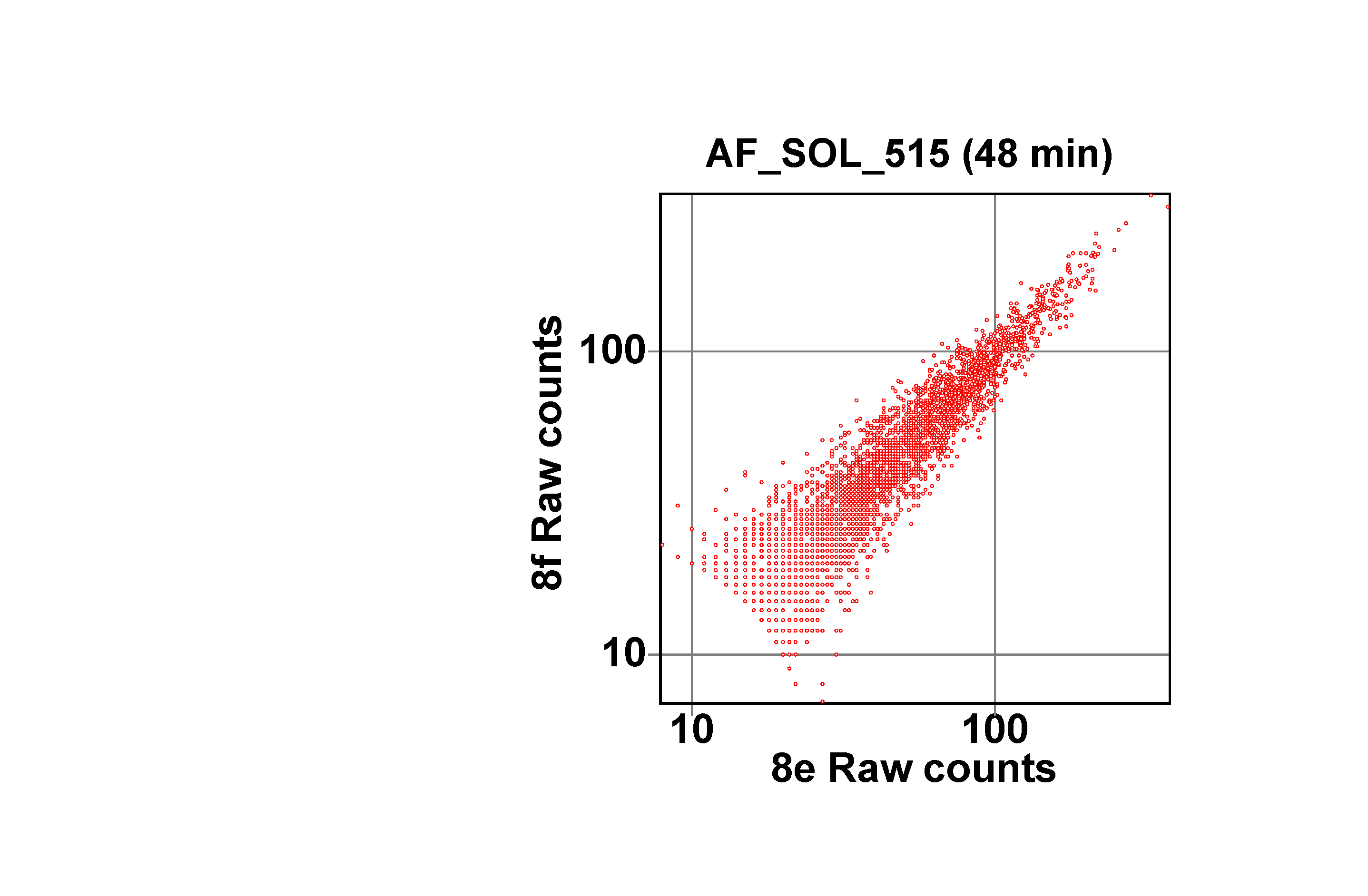

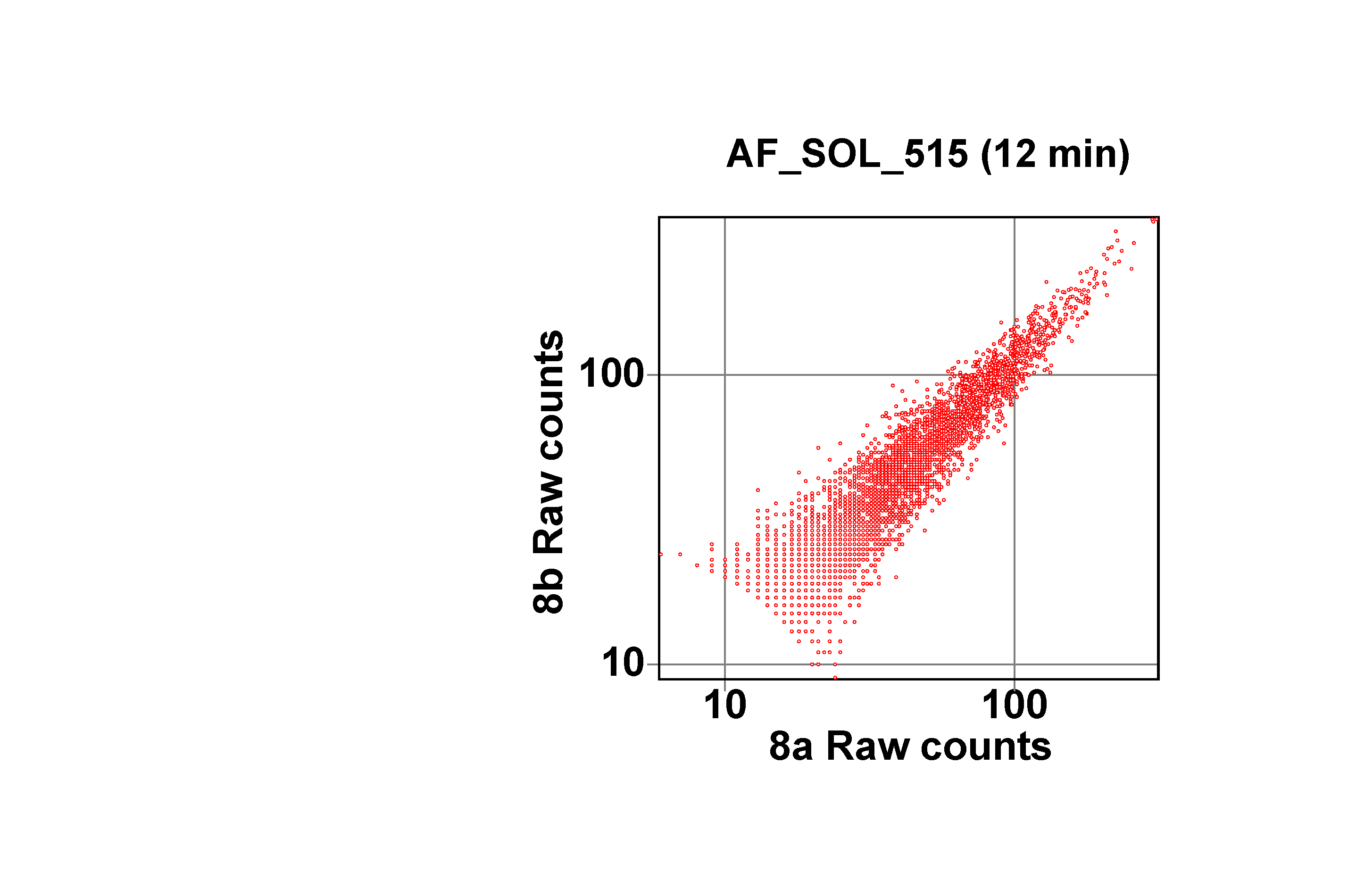

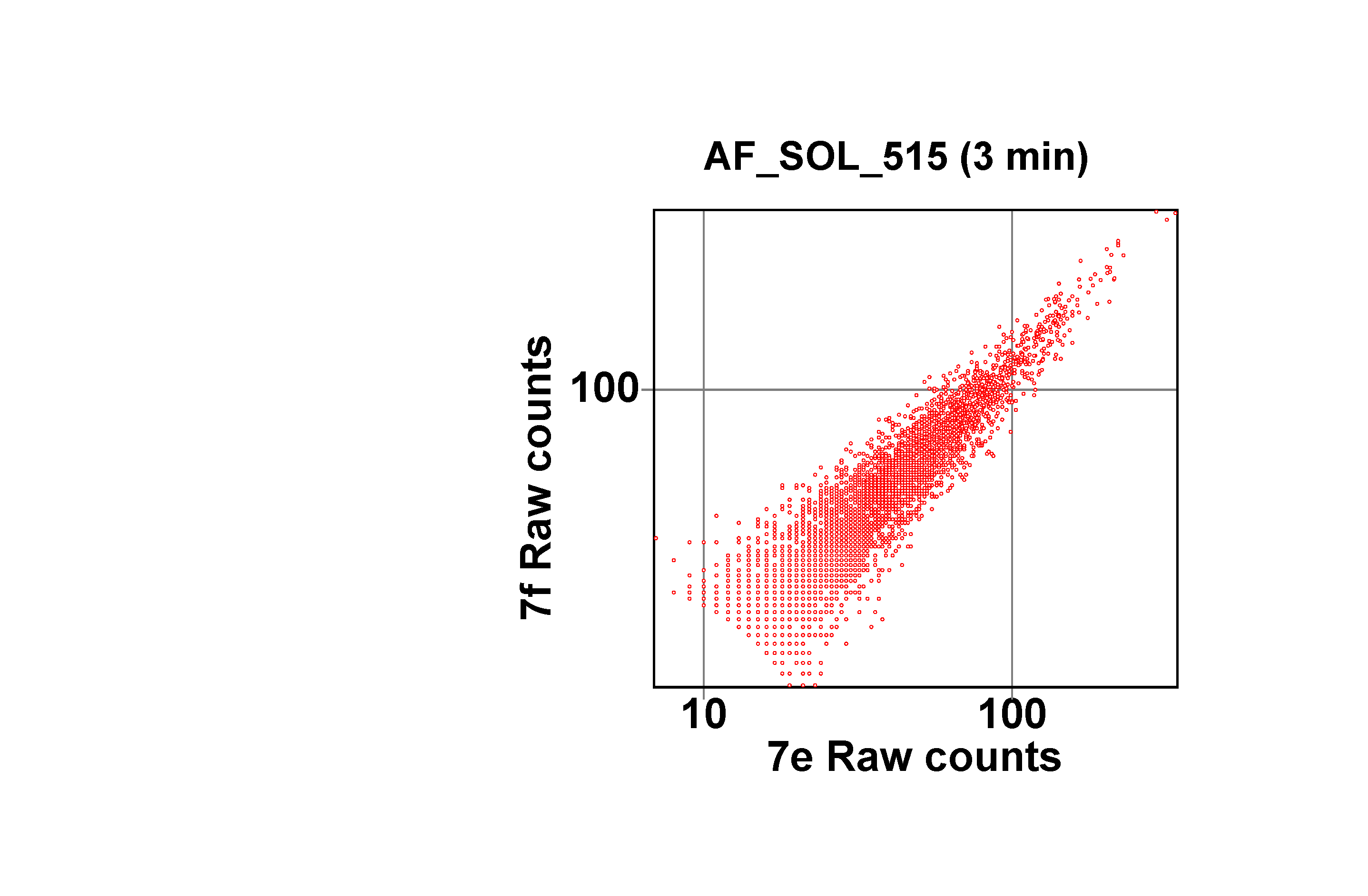

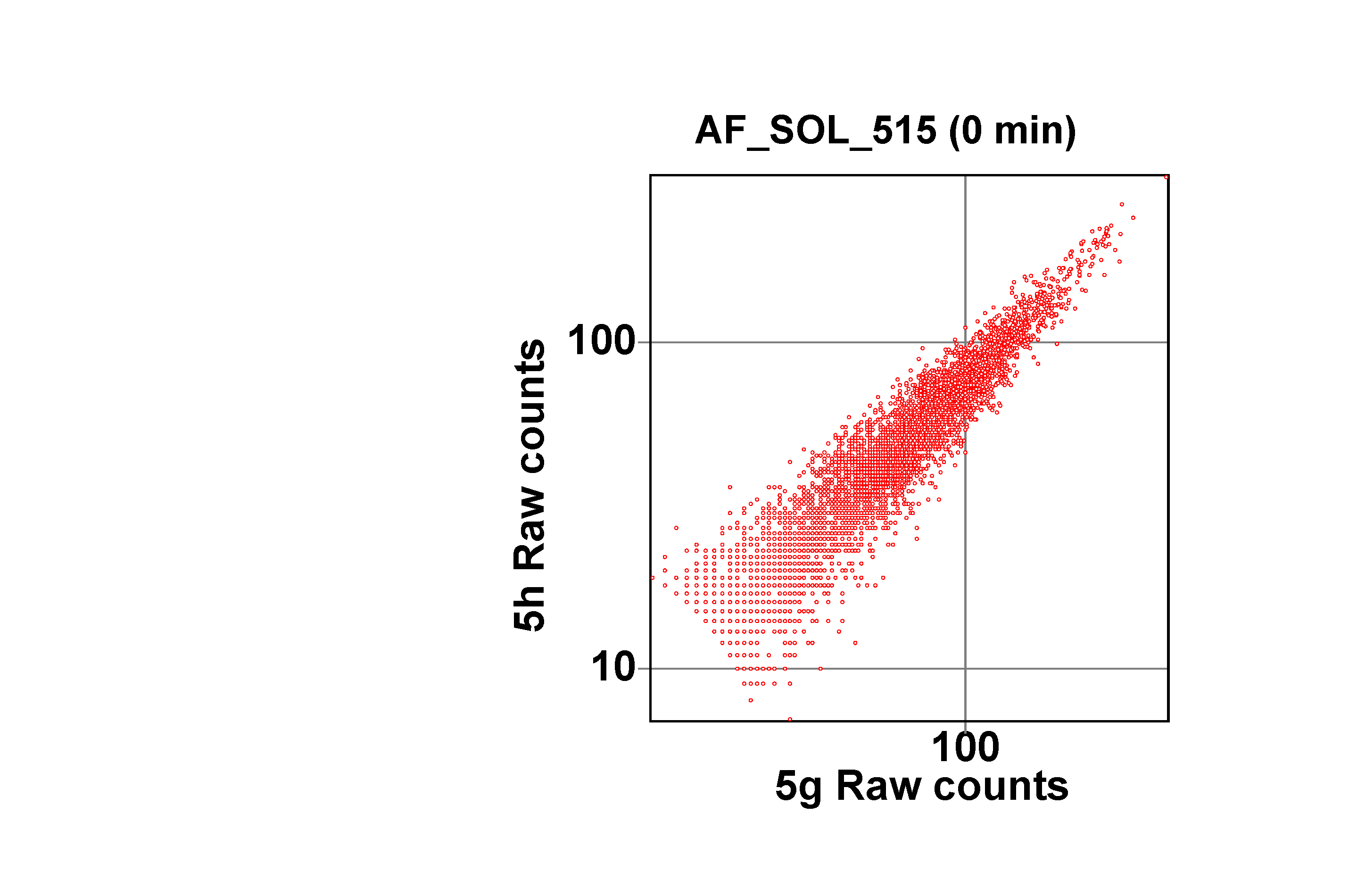


b) Correlation data for AF_SOL_516 (unc-22A variant library).

Correlation plots for between PCR replicates:


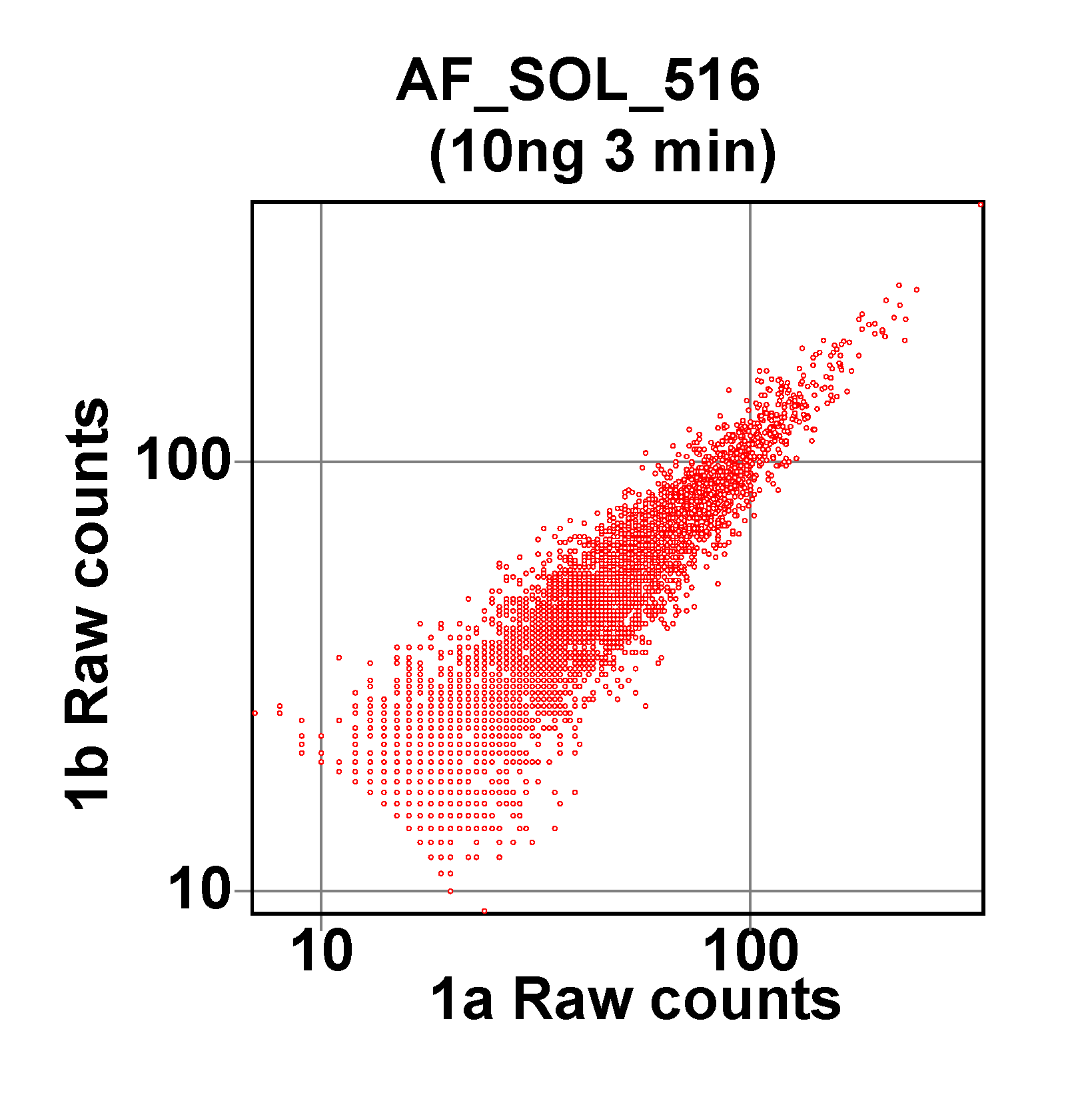

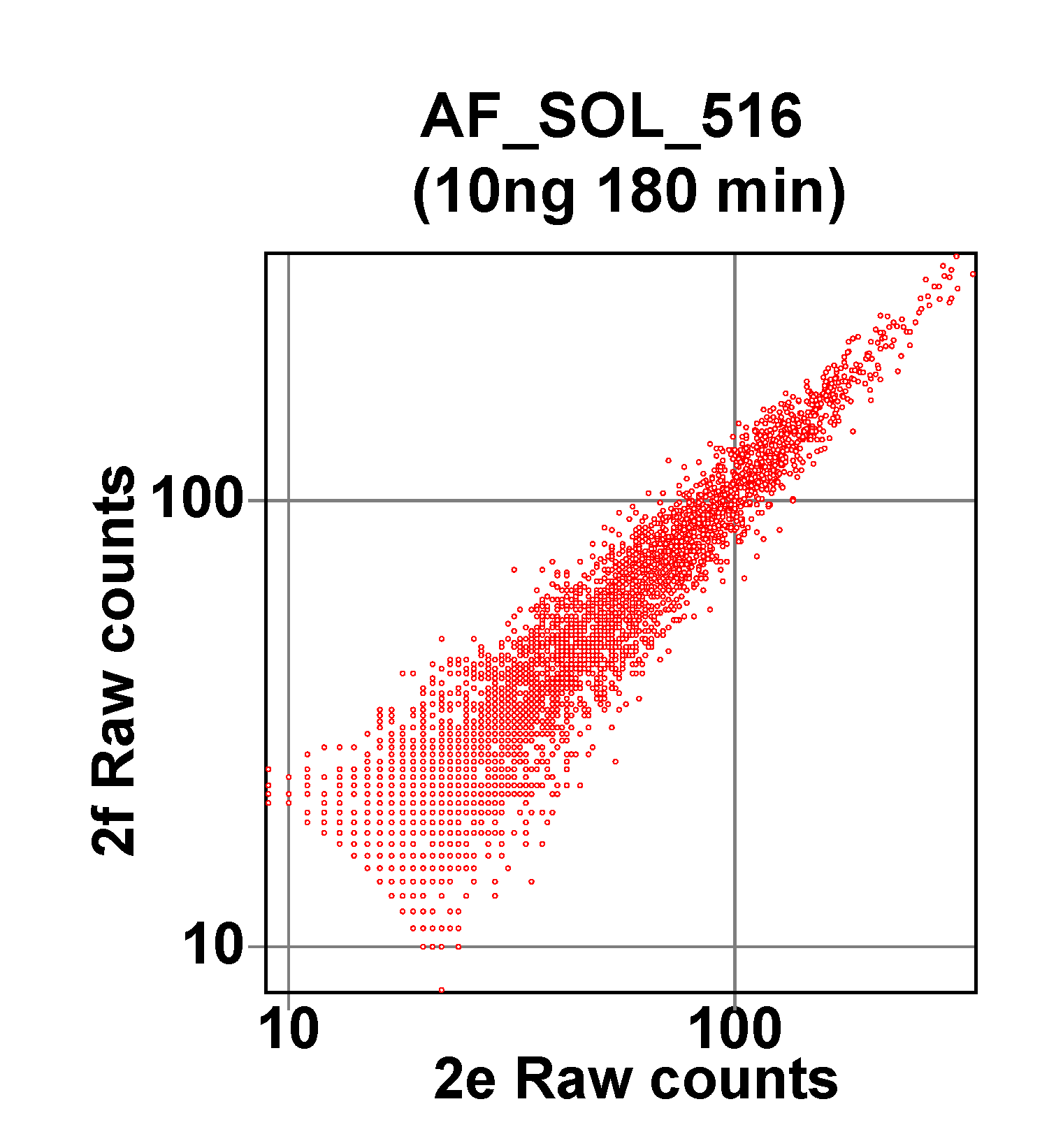

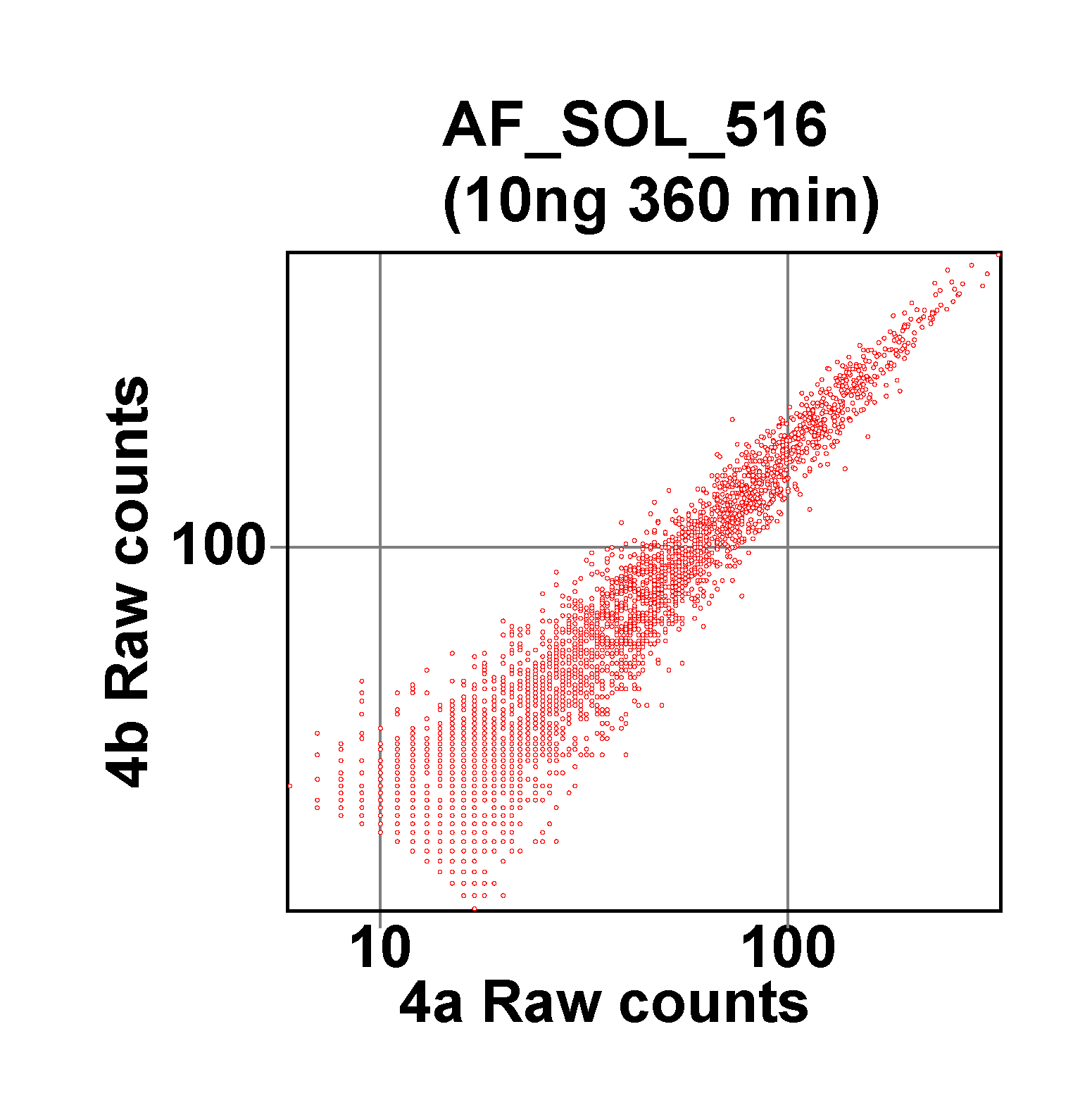

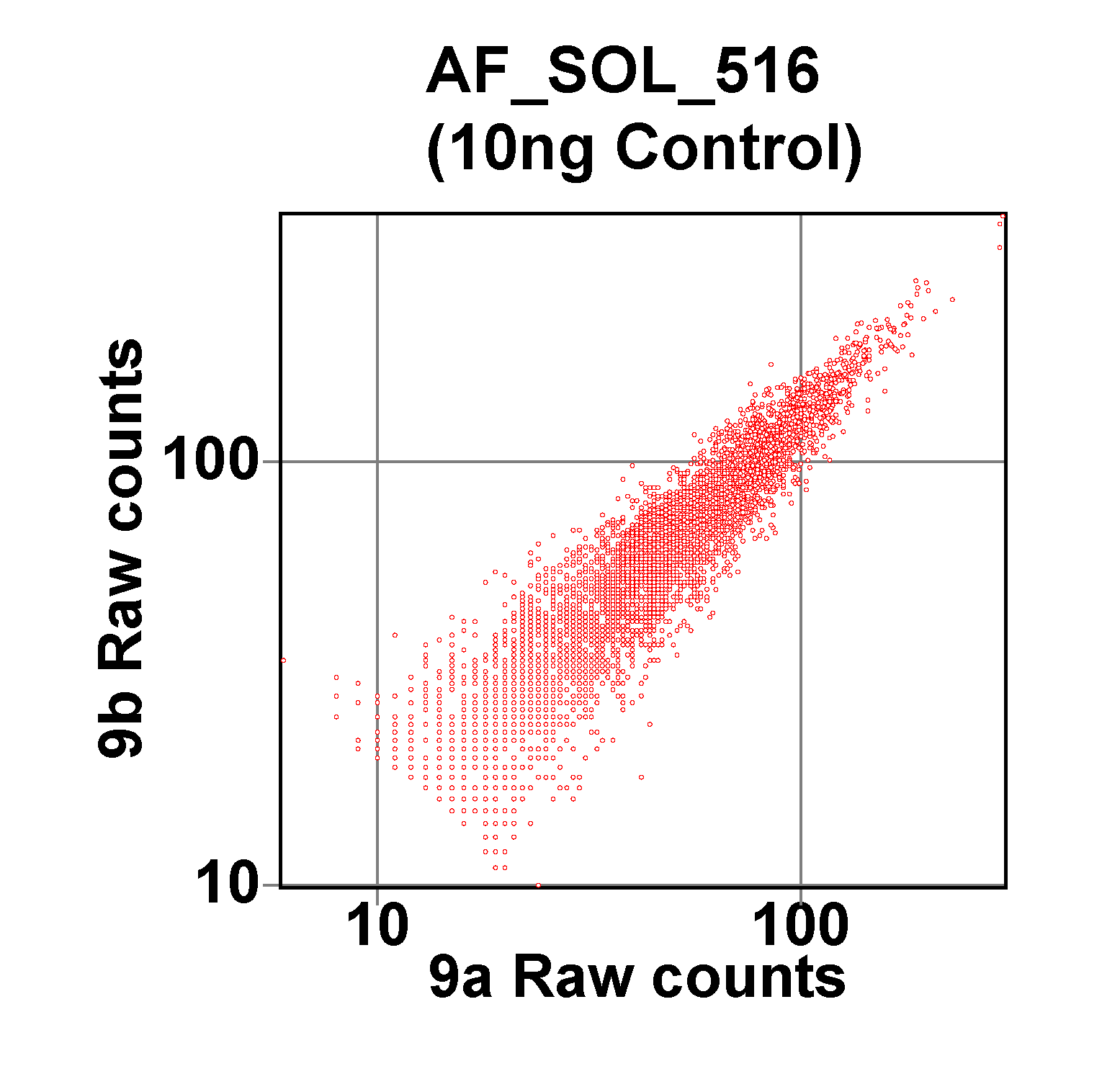


Correlation plots between different experiments:


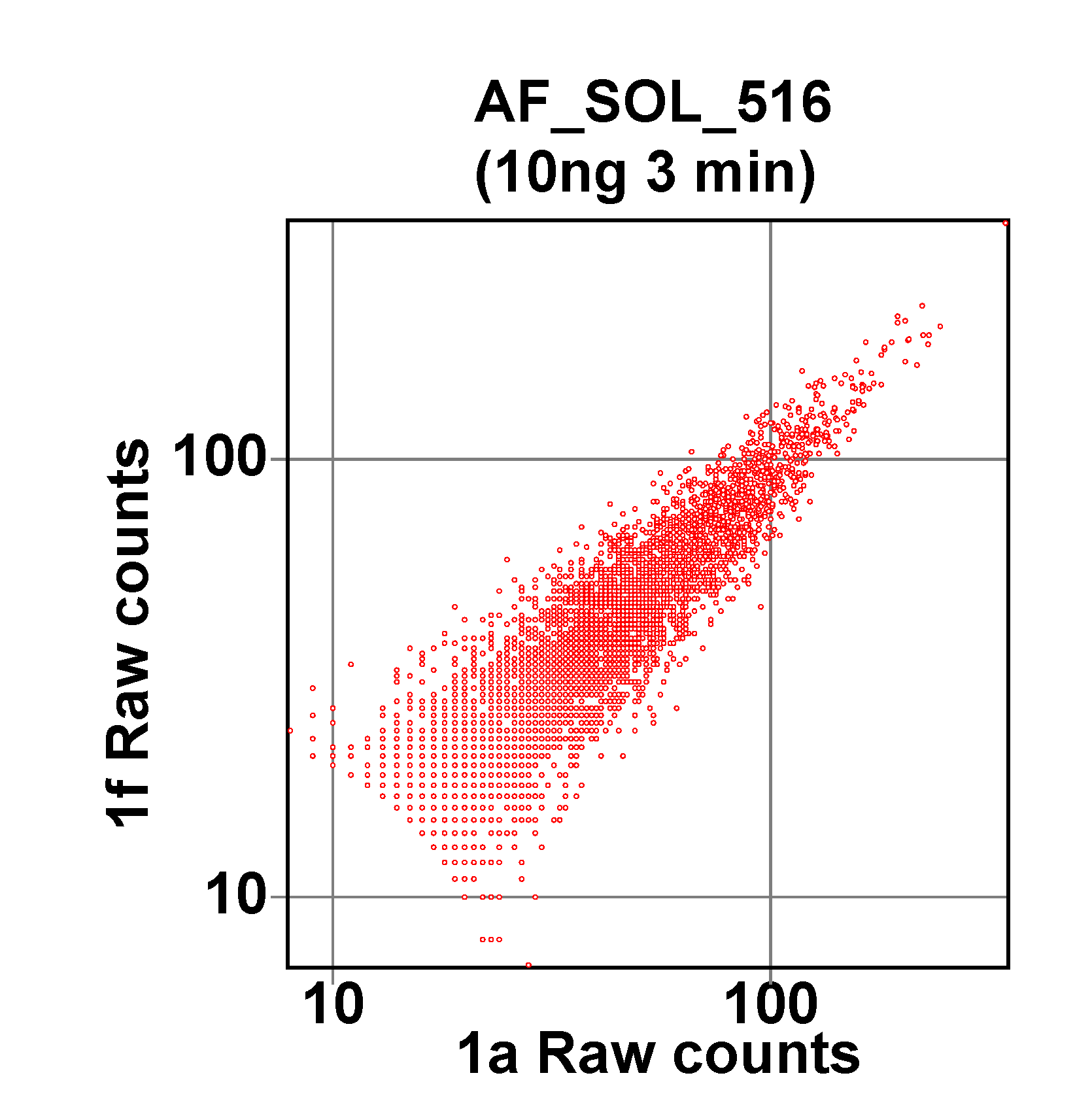

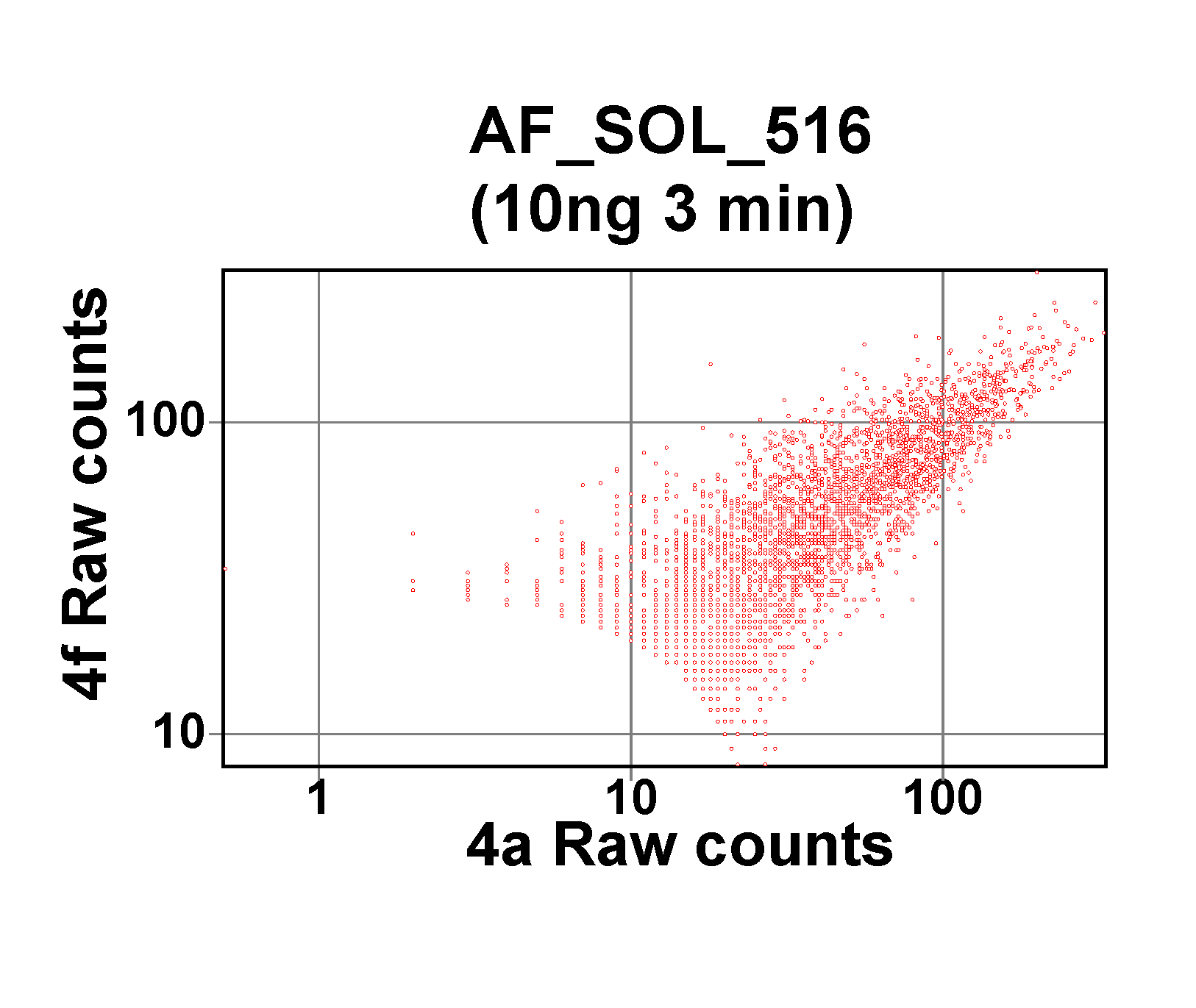

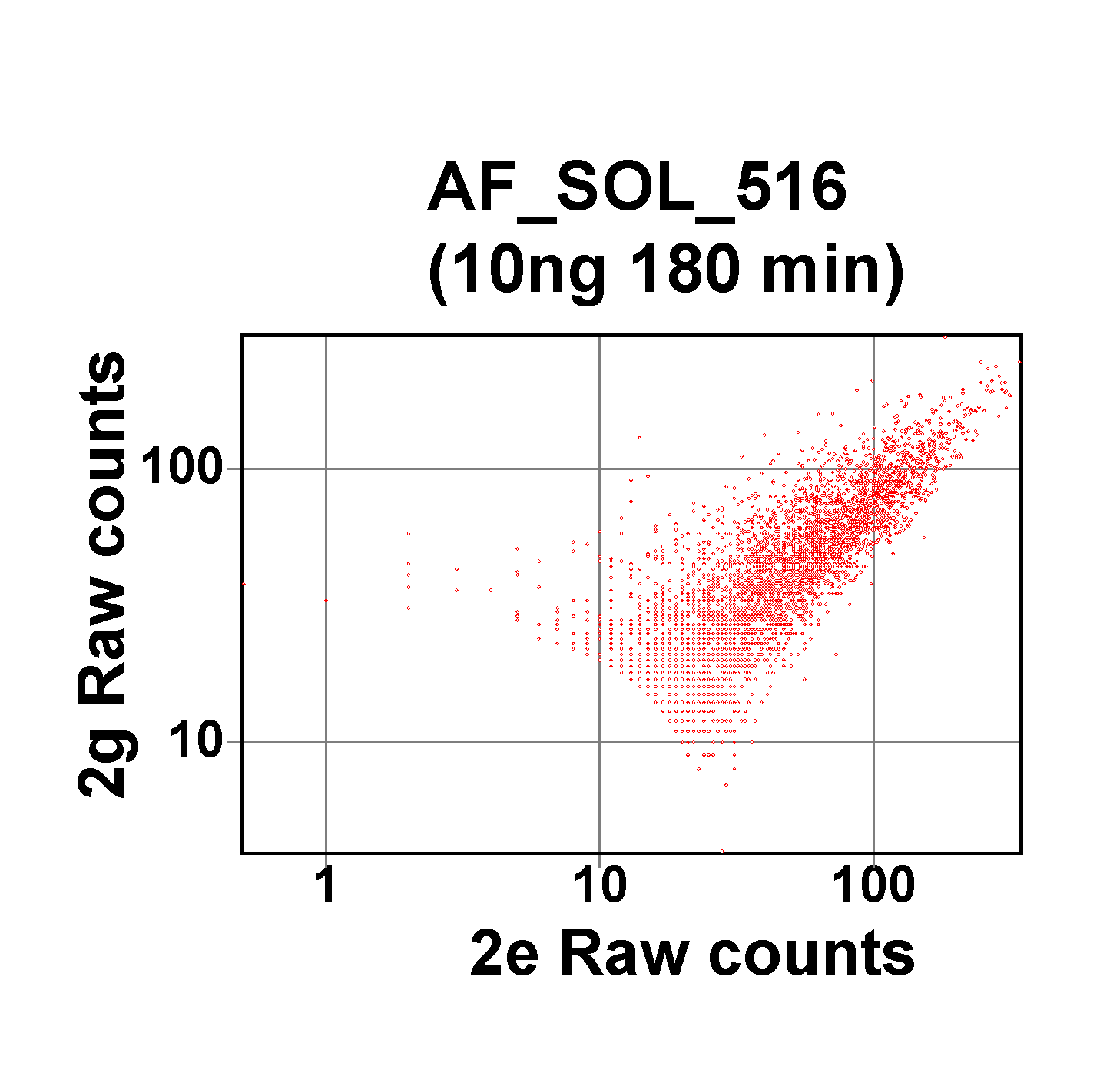


c) Correlation plots for AF_SOL_513 (ps4 variant library).

Correlation plots between PCR replicates:


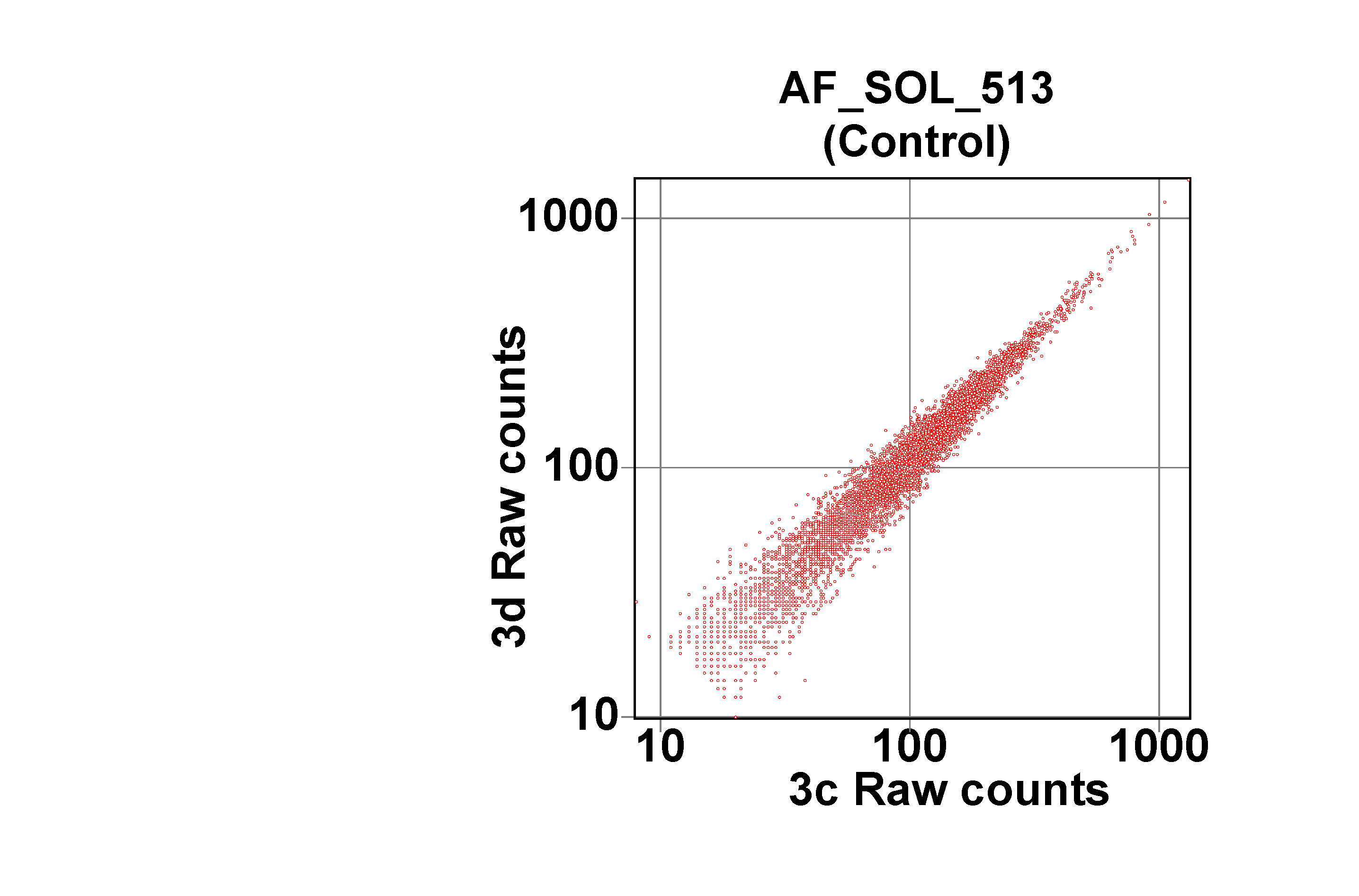

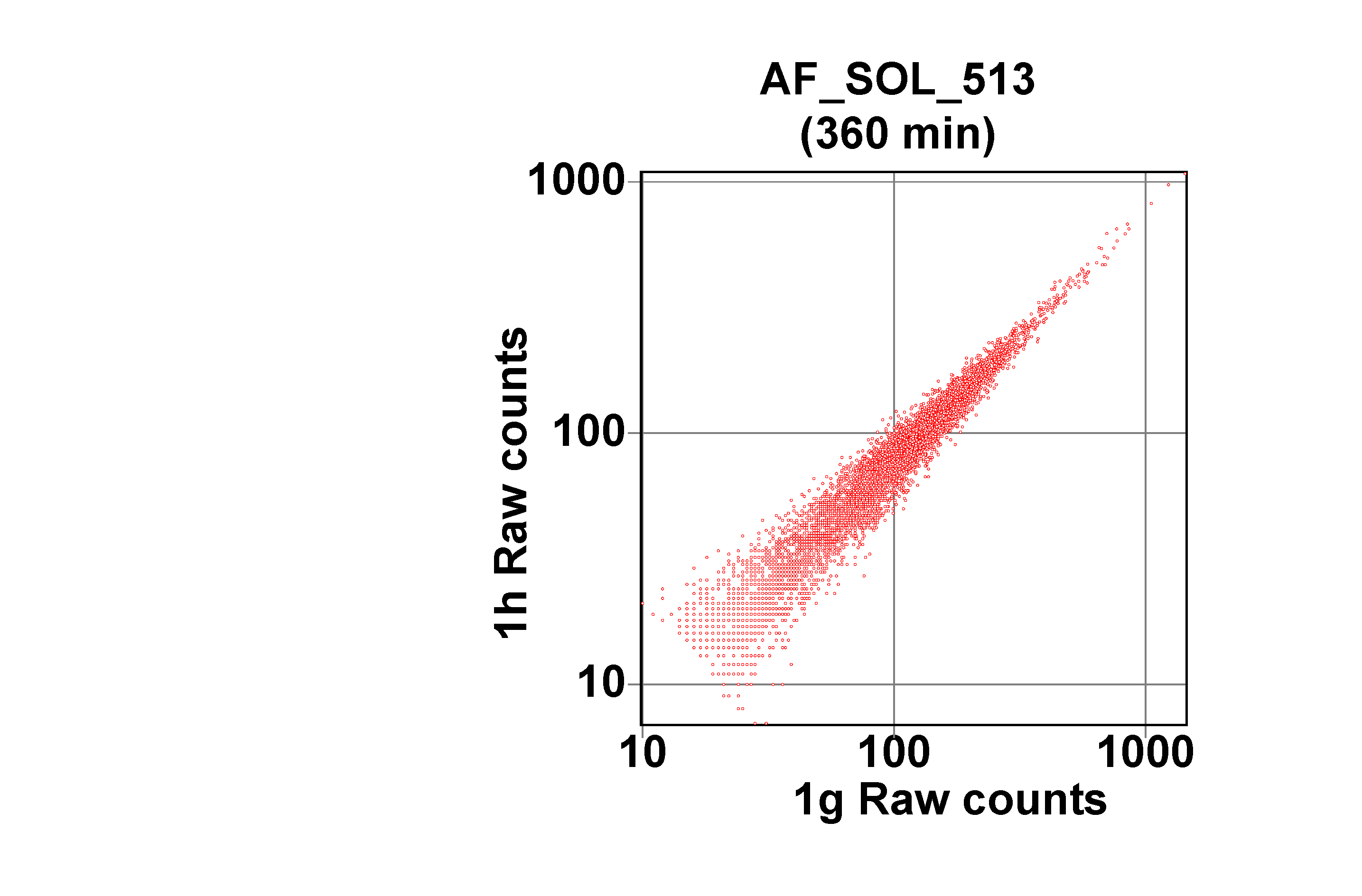


Correlation plots between different digests:


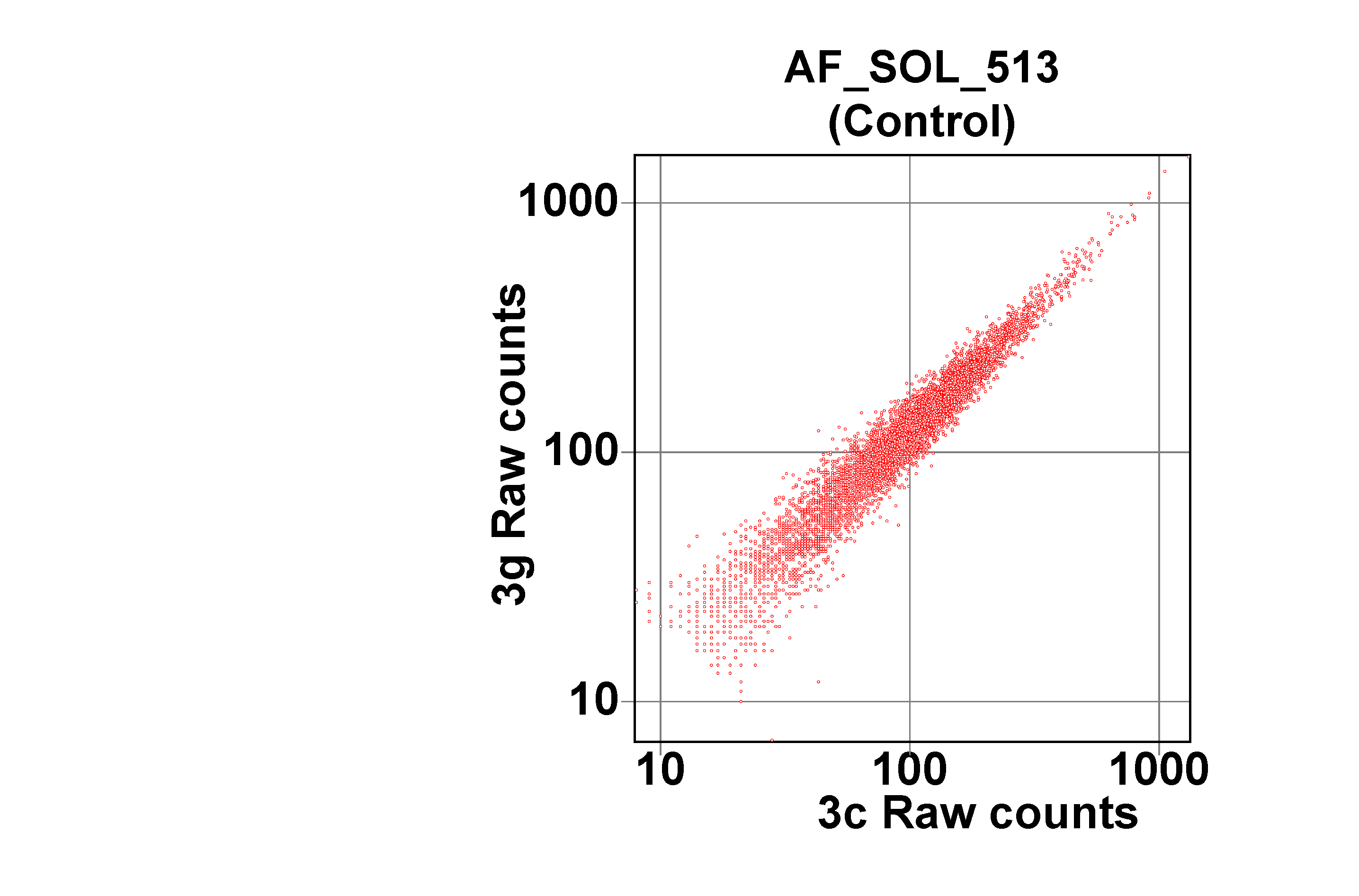

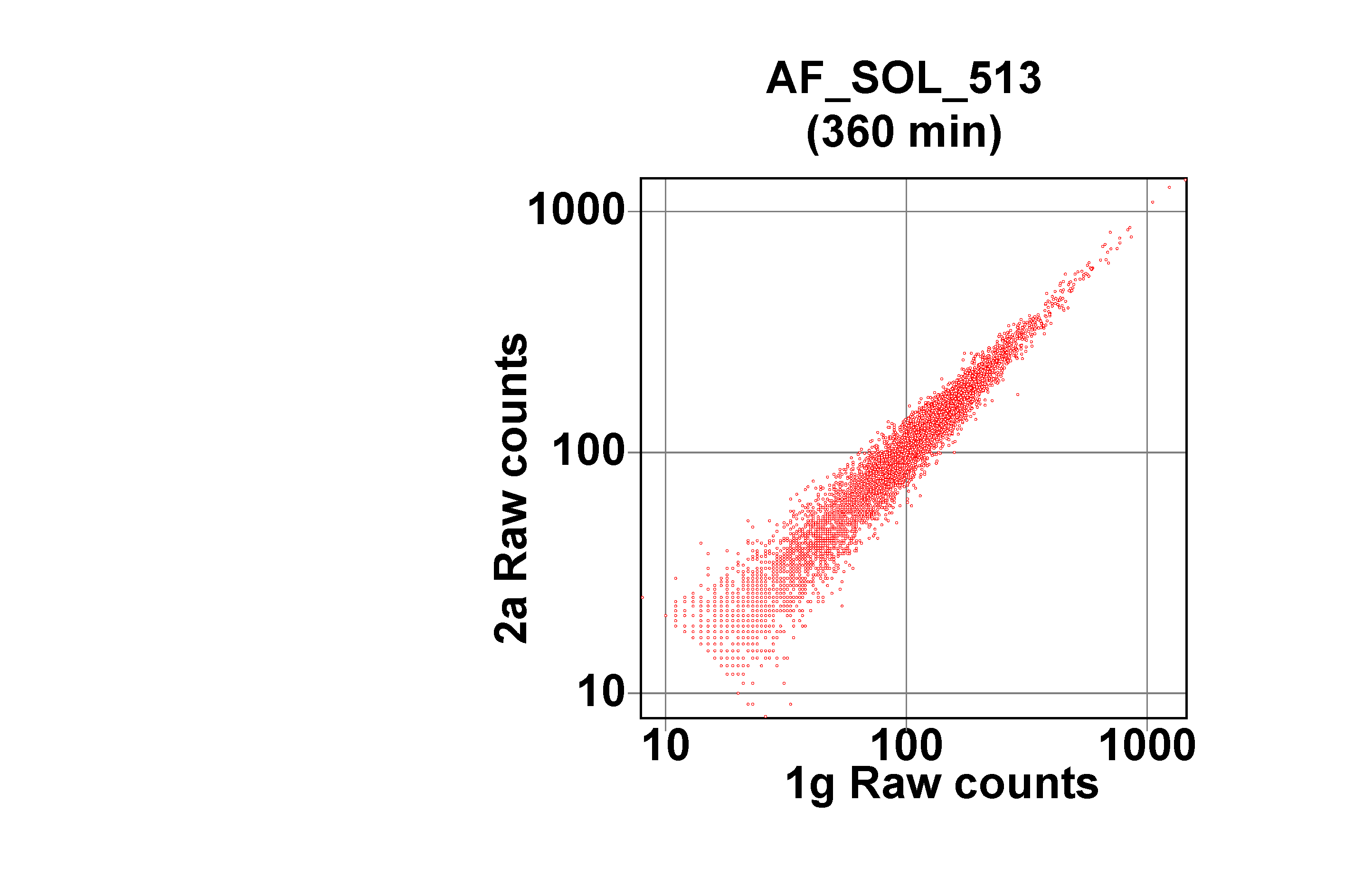


**Figure S7**: Histograms of log retentions. The histograms display the range and frequency of occurrence of the data for different experiments and different libraries.

1. Log retention distribution of the unc-22A random variant library RVL-1 (AF_SOL_516).


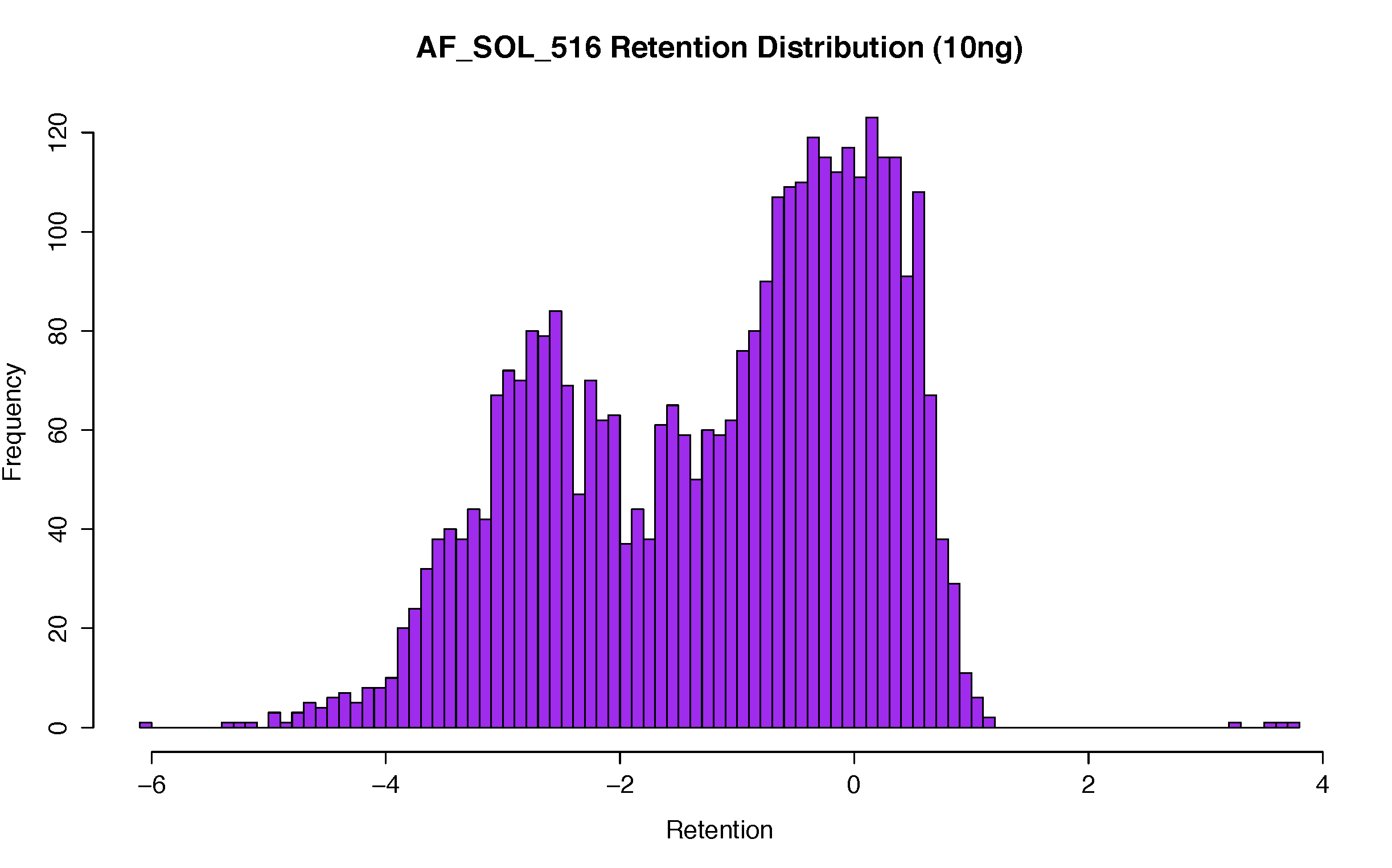


1. Log retention distribution of the unc-22A RVL-1 random variant library. (AF_SOL_515)


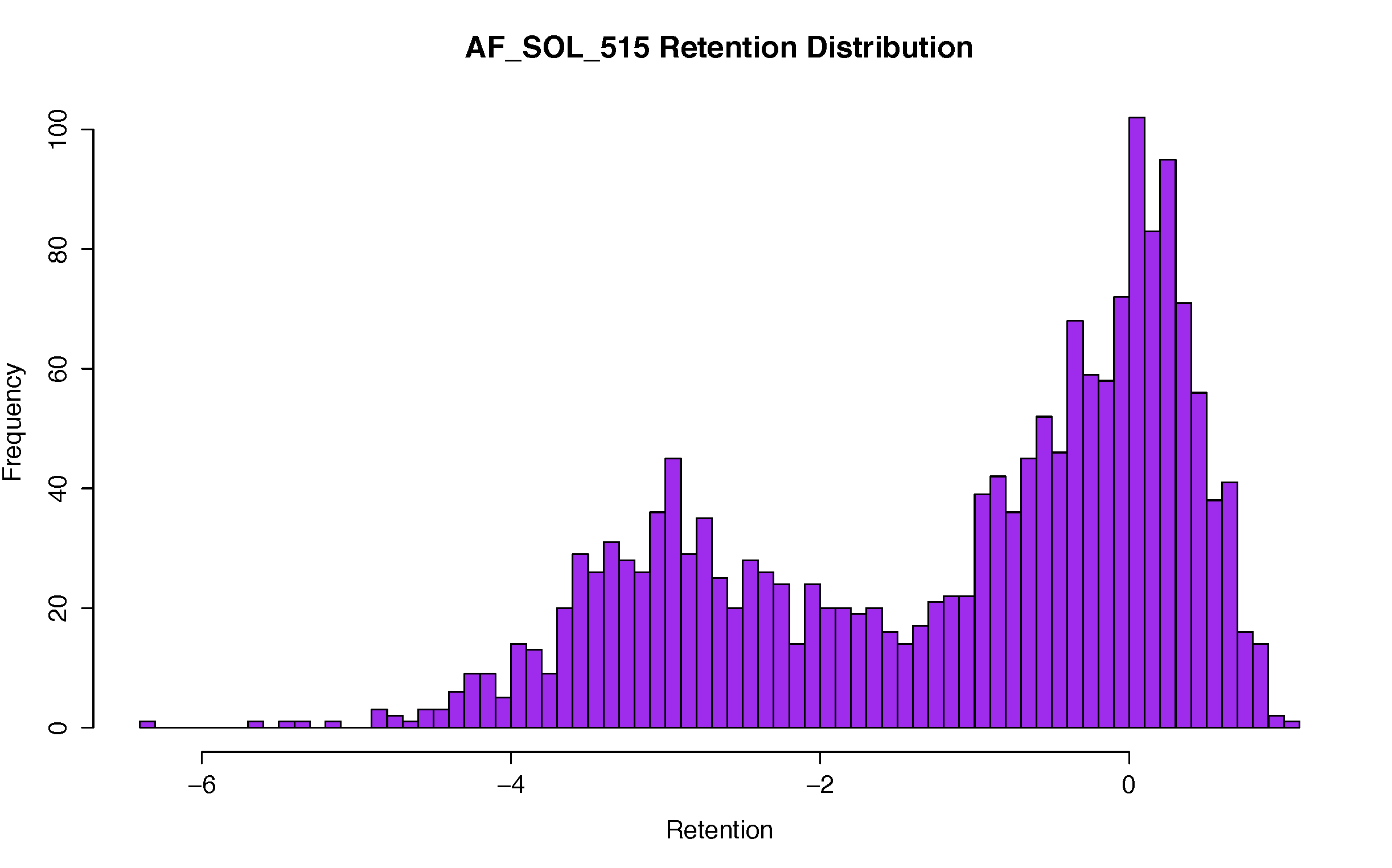


1. Log retention distribution of ps4 variant library. (AF_SOL_513)


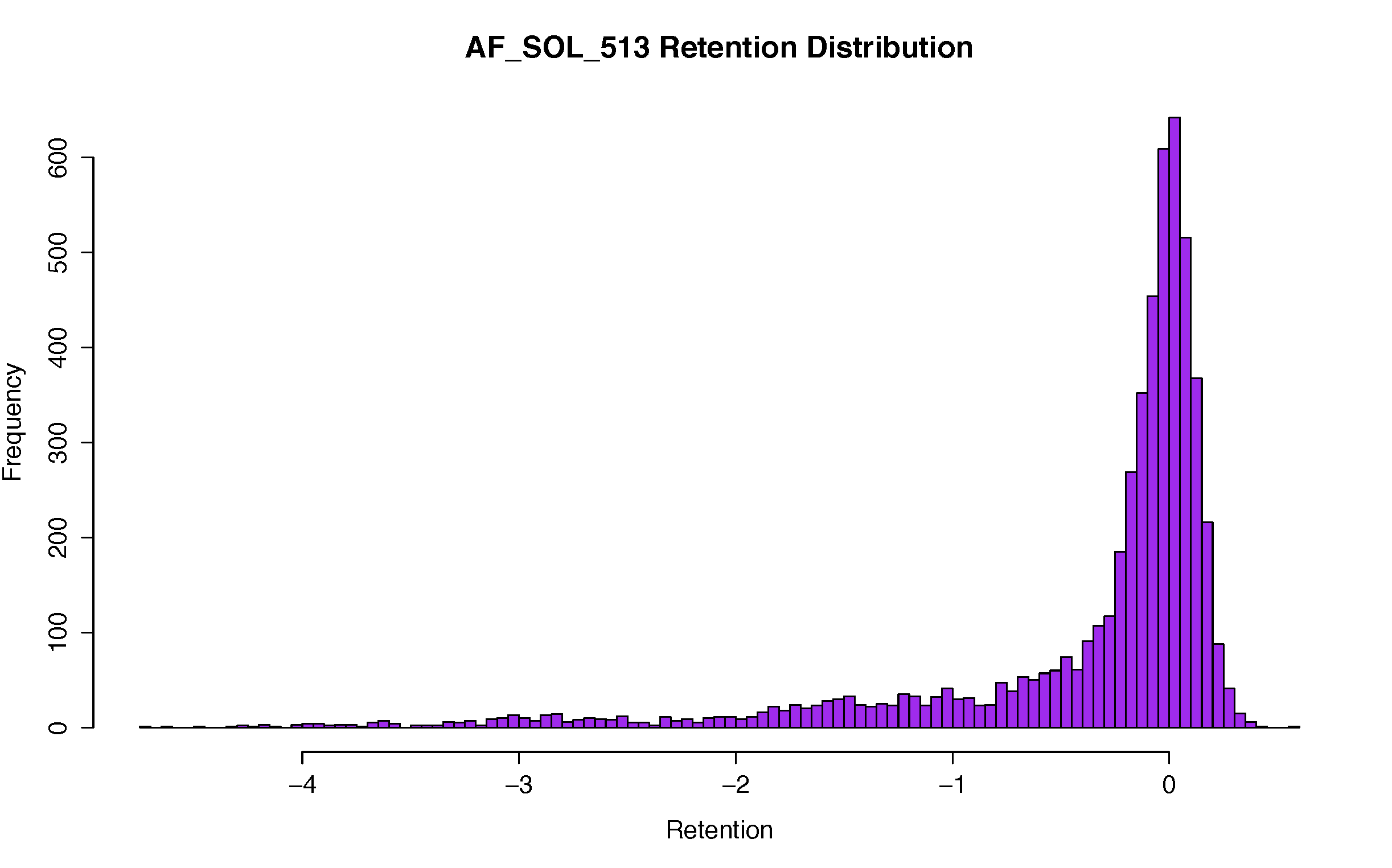


**Figure S8:** Cas9 *in vitro* experiments with simultaneous PCR and non-PCR based measurement of cleavage.

To obtain a rough assessment testing the validity of PCR-based measurement for cleavage of variants at a specific site (in this case position 11), we carried out , a number of assays in which cleavage of a predominant substrate was analyzed direct gel electrophoresis in parallel to analysis of the library by PCR and sequencing. Assays were carried out in larger volume (150 ul) to facilitate the gel analysis, with unc-22A RVL-2 library mixed with two additional plasmids bearing the unc-22A WT and C11G variants respectively. The two added plasmids have been engineered to generate separable restriction fragments (size difference ~100 bp) to allow a comparison of cleavage for these sequences with amplification-based and non-amplification-based assays. The additional DNAs included in the assay are diagrammed in a, with digestion using NEB PvuI-HF to visualize the two plasmids separately on the subsequent gel. A fraction of the purified DNA (1 ul) is taken and used for sequencing as in Figure 2. To further assess possible PCR bias in the assay, the numbers of PCR cycles was varied as shown.

a) The conditions of the non-PCR/PCR Cas9 *in vitro* experiments.

| Sample number: | Enzyme concentration (ug): | gRNA concentration (ng): | DNA mix: | Incubation time: | Sequence ID: |
| --- | --- | --- | --- | --- | --- |
| 2058 | 4 | 200  (WT unc-22A gRNA) | Mix A2:  40 ng unc-22A WT (pb_FU-1515), 40 ng unc-22A C11G (pb_FU-63), 35 ng unc-22 RVL-2 | 420 minutes | **AF_SOL_547:**  6 pcr cycles files: 1g-lo-420_S7_L001_R1_001_OUTPUT_AF_SOL_547.fasta, 1h-lo-420_S8_L001_R1_001_OUTPUT_AF_SOL_547.fasta  10 pcr cycles files: 3c-lo-420_S13_L001_R1_001_OUTPUT_AF_SOL_547.fasta, 3d-lo-420_S14_L001_R1_001_OUTPUT_AF_SOL_547.fasta |
| 2059 | 4 | 200  (unc-22A C11G gRNA) | Mix A2 | 420 minutes | **AF_SOL_547:**  6 pcr cycles files: 2a-lo-420_S9_L001_R1_001_OUTPUT_AF_SOL_547.fasta, 2b-lo-420_S10_L001_R1_001_OUTPUT_AF_SOL_547.fasta  10 pcr cycles files: 3e-lo-420_S15_L001_R1_001_OUTPUT_AF_SOL_547.fasta, 3f-lo-420_S16_L001_R1_001_OUTPUT_AF_SOL_547.fasta |
| 2060 | N/A | N/A | Mix A2 | 420 minutes | **AF_SOL_547:**  6 pcr cycles files: 2c-con-420_S11_L001_R1_001_OUTPUT_AF_SOL_547.fasta, 2d-con-420_S12_L001_R1_001_OUTPUT_AF_SOL_547.fasta  10 pcr cycles files: 3g-con-420_S17_L001_R1_001_OUTPUT_AF_SOL_547.fasta, 3h-con-420_S18_L001_R1_001_OUTPUT_AF_SOL_547.fasta |

b. Graphical representation of Cas9 assays:
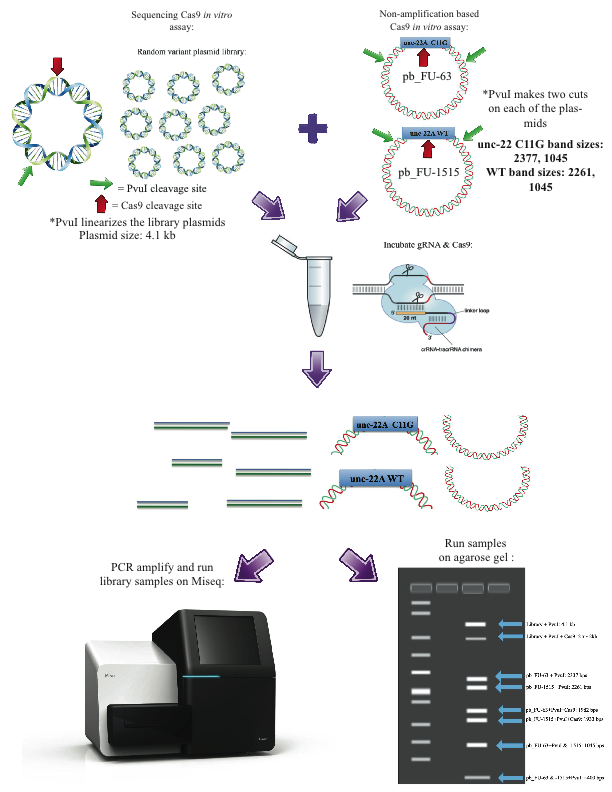


c) An experiment equivalent to Figure 2 using unc-22A library RVL-2 (AF_SOL_547). These assays were done with 6 cycles for PCR1 and 6 cycles for PCR2. Patterns are similar to those with increased PCR cycles (e.g. Figure S8d).
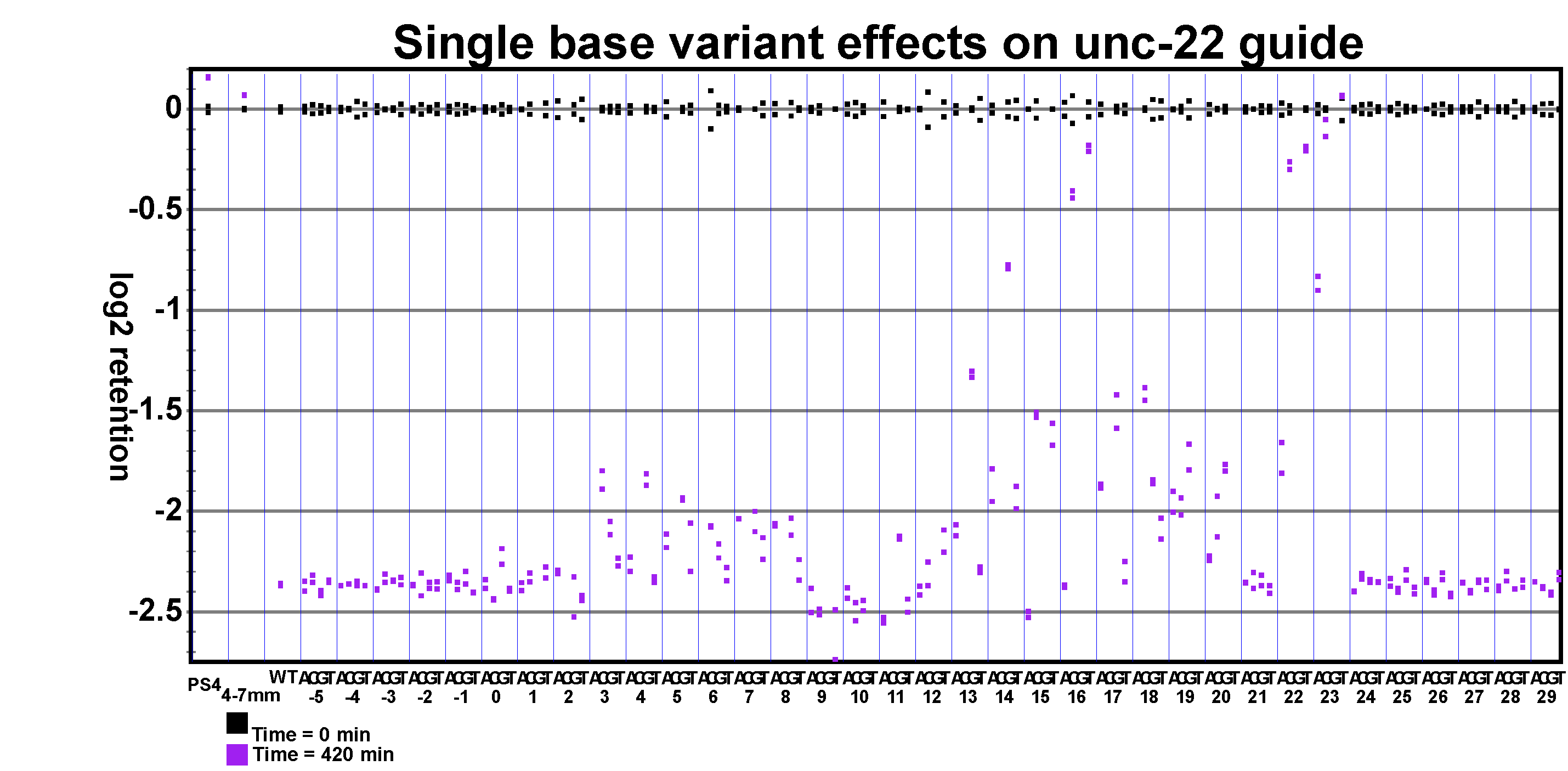

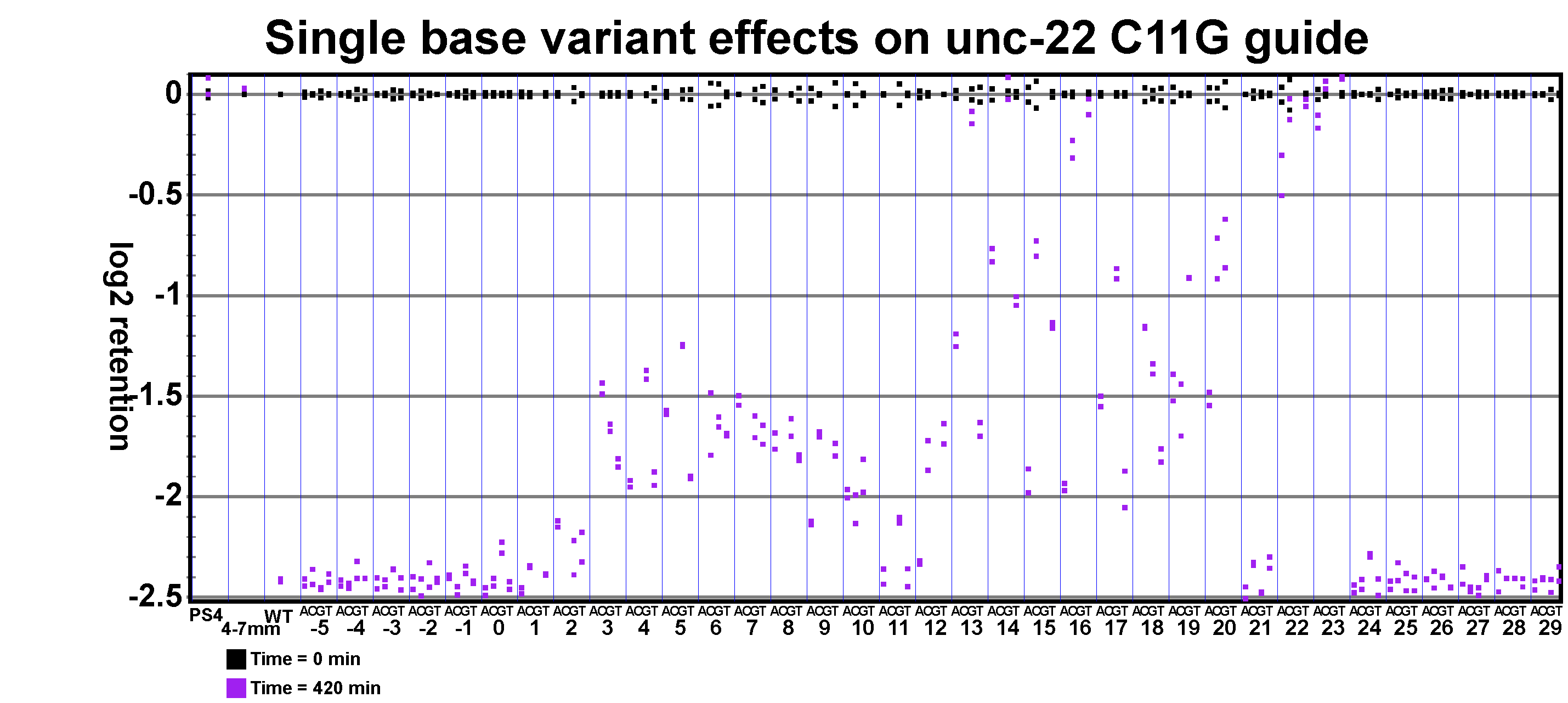


d) An experiment equivalent to Figure 2 using unc-22A library RVL-2 (AF_SOL_547). These assays were done with 6 cycles for PCR1 and 10 cycles for PCR2.
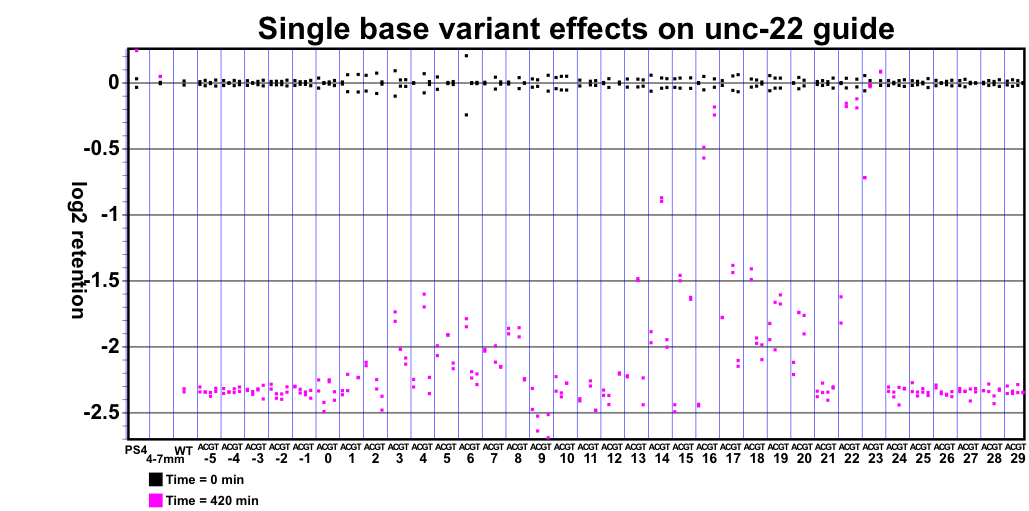

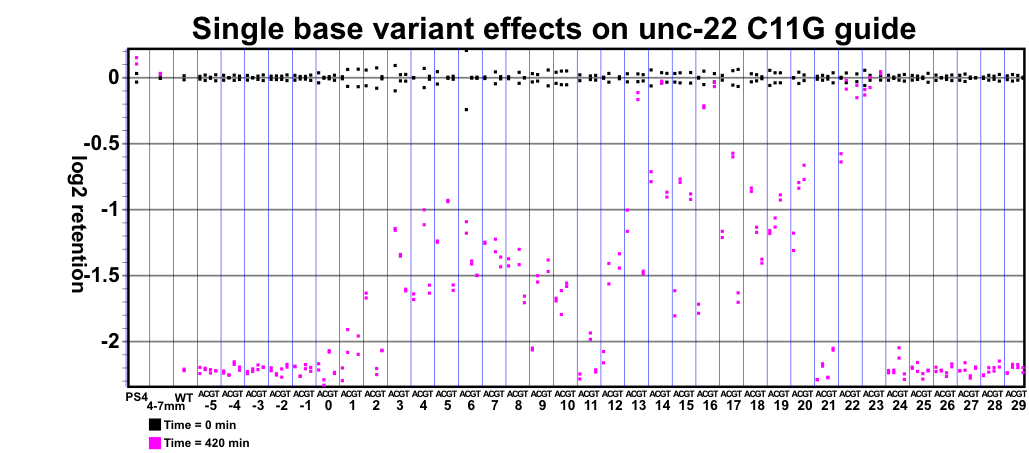


e) Agarose gel showing cleavage efficiencies of predominant cleavage substrates included in incubations for panel 8d.

Lanes 2058 (WT unc-22A gRNA) and 2059 (unc-22A C11G gRNA) show cleavage of the unc-22A WT and C11G mutant DNA plasmids in each sample. Bands labeled +Cas9 indicate cleaved products. The resulting cleavage efficiencies mirror the results of the sequencing experiments shown in Figure S8c-d.


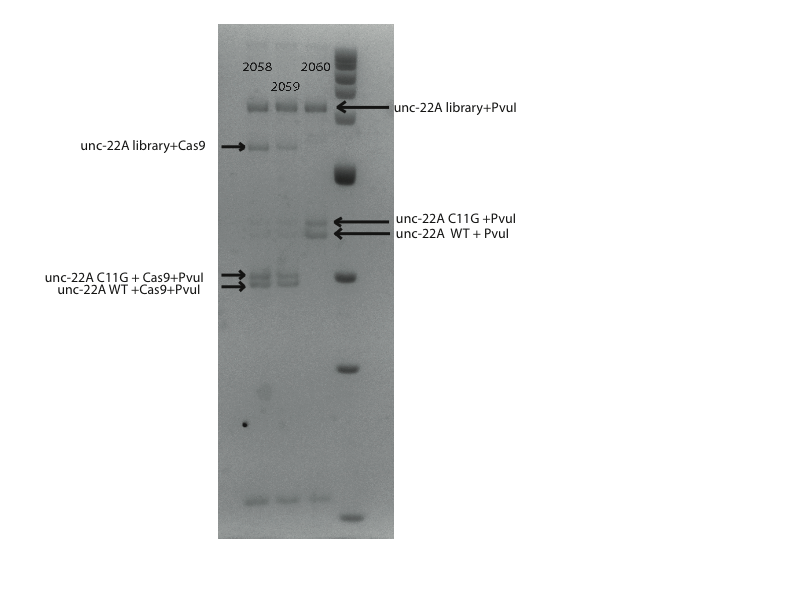


**Figure S9:**

To further investigate the effects of PCR on the Cas9 in vitro cleavage assay, equivalent experiments to Figure 2 and S8 were performed with 6 and 18 amplification cycles for PCR1. The patterns are similar despite more than doubling the cycle count for PCR1.

| Library: | Enzyme concentration (ug): | gRNA concentration (ng): | Sequencing platform: | Incubation time: | Sequence ID: |
| --- | --- | --- | --- | --- | --- |
| unc-22 RVL A  + WT unc-22A gRNA | 0.2 | 25 | Miseq | 420 minutes | **AF_SOL_550:**  6 pcr1 cycles; 4 pcr2 cycles files: (1a, 1b, 1c, 1d) -lo-420_S3_L001_R1_001_OUTPUT_AF_SOL_550.fasta  **AF_SOL_551:**  16 pcr1 cycles; 4 pcr2 cycles files: (1a, 1b, 1c, 1d) -lo-420_S1_L001_R1_001_OUTPUT_AF_SOL_551.fasta |
| unc-22 RVL A+  unc-22A C11G gRNA | 0.2 | 25 | Miseq | 420 minutes | **AF_SOL_550:**  6 pcr1 cycles; 4 pcr2 cycles files: (1e,1f,1g,1h) -lo-420_S5_L001_R1_001_OUTPUT_AF_SOL_550.fasta  **AF_SOL_551:**  16 pcr1 cycles; 4 pcr2 cycles: (1e,1f,1g,1h) -lo-420_S1_L001_R1_001_OUTPUT_AF_SOL_551.fasta |
| unc-22 RVL A +  no gRNA | N/A | N/A | Miseq | 420 minutes | **AF_SOL_550:**  6 pcr1 cycles; 4 pcr2 cycles files: (2a, 2b, 2c, 2d)-con-420_S9_L001_R1_001_OUTPUT_AF_SOL_550.fasta  **AF_SOL_551:**  16 pcr1 cycles; 4 pcr2 cycles files: (2a, 2b, 2c, 2d) -con_S10_L001_R1_001_OUTPUT_AF_SOL_551.fasta |

1. An experiment equivalent to Figure 2 with library unc-22A RVL-2 in 10 ul reactions with WT and unc-22 C11G mutated gRNA (AF_SOL_550). [6 pcr1 cycles; 4 pcr2 cycles]


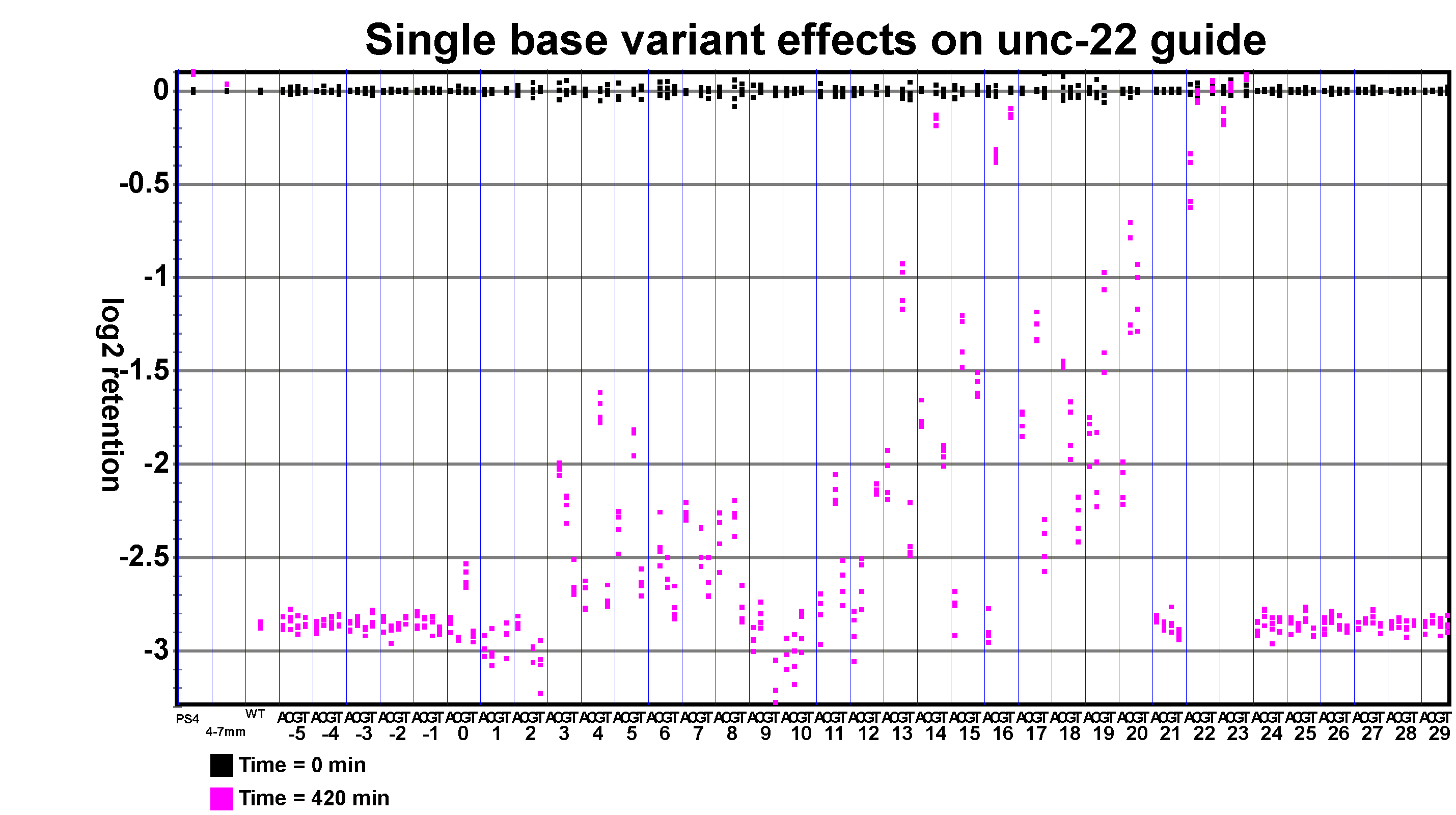

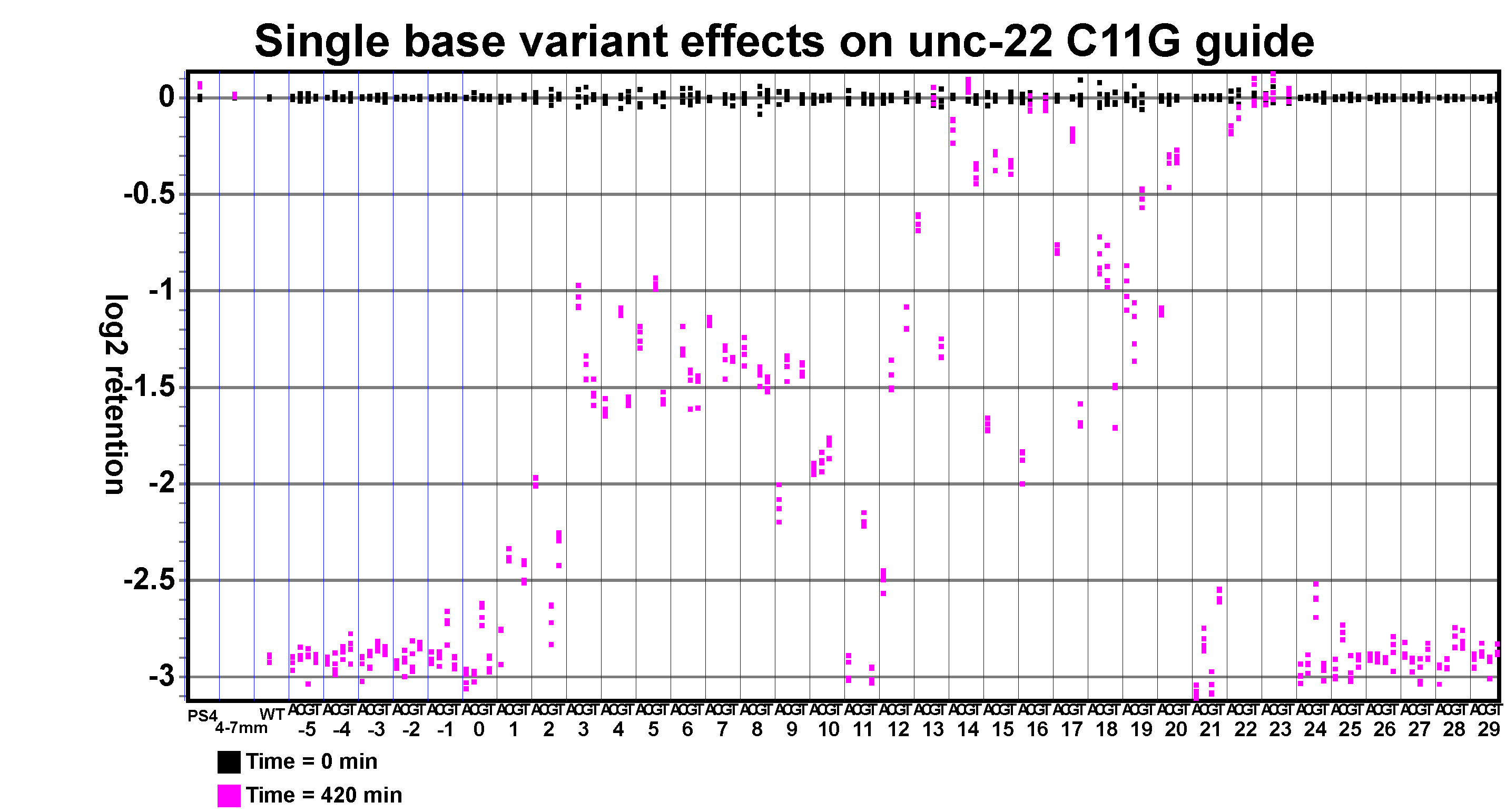


1. An experiment equivalent to Figure 2 with library unc-22A RVL-2 with WT and unc-22 C11G mutated gRNA (AF_SOL_551). [16 pcr1 cycles; 4 pcr2 cycles]


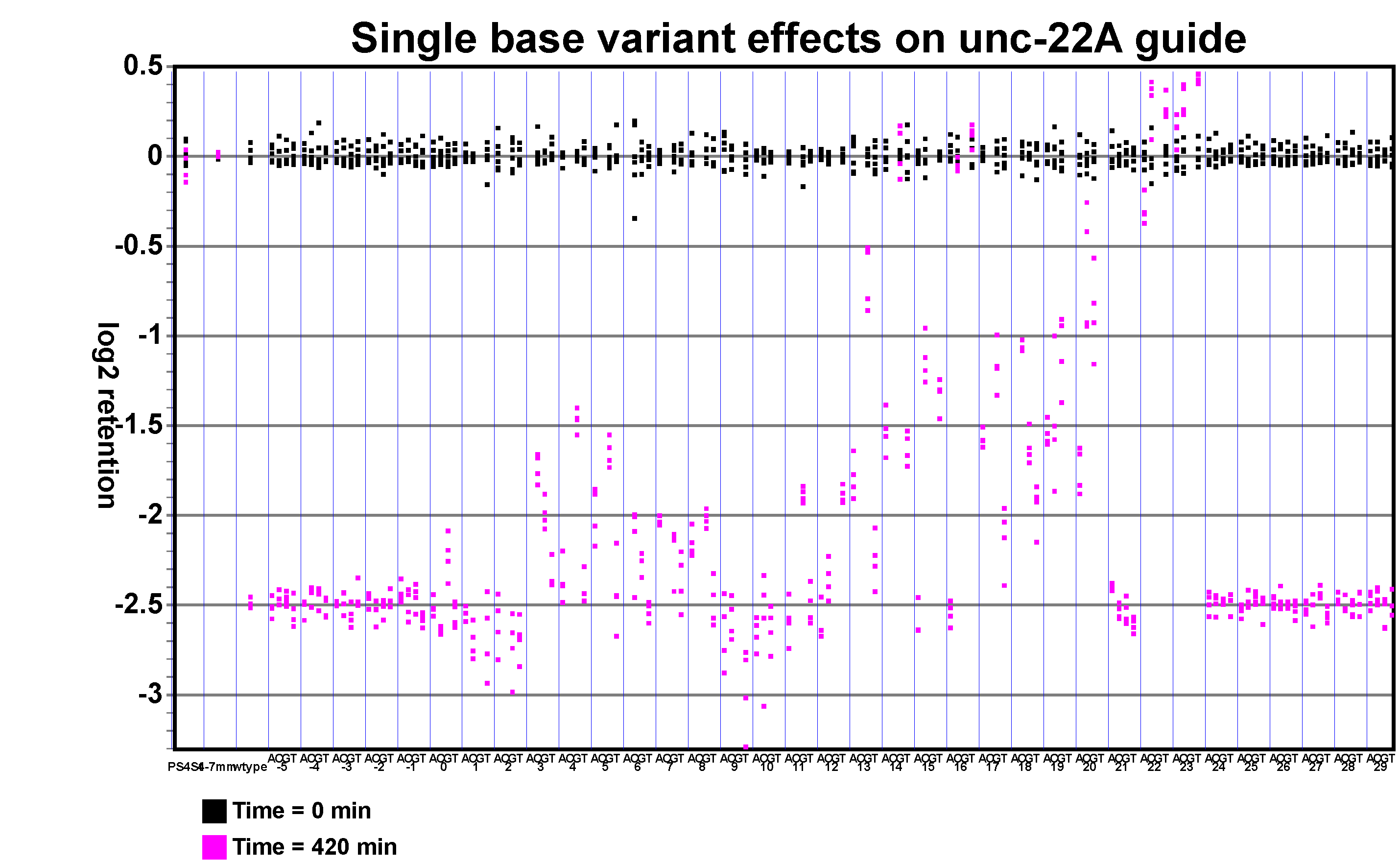


**
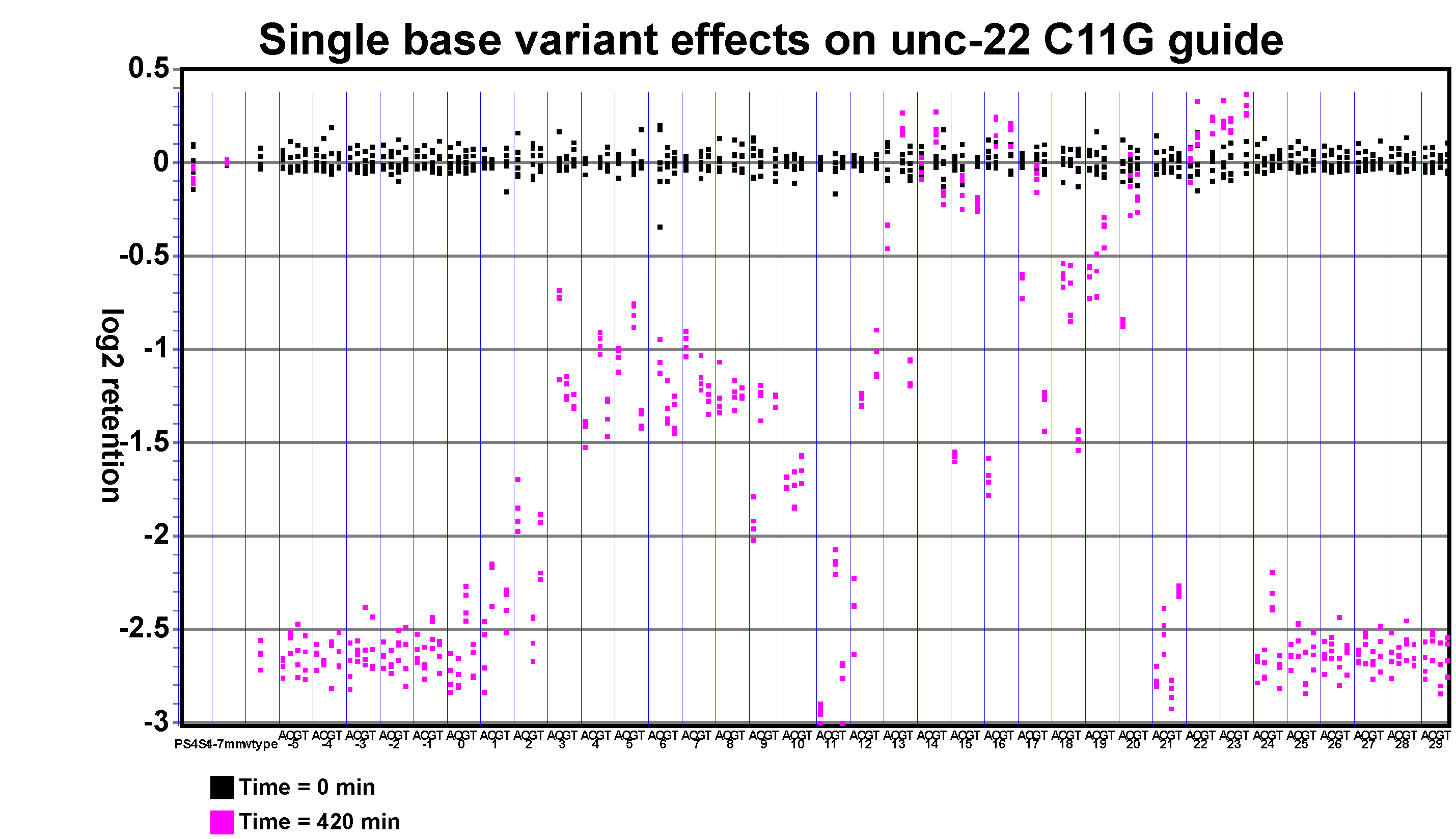
**

**Figure S10**: PCR-free assays for cleavage of plasmids carrying variants unc-22A G16T and T20A. Bands labeled +Cas9 indicate cleaved products.

a. **All experiments have 0.2 ug of Cas9 and 30 ng of plasmid DNA.**


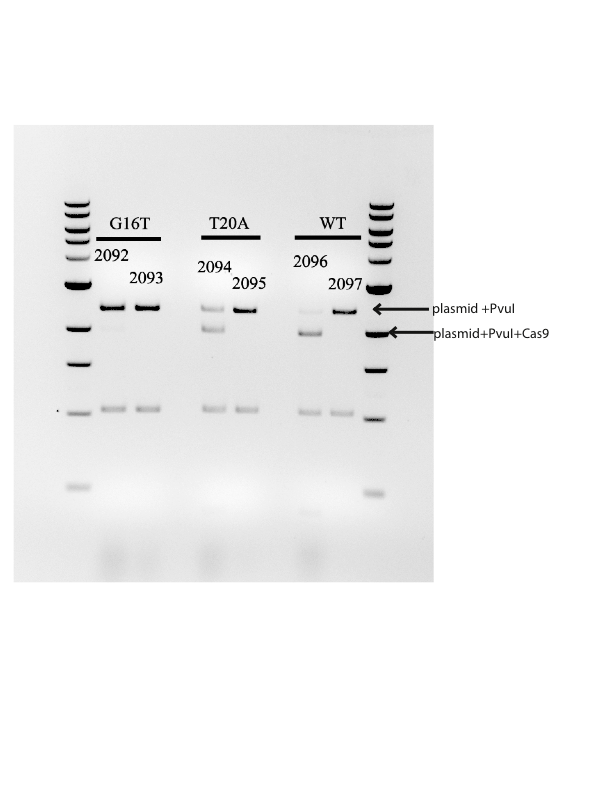


2092: unc-22A G16T plasmid + Cas9:unc-22A WT gRNA+PvuI

2093: unc-22A G16T plasmid+PvuI

2094: unc-22A T20A plasmid + Cas9:unc-22A WT gRNA+PvuI

2095: unc-22A T20A plasmid+PvuI

2096: unc-22A WT plasmid + Cas9:unc-22A WT gRNA+PvuI

2097: unc-22A WT plasmid+PvuI

b. **All experiments have 0.4 ug of Cas9 and 30 ng of plasmid DNA.**





2104: unc-22A G16T plasmid + Cas9:unc-22A WT gRNA+PvuI

2105: unc-22A G16T plasmid+PvuI

2106: unc-22A T20A plasmid + Cas9:unc-22A WT gRNA+PvuI

2107: unc-22A T20A plasmid+PvuI

2108: unc-22A WT plasmid + Cas9:unc-22A WT gRNA+PvuI

2109: unc-22A WT plasmid+PvuI

**Supplementary Methods:**

The use of high throughput sequencing-based assays universally entails the possibilities of noise and bias due to technical aspects of library construction, sequencing, and analysis.  We are aware of at least some of the potential sources of such noise and bias; as such, we have attempted to design our experiments and analysis to maximize both accuracy and robustness in measurement of relationships between target sequence and cleavage efficiency.  We describe the basis of these decisions below.

**Construction and complexities of libraries:**  Each library used in our analysis consists of a population of plasmids polymorphic in a 35nt stretch consisting of (i) a random six base 5’ “barcode”, (ii) a 20 base guide homology region and three base PAM sequence with variable numbers of mismatches to the consensus, and (iii) a random barcode of six random nucleotides.  The barcode sequences serve both to provide definitive information identifying each individual clone and (secondarily) to provide some information on potential effects of flanking sequence on cleavage efficiency.   Critically, we chose a library size so that the space of potential 5’+3’ barcode combinations (4^6)*(4^6)=~16M is only very sparsely occupied. With approximately 5000 clones in this space, we insure that >99.9% of potential barcode combinations are unused [1-(4^6)*(4^6)/5000.0 = 0.9997].  Despite the sparse population of barcode space, this library is large enough so that all single-base variants from the canonical (guide homology+PAM) sequence are covered by several independent instances in the library.

**Filtering of species with low read count:** Analysis of sequence datasets involves a filter step in which individual 35-mer sequences to be considered for calculation of retention must be represented in control (uncut) libraries with a minimum number of instances (in the case of these studies with ~10^6 reads per sample, the threshold chosen was n>=50.  The latter filter step removes numerous rare species likely to be due to sequencing errors, chimerism, and other confounding events.

**Redundant analysis:** A number of redundancies in the experimental analysis serve to ensure robustness in the measured effects on sequence retention.

First, each cleavage assay is repeated in a number of independent populations of DNA from a single library, with results for any species showing strong reproducibility between replicates (Figure S8-10).

Second, each variant is generally represented by several different species (with distinct 5’ and 3’ barcodes).  Having multiple species that are clearly distinct by both 5’ and 3’ barcode identities allows us to take a median value for each variant from among the matching species.  For the analysis of single mutants in the PAM sequence, each sequence variant was represented by at least three independent species with differences in both 5’ and 3’ barcodes.  Taking the median values serves to avoid any substantial outlier effects on any individual species.

Third, we constructed duplicate libraries for the unc-22 target sequence with different sets of clones (and hence different combinations of barcodes to represent each species).  Profiles of point mutational effects were essentially identical for the different libraries.

Fourth, we note that overall profiles of cleavage were determined over a range of times, enzyme, reaction DNA concentrations, and PCR cycle numbers.  While some differences in relative effects are observed as a function of experimental conditions, the overall conservation of relationships argues for the veracity of the underlying assay.

Fifth, we have carried out a number of independent assays using gel electrophoretic separation of cleavage products for mixed substrates to estimate relative cleavage frequencies.  As such experiments involve somewhat different experimental conditions than the original library-only assays, we “spike” a complex library into these assays at a low concentration in order to compare PCR-based sequence recovery and gel-based fragment observation in a uniform set of conditions.

**Estimates of Chimerism during PCR:** Chimeric PCR products can be produced in any reaction where defined 5’ and 3’ primers are used to amplify a spanned region from a template mixture with diversity at multiple intervening positions.  In the case of this analysis, PCR chimeras would be evident through the appearance of 5’-3’ barcode combinations that would, in general, be absent in the initial plasmid library.  A specific concern is that cut samples might lead to partial extension during a first round of PCR, annealing during a subsequent PCR round to an additional template, and further extension, resulting in chimerism specifically at the site of Cas9-gRNA cleavage.

A worst-case scenario for chimerism would result from a universal random pairing of left and right sequences from the initial library to produce the eventual PCR amplified pool.  At the other extreme, a lack of chimeric sequences would be evidenced by a unique and absolute pairing of 5’ and 3’ barcodes so that each 5’ barcode corresponds universally to a 3’ barcode and vice versa.  The latter expectation is also violated in cases where an individual 5’ barcode is paired with different 3’ barcodes (or vice versa) in the library, with such cases resulting in a distinct count distribution (and in general with different sets of gRNA-homology mutations in such bone fide cases of barcode coincidence).   Analysis of potential barcode-recombination events in such analysis give us an upper bound on possible chimerism events in the PCR amplification, with such upper bounds showing 0.6% of total reads at meeting these criteria as possible chimeras, and with evident sequencing errors other than PCR accounting for at least half of events that fell into this category. No substantial differences in potential chimeric read counts between cut an uncut samples were observed. The sparse utilization of barcode space, with <0.1% of potential barcode combinations actually used, serves to greatly decrease any confounding effects on observed retention values, so that chimerism effects on any observed retentions should at most be on the order of 0.001%, or well below the variances already present in the assay.

**Categorization of point sequencing error types in cleavage datasets:**  In analyzing data from Ilumina sequencing of variant libraries we found one error type to predominate, albeit at low frequencies of <<1% consistent with the high fidelity of Illumina machinery and reagents.  These errors were cases in which a small number of reads would be observed that match a plasmid represented in the initial library but for which a single base that was mutated (non-cannonical) in the guide RNA homology region was fortuitously “corrected” to the consensus sequence present in the vast majority of clusters on the sequence flow cell.  Likely to represent rare failures in cluster differentiation, any influence of such errors on eventual retention values was avoided as the resulting sequence species were too rare to meet the cutoffs for inclusion in the subsequent medians for analysis.
